# Navigating Antibacterial Frontiers: A Panoramic Exploration of Antibacterial Landscapes, Resistance Mechanisms, and Emerging Therapeutic Strategies

**DOI:** 10.1021/acsinfecdis.4c00115

**Published:** 2024-05-01

**Authors:** Krittika Ralhan, Kavita A. Iyer, Leilani Lotti Diaz, Robert Bird, Ankush Maind, Qiongqiong Angela Zhou

**Affiliations:** † ACS International India Pvt. Ltd., Pune 411044, India; ‡ CAS, A Division of the American Chemical Society, Columbus, Ohio 43210, United States

**Keywords:** antimicrobial resistance, multidrug resistance, antibiotics, antibacterial, bacterial infection

## Abstract

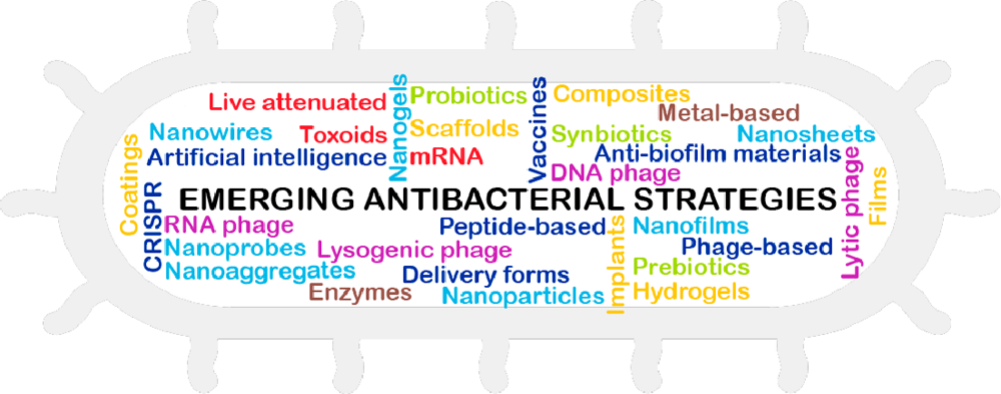

The development of effective antibacterial solutions has become paramount in maintaining global health in this era of increasing bacterial threats and rampant antibiotic resistance. Traditional antibiotics have played a significant role in combating bacterial infections throughout history. However, the emergence of novel resistant strains necessitates constant innovation in antibacterial research. We have analyzed the data on antibacterials from the CAS Content Collection, the largest human-curated collection of published scientific knowledge, which has proven valuable for quantitative analysis of global scientific knowledge. Our analysis focuses on mining the CAS Content Collection data for recent publications (since 2012). This article aims to explore the intricate landscape of antibacterial research while reviewing the advancement from traditional antibiotics to novel and emerging antibacterial strategies. By delving into the resistance mechanisms, this paper highlights the need to find alternate strategies to address the growing concern.

## Introduction

The emergence of drug-resistant bacterial strains and their associated challenges continue to be responsible for a sustained economic burden to the whole world, exemplified by the fact that the World Health Organization (WHO) declared antimicrobial resistance (AMR) as one of the top 10 primary health concerns affecting humanity.^1,2^ Bacterial infections comprise the majority of microbial infections because of their prevalence in diseases, public health impact, variability of virulence, development of resistance, and ease of transmission; therefore, antibacterial agents are the most common method to prevent and treat these infections. The spectrum of antibacterial activity encompasses a wide range of bacteria; however, certain bacterial strains, such as *Enterococcus faecium, Staphylococcus aureus, Klebsiella pneumoniae, Acinetobacter baumannii, Pseudomonas aeruginosa, Enterobacter* spp., and *Escherichia coli*—collectively called ESKAPEE—have garnered public interest because they are known to “escape” commonly used antibiotic treatment because of multidrug resistance (MDR).^3−6^ Data from the Centers for Disease Control and Prevention (CDC) for the year 2020 suggests that 6 out of the 18 listed antimicrobial-resistant bacterial threats, namely vancomycin-resistant *Enterococcus* (VRE), carbapenem-resistant *A. baumannii* (CRAB), methicillin-resistant *S. aureus* (MRSA), carbapenem-resistant Enterobacterales (CRE), multidrug-resistant *P. aeruginosa* (MDR-PA), and extended-spectrum β-lactamase (ESBL)-producing Enterobacterales, incur a collective cost of more than $4.6 billion annually.^7^ MRSA strains remain a leading cause of infections worldwide ranging from skin and soft tissue infections to more serious conditions, such as bacteremia and endocarditis. Because of the rise in resistant species, some bacterial infections have become public health threats. Constantly evolving antibiotic-resistant bacterial species emphasize the need for a systematic literature review and analysis in the antibacterial field.

In this report, we provide an overview of the current knowledge on antibiotic resistance, antibiotics, and antibacterial materials. Furthermore, we provide a landscape of the antibacterial field on the basis of data from the CAS Content Collection,^8^ the largest human-curated collection of published scientific knowledge, which has proven useful for quantitative analysis of global scientific publications. Our analysis focuses on mining the CAS Content Collection for recent documents (2012 onward) to uncover trends in journal and patent publications, the use of various substances, and to provide insights linking antibiotics with bacteria and disease indications. Additionally, we review the antibiotic resistance mechanisms, diverse classes of antibiotics, their modes of action, and emerging antibacterial strategies. The overarching aim of this report is to serve as a useful resource for understanding the current state of the field of antibacterials and global research efforts in this field.

## Antibiotic Resistance

According to WHO, a resistant organism is one that is not killed/inactivated upon completion of the entire course of treatment. According to data presented by the Centers for Disease Control and Prevention (CDC), more than 2.8 million antibiotic-resistant bacterial infections occur each year leading to >35 000 deaths per year.^9^ According to projections made by the World Bank,^10^ by the year 2050, 10 million people are projected to die because of MDR bacterial infections, thereby incurring a loss of up to USD 100 trillion to the global economy.^11^ Antibiotic resistance occurs when bacteria evolve to render existing antibiotics ineffective, thereby leading to difficult or ineffective treatment.^2,12,13^ While many factors have led to the rise in MDR, one significant factor is the inability of antibacterial drug discovery and development to keep pace with bacterial drug resistance. Over 100 antibiotics are available for treating bacterial infections, but overuse and misuse of antibiotics in both humans and livestock have also played a significant role in the rise of antibiotic resistance as it creates a selective pressure favoring resistant strains.^14,15^ Inadequate prescription practices, a rise in self-medication, and noncompliance with prescribed antibiotic regimens can exacerbate these issues. Highly resistant bacterial strains include various Gram-positive bacteria, such as *Enterococcus faecalis*, *E. faecium*, coagulase-negative *Staphylococci* (CNS), and methicillin-resistant *S. aureus* (MRSA), and Gram-negative bacteria, such as multidrug-resistant *Acinetobacter*, *Enterobacter*, *E. coli*, *P. aeruginosa, Klebsiella*, etc.^15−17^

Antibiotic resistance in bacterial species can be intrinsic or acquired. Intrinsic antibiotic resistance occurs primarily because of the inherent structural/genetic composition of a particular bacterial species, while acquired antibiotic resistance arises because of the gain of new genetic material or from a mutation arising in the bacterial genome to provide novel capabilities mediating survival in bacterial species.^18,19^ Mutations (contributing to acquired resistance) can be of several types—spontaneous, adaptive, and random, among others—that arise because of errors during replication or by inefficient repair of damaged DNA. In certain instances, selection pressure arising because of nonlethal antibiotics can result in hypermutations. In these cases, bacteria enter a state of high-mutation rate called the “hypermutable” state wherein they acquire mutations to survive. In certain cases, adaptive mutations can occur in nondividing or slowly dividing cells due to selection pressure. These mutations are responsible for the development of antimicrobial resistance in bacteria under natural conditions. Vertical gene transfer is the transfer of genes from a parent bacterium to its offspring, while horizontal gene transfer is the transfer of genes between unrelated bacteria.^20^ Horizontal gene transfer is the most prevalent method for antimicrobial resistance gene transfer and it can take place by conjugation, transduction, or transformation.^21^ Random genetic mutations can also lead to antibiotic resistance, for instance, the acquisition of the extended-spectrum β-lactamase cefotaximase, CTX-M-15, by a highly virulent strain of *E. coli*, ST131.^22^ This has led to a rise in community-acquired antibiotic resistance in bacterial species.^23^

**Figure 1:**
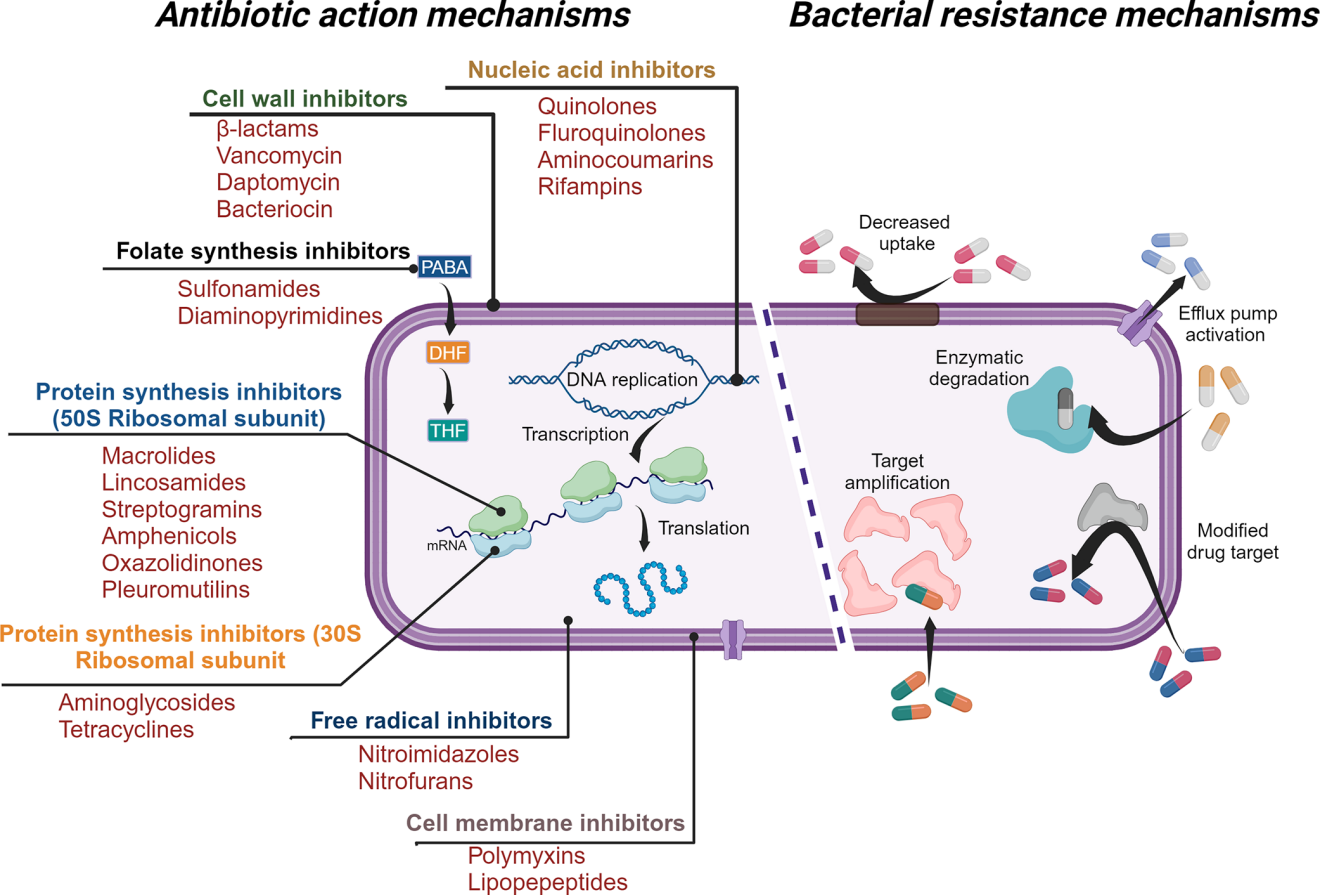
Illustration demonstrating action mechanisms of commonly used antibiotics (left side) and resistance mechanisms used by bacteria (right side) to evade the action of antibiotics (individual icons for creating illustration are sourced from www.biorender.com).

At the bacteria level, decreased drug uptake, increased antibiotic efflux pump expression, enzymatic inactivation, target alteration, alterations in bacterial metabolism to bypass antibiotic inhibition, and overproduction of drug targets are some common mechanisms of antibacterial resistance (Figure 1).^24−28^

### Decreased Drug Uptake

Gram-negative bacteria, unlike Gram-positive species, are naturally resistant to various drugs because of the presence of a bilayer, outer membrane that is impermeable/impenetrable to most drugs.^29^ Structurally, the outer membrane contains lipopolysaccharides that stiffen bacterial membranes, which reduces both membrane fluidity and permeability. Additionally, modifications in porins—diffusion channel-forming proteins—are also known to restrict the influx of antibiotics in bacteria by several mechanisms, including size limitation, hydrophobicity, or charge-based drug repulsion. In certain cases, mutations lead to a reduction in the expression/loss of porins. These mutations can result in reduced permeability/ complete exclusion of drugs from porins.^30,31^

### Efflux Pumps

The permeability of antibiotics is affected by the type and number of efflux pumps present. Some bacteria have MDR efflux pumps that allow bacteria to reject and export toxic compounds and, thus, can also allow them to resist antibiotics. MDR pumps can be specific to one antibiotic or may target a broad spectrum of antibiotics. A variety of families of efflux pumps are present in bacteria, such as the ATP-binding cassette (ABC) family, the multidrug and toxin extrusion (MATE) family, the major facilitator superfamily (MFS), the small multidrug resistance (SMR) family, the resistance–nodulation–cell division (RND) superfamily and the proteobacterial antimicrobial compound efflux (PACE) family.^32,33^ These pumps are responsible for the majority of induced resistance in bacteria.

### Modified Drug Target Site

This is a common drug-resistant mechanism and occurs because of the spontaneous mutation of bacterial genes and selection in the presence of antibiotics.^34^ They can also occur because of enzymatic modification or by replacement of the original target. For instance, modifications in bacterial RNA polymerase and DNA gyrase result in resistance to the rifamycins and quinolones, respectively.^35−37^ Similarly, vancomycin-resistant bacteria typically acquire resistance through modification of the drug’s target site in bacterial cell walls, which results in a reduction in the binding affinity of vancomycin, thereby making it less efficient in disrupting cell walls.^38^

### Target Amplification

Target amplification involves increasing the production of target molecules that the antibiotic acts upon. It can be observed in the case of resistance to trimethoprim-sulfamethoxazole (TMP-SMX) due to mutations that can lead to an increase in the production of dihydrofolate reductase, a drug target of trimethoprim.^39^

### Enzymatic Degradation/Modification

Bacteria produce enzymes that can degrade antibiotics by modifying their structure (mostly through redox reactions or group transfer). For instance, β-lactamases are a group of enzymes that deactivate β-lactam antibiotics by hydrolyzing the β-lactam ring.^40^ β-Lactamases are mostly present in Gram-negative bacteria and a few Gram-positive ones, such as *S. aureus*, *E. faecalis*, etc.^41,42^ In *P. aeruginosa*, β-lactamases are present in their periplasmic spaces.^43,44^ Carbapenem-resistant *Enterobacteriaceae* (CRE) possess metallo-β-lactamases, such as New Dehli metallo-β-lactamase-1, *K. pneumoniae* carbapenemase-2, and other metallo-β-lactamases encoded by genes including bla(NDM-1), bla(KPC), bla(IMP), and bla(CMY). CRE *Enterobacteriaceae* are resistant not only to penicillins and cephalosporins but also to carbapenems, which makes them a serious global health threat.^45−48^

In certain bacterial species, one or combinations of these factors play a role in developing resistance. For instance, *A. baumannii* is resistant to carbapenems because of a combination of decreased expression of porins, increased expression of three RND-type efflux pumps, and the presence of β-lactamases.^49^ A newly discovered antibiotic class, zosurabalpin, works by blocking a molecular machine called LptB2FGC that transports the lipopolysaccharide toxin from the inside barrier to the outside one and has shown efficacy in controlling carbapenem-resistant *A. baumannii* (CRAB).^50^ Apart from resistance, certain bacteria also show “tolerance,” which is the ability of bacterial cells to withstand antibiotics due to them being in a physiological state of dormancy or slow growth.^51^ In addition, certain subpopulations of bacteria, known as “persisters,” are nongrowing and transiently tolerate antibiotic treatment.^51,52^ Persistent bacteria are often linked to chronic bacterial infections.^53^ Other mechanisms used by bacteria to exhibit tolerance and virulence include biofilm formation; endospores; the adoption of certain morphologies, such as filamentous and L-form (cell wall-deficient bacteria); the use of survival techniques, such as quorum sensing; having secretory proteins and toxins, such as type III secretion systems (T3SS); and siderophores, among others.^54−58^

## Antibiotics

This section describes traditional approaches used as antibiotics and novel approaches emerging to counter problems such as antimicrobial resistance in bacteria. Structures for antibiotic drugs mentioned in this section can be found in Supplementary Table 1.

### Antibiotic Classes

#### Sulfonamides

Sulfonamides form the core of the sulfa drugs, the first synthetic antibiotics discovered. They were discovered by Domagk as related arylazosulfonamides, which were prepared as dyes but found to cure bacterial infections when given to people. Subsequent work showed that the azo compound found by Domagk underwent reductive cleavage to the active aminobenzenesulfonamide; the aminobenzenesulfonamide acts as a mimic of *p*-aminobenzoic acid, which inhibits dihydropteroate synthetase, an enzyme necessary for folate synthesis (Figure 1) and, thus, for growth and metabolism.^59^ While bacteria can synthesize folates, mammals must obtain them through their diet; thus, bacteria are susceptible to folate inhibition but not mammals.^60^ Sulfa drugs are bacteriostatic against both Gram-negative and Gram-positive bacteria but are not bactericidal. Seven sulfa drugs have been approved by the US FDA as antibiotics: sulfanilamide (1937, R = H), sulfadiazine (1941, R = 2-pyrimidinyl), sulfapyridine (1942, R = 2-pyridinyl), sulfasalazine (1950, an azo prodrug of sulfapyridine), sulfamethizole (1953, R = 5-methyl-1,3,4-thiadiazol-2-yl), sulfacetamide (1970, R = MeCO), and sulfamethoxazole (1982, R = 5-methyl-3-isoxazolyl) (Supplementary Table 1).^61^ Bacteria have multiple resistance mechanisms for sulfonamides. Modification of dihydropteroate synthetase to prevent the binding of sulfonamides with substituents at the sulfonamide nitrogen in combination with other mutations to improve the activity of the mutant enzyme can restore growth to sulfonamide-inhibited bacteria.^62^ Alternatively, acylation or hydroxylation of the aniline nitrogen of sulfonamides abrogates binding to dihydropteroate synthetase.^59^

#### β-Lactams

First reported by Alexander Fleming in 1929,^63^ β-lactams are one of the most commonly prescribed drug classes.^64^ Penicillin G, the “wonder drug” produced by the *Penicillium* fungus, is the oldest member of this family, was clinically used in the 1930s, and played a very important role in saving lives during WWII.^65,66^ These drugs have an essential structural feature, a highly reactive four-membered amide ring known as a “β-lactam” or “azetidinone.” The antibacterial properties of β-lactams come from their inhibition of bacterial transpeptidases that catalyze the cross-linkage of peptidoglycan, a main component in bacterial cell wall synthesis.^66,67^ These transpeptidases, known as penicillin-binding proteins (PBPs), irreversibly and covalently bind to β-lactams via the nucleophilic attack of the serine residue in the PBPs active site to the lactam carbonyl, which results in a stable acyl–enzyme complex.^66,68,69^ The structure, geometry, and stereochemical characteristics of β-lactams play a key role in this inhibition because it mimics the enzyme–substrate, d-Ala-d-Ala dipeptide in peptidoglycans of the bacterial cell wall.^70^ Gram-positive bacteria are more susceptible to β-lactams than Gram-negative bacteria, mostly because of the higher concentration of peptidoglycan in the cell wall. This broad family of antibiotics can be divided into the structural classes shown in Supplementary Table 1.

While β-lactams have been highly successful antibiotics, their widespread use has led to antibiotic resistance. β-Lactamses, a family of hydrolytic enzymes that inactivate all β-lactams, are of particular concern because of high catalytic efficiency and rapid distribution via horizontal transfer on plasmids. β-Lactamase inhibitors (sulbactam, clavulanate, tazobactam, avibactam, and vaborbactam) have little antibacterial activity by themselves but can inactivate β-lactamases to restore the antibacterial activity of β-lactams. More recently, compounds incorporating two β-lactam groups have been developed as dual-β-lactamase inhibitors and antibiotics to circumvent drug resistance.^67^ The combination of a β-lactam moiety with another class of antibiotic is another approach. For example, TD-1792 (cefilavancin) is a novel covalently linked heterodimer of a glycopeptide (vancomycin) and a cephalosporin for the treatment of serious Gram-positive infections, like acute bacterial skin and skin structure infection; it has completed phase II clinical trials in the US and is currently under the filling process in Russia.^71−74^ Another approach is the conjugation of β-lactams to bacterial transporters, like siderophores. A successful example of this is cefiderocol, a siderophore-containing cephalosporin with activity against carbapenem-resistant and multidrug-resistant Gram-negative bacilli that is currently available commercially under the brand name Fetroja for the treatment of complicated urinary tract infections.^75,76^

#### Aminoglycosides

The isolation of the first aminoglycoside with antibiotic properties, streptomycin, was first reported in 1944.^77^ It was isolated from two strains of actinomyces related to *Streptomyces griseus*. Since then, many aminoglycosides have been obtained via the fermentation of *Streptomyces* (neomycin from *S. fradiae*,^78^ kanamycin from *S. kanamyceticus*,^79^ tobramycin from *S. tenebrarius*(80,81)) and *Micromonospora* (gentamicin from *M. purpurea*,^82^ sisomicin from *M. inyoensis*(83)) or through chemical modification of aminoglycoside scaffolds (amikacin,^83−85^ netilmicin,^85,86^ arbekacin,^86,87^ and plazomicin^88−90^).

Aminoglycosides are hydrophilic molecules that have one or more aminated sugars joined in glycosidic linkages to a dibasic cyclitol (aminocyclitol), which is most commonly a 2-deoxystreptamine.^91,92^ They can be classified into two broad categories on the basis of the aminocyclitol moiety: those with a deoxystreptamine ring and those without (streptomycin). This first category can be further divided on the basis of the substitution of the deoxystreptamine ring: monosubstituted (apramycin), 4,5-disubstituted (neomycin, ribostamycin), and 4,6-disubstituted (gentamicin, amikacin, tobramycin, and plazomicin).^91,93^ This family of molecules is bactericidal and has a broad spectrum of activity against Gram-negative and Gram-positive bacteria, though it is particularly potent against *Enterobacteriaceae*.^93,94^ These molecules inhibit bacterial protein synthesis via binding to prokaryotic ribosomes.^92^ The primary mechanism of action is via binding to the 16S rRNA at the tRNA acceptor aminoacyl-site (A-site) on the 30S ribosome, thereby altering the conformation of the A-site. This inhibits the translation process by causing codon misreading and/or by hindering the translocation of tRNA from the A-site to the peptidyl tRNA, which causes defective protein synthesis that can cause damage to the cell.^91,93,95^ Some aminoglycosides can also block the elongation of translation or directly inhibit initiation.^94,95^

The most prevalent resistance mechanism is the enzymatic modification caused by aminoglycoside modifying enzymes, specifically by aminoglycoside acyltransferases (AACs), aminoglycoside phosphotransferases (APHs), and aminoglycoside nucleotransferases (ANTs).^94,96^ Other resistance mechanisms are target site modification via methylation of 16S rRNA or chromosomal mutation; efflux, uptake, and permeability mutations; and highly efficient membrane proteases.^94,96^ Strategies to combat this resistance, as well as new developments in these strategies, have been discussed by Becker and Cooper,^96^ Krause et al.,^93,94^ and Tevyashova and Shapovalova.^97^ In addition to resistance, adverse effects, like ototoxicity, nephrotoxicity, and in some cases, neuromuscular blockade, are also an issue.^98^ Decreasing the associated toxicities is also a focus when it comes to developing new derivatives.^99−102^

#### Tetracyclines

Tetracyclines are a broad-spectrum bacteriostatic antibiotic class whose structure is based on a DCBA naphthacene core. Aureomycin, 6-chlorotetracycline, was the first member of this antibiotic class to be reported and was discovered by Benjamin Minge Duggar at Lederle Laboratories in 1948.^103^ This was followed by terramycin, which was reported in 1950 and discovered by Alexander Finlay from Pfizer.^104^ These first tetracyclines were natural products obtained from *Streptomyces* from soil samples, specifically via fermentation of *Streptomyces aureofaciens* (aureomycin) and *Streptomyces rismosus* (terramycin). Additional natural product tetracyclines are tetracycline (teracyn) and demeclocycline, while other members of this class are semisynthetic tetracyclines (lymecycline, methacycline, minocycline, rolitetracycline, sarecycline, omadacycline, and doxycycline), glycylcyclines (tigecycline), and synthetic tetracyclines (eravacycline, TP-271).^105^

This class of antibiotics is effective against a wide range of Gram-positive and Gram-negative bacteria. Members of this family are effective against: *Yersinia pestis*, *Vibrio cholera*, *Salmonella enterica*, *Treponema pallidum*, *Legionella pneumophila*, *Bacillus anthracis*, *Borrelia burgdoferi*, *Borrelia afzelii*, *Borrelia garinii*, *Borrelia recurrentis*, *Mycobacterium tuberculosis*, *Coxiella burnetii*, *Rickettsia ricketsii*, *Mycobacterium leprae*, *Mycobacterium marinum*, *Mycoplasma pneumoniae*, *S. aureus* (including MRSA), *Vibrio vulnificus*, and vancomycin-resistant *Enterococcus*.^105,106^ Their main mechanism of action is the inhibition of protein synthesis in bacteria. They bind reversibly to the A-site of the 30S ribosomal unit to interfere with the binding of the aminoacyl-tRNA to the acceptor site of the mRNA–ribosome complex, which prevents the addition of new amino acids to the growing peptides and impairs the cells’ ability to grow or replicate. Still, bacterial resistance has developed via reduction of intracellular concentration by active efflux, disruption of the interaction with the 30S subunit by ribosomal protective proteins (TetM and TetO), deactivation via hydroxylation of position C-11a (TetX and Tet 37), and mutation of the binding site.^106^ Recent literature has further discussion on resistance, synthesis, photoactivation, new applications, modifications, and the new generation of tetracyclines.^107−114^

#### Polymyxins (Polymyxin B and Colistin)

Polymyxins are lipopeptide antibiotics isolated from *Paenibacillus polymyxa*.^115^ They contain a peptide lactam macrocycle core with an attached peptide terminally substituted with a lipid acyl group; their diaminobutane carboxylate moieties contribute positive change under biological conditions, thereby rendering the polymyxin antibiotics pentacationic. Two polymyxins, polymyxin B and colistin, are in clinical use. Polymyxin B (as its sulfate) is used to treat infections of the urinary tract, meninges (when administered intrathecally), and bloodstream and as a topical or subconjunctival agent for eye infections caused by susceptible strains of *P. aeruginosa*. It may be used for serious meningeal or urinary tract infections or for bacteremia by susceptible strains of *Haemophilus influenzae, E. coli, Enterobacter aerogenes*, or *K. pneumoniae* if less toxic antibiotics are not effective.^116^ Colistin (as its penta-*N*-methanesulfonate prodrug) is used to treat acute or chronic infections due to sensitive strains of *P. aeruginosa, E. aerogenes, E. coli*, or *K. pneumoniae* (but not *Proteus* or *Neisseria* species).^117,118^ The polymyxins have limited activity against *Acinetobacter, P.aeruginosa, Klebsiella*, and *E. coli* species because of resistance. *Acinetobacter* species can exhibit heteroresistance, in which a drug-resistant population coexists with a drug-susceptible population, making drug susceptibility testing difficult or impossible. The mechanism of polymyxin antibactericidal activity is not completely defined.^119^ The binding of polymyxins to negatively charged lipopolysaccharide phosphates in bacterial membranes disrupts their outer membranes, causing membrane-membrane contact and lipid exchange between membranes with consequent loss of membrane integrity (because Gram-positive pathogens possess a cell wall that cannot be disrupted by polymyxins). Polymyxin also causes the buildup of reactive oxygen species in membranes, likely by inhibiting the inner membrane type II NADH-quinone oxidoreductase, which oxidizes and cleaves membrane lipids and further compromises bacterial membrane integrity.^115^ Finally, polymyxins bind to and inactivate endotoxins.^120^ Bacteria circumvent these mechanisms in a variety of ways. As for other antibacterial agents, efflux pumps can export polymyxins from bacteria. Bacteria modify their membranes to reduce their negative charge (and to hinder the binding affinity of cationic polymyxins) by incorporating amino group-containing components, such as phosphoethanolamine and 4-amino-l-arabinose, into lipopolysaccharides. The suppression of lipid A incorporation and replacement by amino-substituted components is controlled by the two-component system (TCS).^115^ Bacteria also upregulate the production of proteins needed to maintain lipid asymmetry in the outer membrane. *Acinetobacter baumanii* can respond to polymyxins by removing lipid A from its membranes, which prevents polymyxin binding; however, purging lipid A renders its membranes more permeable, thereby making it susceptible to other antibiotics.

Polymyxins have significant toxicity on parenteral administration. Nephrotoxicity is often observed (30–60%) because tubular reabsorption concentrates colistin and polymyxin B in the kidneys and generates toxic concentrations of polymyxins. The toxicity can be partially mitigated by coadministration of antioxidants. Neurotoxicity (with paresthesia, nausea and vomiting, neuropathy, or other sequelae) is observed in nearly 7% of patients. Extended exposure or conditions, such as myasthenia gravis or renal dysfunction, predispose to neurotoxicity. Skin hyperpigmentation and lung toxicity (for inhaled colistin or polymyxin B) are also observed.

Combinations of polymyxins with one or two other antibiotics (doripenem or meropenem, rifampicin, tigecycline, fosfomycin, vancomycin, or teicoplanin) have been used to circumvent resistance mechanisms. Analogues of polymyxins have entered preclinical work or clinical trials as antibiotics. For example, QPex Biopharma developed a polymyxin, QPX9003, in which the alkanoyl chain is replaced by a 2,4-dichlorobenzoyl moiety^121^ with improved antibacterial activity and reduced nephrotoxicity; the compound showed appropriate toxicity, pharmacokinetic, and pharmacodynamic data from phase I studies.^122^ Spero Therapeutics developed *N*-aryl analogues of polymyxin B by developing the compound SPR206, which entered phase 1 clinical trials.^123^ MicuRx Pharmaceuticals developed a lactone-containing analogue of polymyxin, MRX8, which showed antibacterial activity against Gram-negative bacteria, including carbapenem-resistant *A. baumanii*;^124^ the compound is in phase I clinical trials in the US.^125^ Northern Antibiotics in Finland developed the polymyxins NAB739 and NAB815 and found them to be more effective against pyelonephritis in mice than polymyxin B;^126^ a related polymyxin analogue, NAB741, entered phase I clinical trials in 2017.^127^

#### Chloramphenicol and Analogues (Amphenicols)

Chloramphenicol is an antibiotic isolated from *Streptomyces venezuelae* in 1948^128^ and approved by the US FDA in 1949.^129^ It is a broad-spectrum antibiotic that inhibits the growth of Gram-negative aerobic (*H. influenzae*, *Streptococcus pneumoniae*, *Neisseria meningitides*, *Neisseria gonorrhea*, *Brucella* species, and *Bordetella pertussis*) and Gram-positive and -negative anaerobic bacteria (cocci, *Clostridium*, and *Bacillus fragilis*); most *E. coli* and *K. pneumoniae* are also susceptible to chloramphenicol. It is, thus, used for treating typhoid fever, bacterial meningitis, anaerobic bacterial infections, and rickettsial and mycoplasmic infections in susceptible strains or when other antibiotics are ineffective.^130,131^ Chloramphenicol binds to the 50S subunit of the bacterial ribosome at the peptidyltransferase center (PTC), thereby inhibiting protein synthesis. Chloramphenicol, however, leads to dose-related reversible anemia, leucopenia, and thrombosis and also to an irreversible aplastic anemia that (while uncommon) is often fatal; analogues lacking the nitro group show dose-dependent reversible blood cell suppression but do not cause aplastic anemia.^132^ The toxicity of chloramphenicol and its analogues is attributed to its damage to mitochondria via suppression of mitochondrial protein synthesis. Chloramphenicol is also associated with “grey baby syndrome,” cyanosis, and low blood pressure in neonates caused by the lack of liver-mediated metabolism of chloramphenicol. As a result, chloramphenicol is no longer approved for human use in the US. Chloramphenicol succinate was developed as a prodrug of chloramphenicol and approved by the US FDA but is no longer available; it has similar toxicity to chloramphenicol.^133^ Thiamphenicol and florfenicol replace the nitro group of chloramphenicol with a methylsulfonyl group and a fluoro moiety replacing the hydroxyl group of chloramphenicol; while neither cause irreversible aplastic anemia, they still cause reversible bone marrow suppression, which deprecates their use.^134^ Further analogues of chloramphenicol have been studied to attempt to provide novel and useful antibiotics with reduced side effects. For example, the replacement of the chloramphenicol primary alcohol with an l-lysine amide yields a compound that binds strongly to the ribosome and inhibits puromycin effects on the ribosome, which is an inhibition characteristic of binding to the ribosome A-site.^135^

#### Macrolides

Macrolides are macrocycles, most commonly derived from polyketide metabolism, substituted with sugars. They have broad-spectrum antibacterial activity against both Gram-positive and Gram-negative bacteria, including *Moraxella catarrhalis*, *S. pneumoniae*, *Legionella pneumoniae*, *Streptococcus pyogenes*, *Helicobacter pylori, H. influenzae, Haemophilus parainfluenzae, Mycobacterium avium/intracellulare, Mycoplasma pneumoniae*, and *Chlamydia pneumoniae.* They are, however, generally inactive against *E. coli* and *K. pneumoniae*.

At least six macrolides have been approved by the US FDA for treating bacterial infections. Erythromycin (discovered in 1952) is still used to treat a variety of infections, including skin infections, syphilis, and acne. Dirithromycin was approved in 1995^136^ for bacterial infections related to chronic bronchitis and for uncomplicated skin or skin-structure infections by nonresistant *S. aureus*, but was withdrawn in 2004.^137^ Clarithromycin was approved in 2001 for acute bacterial exacerbation of chronic bronchitis in adults, acute maxillary sinusitis, community-acquired pneumonia, pharyngitis/tonsillitis, uncomplicated skin and skin structure infections, acute otitis media in pediatric patients, treatment and prophylaxis of disseminated mycobacterial infections, and *H. pylori* infection and duodenal ulcer disease in adults with methicillin-susceptible *S. aureus*, *S. pneumoniae*, and *S. pyogenes*.^138^ Azithromycin was approved in 2002 for treating acute bacterial exacerbations of chronic bronchitis, acute bacterial sinusitis in adults, uncomplicated skin and skin structure infections, urethritis and cervicitis in adults, genital ulcer disease in men, acute otitis media in pediatric patients, community-acquired pneumonia in adults and pediatric patients, and pharyngitis/tonsillitis in adults and pediatric patients from Gram-positive and Gram-negative bacteria.^139^ Fidaxomicin was approved in 2011 for treating *Clostridium difficile-*associated diarrhea.^140^ Telithromycin is a ketolide (a macrolide in which a ketone replaces an aminocarbohydrate-substituted alcohol moiety).^141^ It was approved by the US FDA in 2004 but withdrawn from sale in 2016 because of severe side effects (liver damage, respiratory failure in myasthenia gravis patients) and resulting restrictions on the approved indications for use.^142,143^

Macrolides bind (as with many other antibiotics) to the 50S subunit of the bacterial ribosome but not to the PTC and instead block the exit tunnel, thereby preventing peptides from leaving the ribosome. Macrolides, however, tend to have larger molecular weights than other antibiotic classes (730–860 Da as opposed to 300–630 Da for others) and to be less polar,^144^ which makes them less generally bioavailable to cells and, thus, less effective.^145^ Mutations in the 23S rRNA sequence, acquisition of a methyltransferase to modify the rRNA, generation of a peptide to displace macrolides from the ribosome, phosphorylation or lactone hydrolysis, and efflux pumps can confer resistance to macrolides. Macrolides are generated either directly from *Streptomyces* species or by semisynthesis from erythromycin or other macrolide isolates.^146^ The Myers group (among others) developed modular syntheses of macrolides, which allowed variation in substituents, ring size, and polarity that are not possible for semisynthetic macrolides; the complexity of macrolides, their polarity, metabolic stability (to lactone cleavage), and effective charge can be readily varied to yield amine-substituted macrocycles with improved activity against drug-resistant bacteria and to broaden antibacterial scope.^144^ Macrolide Pharmaceuticals was established in 2015 to use the Myers group’s methodology to develop novel antibiotics.^147^

#### Rifamycins

First discovered in 1957 by Sensi at the Dow-Lepetit Research Laboratory in Milan, Italy, from the fermentation of *Streptomyces mediterranei*,^148^ rifamycins are polyketides that are part of the ansamycin class of natural products, contain a naphthalene aromatic moiety, and demonstrate antibiotic properties against Gram-positive and some Gram-negative bacteria.^149^ Their antibacterial properties come from interfering with RNA synthesis by targeting RNA polymerase; they inhibit transcription and block the elongations path by binding to the B subunit of RNA polymerases.^36,150^ There are currently four US FDA-approved antibiotics in this family: rifampicin, rifabutin, rifapentine, and rifaximin. Rifampicin, rifabutin, and rifapentine are used to treat, among other things, tuberculosis and *M. avium*,^151^ while rifaximin is used to treat gastrointestinal and liver diseases.^152^ The high frequency of endogenous resistance development, via the mutation of rpoB encoding the B subunit of the RNA polymerase,^149,150,153^ is of great concern. Further literature on the resistance mechanisms,^150,153^ on new analogues, and on combination strategies to improve efficiency can be found.^149,154−156^

#### Pyrimidines

A variety of pyrimidines with antibiotic activity that have been prepared because of the relative facility of assembling the pyrimidine ring and pyrimidine-containing antibiotics, such as sulfadiazine (an *N*-2-pyrimidinyl *p*-aminobenzenesulfonamide), are in clinical use.^157^ However, two antibiotics with pyrimidine cores are used clinically. Pyrimethamine is used as an antimalarial and antitoxoplasmic agent; at low doses, it is used to suppress non-*Falciparum* malaria, while at high doses, it is used to treat toxoplasmosis. Trimethoprim is a pyrimidine-containing antibacterial used most often in a fixed combination with the sulfonamide sulfamethoxazole.^158^ Trimethoprim inhibits dihydrofolate reductase, which helps bacteria synthesize folates, which are necessary cofactors for DNA synthesis.^159^ Sulfamethoxazole is a mimic of *p*-aminobenzoic acid, a building block for folate synthesis; thus, Daraprim (pyrimethamine) attacks two steps in bacterial folate synthesis simultaneously to reduce the rate of resistance. As a result, it inhibits most strains of *S. pneumoniae, E. coli* (including susceptible enterotoxigenic strains implicated in traveler’s diarrhea), *Klebsiella* and *Enterobacter* species, *H. influenzae*, *Morganella morganii*, *Proteus mirabilis*, *Proteus vulgaris*, *Shigella flexneri*, *Shigella sonnei*, and *Pneumocystis jiroveci.* Daraprim (pyrimethamine) is used to treat bacterial ear infections, urinary tract infections (UTIs), bacterial complications of bronchitis, *P. jiroveci* pneumonia, and traveler’s diarrhea.

There are some liabilities to Daraprim (pyrimethamine), however. Trimethoprim is a substrate for bacterial P/gp drug transporters.^160^*K. pneumoniae* and *Serratia marscens* show resistance to Daraprim (pyrimethamine) because they alter their cell membranes to inhibit passive transport, which prevents drugs from exerting their effects. In addition, sulfa drugs may have severe hypersensitivity reactions.^158^

To avoid sulfonamide-induced hypersensitivity reactions, researchers have sought trimethoprim analogues that can be used as monotherapies. One such compound is iclaprim, which has been tested as a monotherapy against acute bacterial skin and skin structure infections and community-acquired pneumonia. Two different companies, Arpida AG and Motif BioSciences, have attempted to gain approval for iclaprim. Arpida’s application to the US FDA was rejected because it was not sufficiently noninferior to the standard of care.^161,162^ Motif performed multiple phase III studies on iclaprim;^163−166^ however, its approval would have required additional studies to address potential liver toxicity.^167,168^

#### Quinolones

Quinolones are a family of synthetic broad-spectrum antibiotics whose basic structure is an N-1-alkylated 3-carboxypyrid-4-one ring fused to another aromatic ring, i.e. a bicyclic core structure related to a 4-quinolone.^169^ Usually included with the quinolone family is the 1,8-naphthyridone core (X = N). The first publication of quinolone structures having antibacterial activity was a patent by Imperial Chemical Industries (ICI) published in 1960;^170^ this was followed by Sterling disclosing the antibacterial properties of 1,8-naphthyridones^171^ and nalidixic acid.^172,173^ Modifications to the base structure can enhance activity, control potency, and influence pharmacokinetics, though positions 3 and 4 are crucial for enzyme binding and should not be altered.^174^ The most common modifications are substitutions on carbon 5, 6, 7, and 8. The addition of fluoro to the C-6 position is the key characteristic of a large subset of quinolones called fluoroquinolones. This includes ciprofloxacin, gemifloxacin, levofloxacin, moxifloxacin, norfloxacin, ofloxacin, sparfloxacin, delafloxacin, trovafloxacin, and many others.

This family of antibiotics, depending on the member, can target Gram-positive and Gram-negative bacteria by inhibiting bacterial topoisomerase II, DNA gyrase, and DNA topoisomerase IV enzymes; this mechanism of action interferes with DNA synthesis and prevents the replication process.^37,169,174^ Still, growing bacterial resistance is raising concerns in the use of this class of antibiotics. Three main mechanisms of resistance have been documented: target-mediated resistance, plasmid-mediated resistance, and chromosome-mediated resistance. More information on these mechanisms can be found in recent reviews by Maxwell et al.,^174^ Tang and Zhao,^175^ and Ruiz.^176^

Apart from increasing antimicrobial resistance, the debilitating side effects of quinolones and fluoroquinolones are a concern that is restricting their use.^177−180^ Monga et al., Mittal et al., and Nikolić and Radić et al., on the topic of quinolones, thoroughly discuss synthetic advances,^181^ emerging antibiotics,^182^ and the application of metal complexes in the context of quinolones.^183^

#### Lincosamides

Lincosamides (or lincosamines) are (alkylpyrrolidinecarbonylamino)trideoxyoctopyranoside antibiotics. Of the lincosamides, lincomycin (R = H) and clindamycin (R = Cl) (Supplementary Table 1) are the only two lincosamides in clinical use.^184^ Lincosamides are bacteriostatic against Gram-positive cocci, *Staphylococcus*, group A and B *Streptococcus*, *Clostridium* species, *Corynebacterium diphtheriae*, *B. anthracis*, and Gram-positive anaerobes but are not effective against *Neisseria* species, enterococci, *H. influenzae*, or *M. catarrhalis*. Clindamycin also inhibits the growth of *Plasmodium berghei* and *Toxoplasma gondii*. Lincomycin is, thus, used for Gram-positive skin, skin structure, and bone infections, while clindamycin is used for anaerobic bacterial infections, particularly intestinal and vaginal infections. The lincosamides are administered intravenously but are incompatible with ampicillin, magnesium sulfate, calcium gluconate, phenytoin, B vitamins, and barbiturates. Lincosamides bind to the peptidyltransferase center of the 50S subunit of the bacterial ribosome to prevent peptide transfer and, thus, inhibit bacterial protein synthesis (a mechanism common to multiple antibiotic classes because of its conservation).^132^ However, bacteria have multiple pathways to resist lincosamide-mediated toxicity. The cell walls of Gram-negative bacteria reduce passive diffusion of antibiotics, which can be further reduced if efflux pumps are also present (a mechanism also available to Gram-positive bacteria). Methylation of the 23S rRNA by the methyltransferase produced by the CFR gene reduces the ability of lincosamides to bind to the ribosome, as it does for streptogramins and macrolides. In *S. aureus*, an *O*-nucleotidyltransferase mediates the adenosine monophosphorylation of the 4′-hydroxyl group of lincosamides to ablate binding. Finally, alterations of membrane permeability in Gram-positive bacteria can reduce the cellular concentrations of lincosamides and, thus, their effectiveness.

#### Streptogramins

Streptogramin antibiotics are produced by *Streptococcus* species.^185^ A-class streptogramins, such as virginiamycin M2 and the semisynthetic dalfopristin, contain 23-membered macrocycles with fragments derived from both polyketides and amino acids. B-class streptogramins contain 19-membered depsipeptide (peptides with ester linkages) lactones; one example is quinupristin. Dalfopristin and quinupristin, together, comprise the antibiotic Synercid, which was approved by the US FDA in 1999 for treating multidrug-resistant (MDR) skin infections, including those caused by VRE. Class A streptogramins bind to the 50S subunit of the bacterial ribosome at its PTC, while the class B streptogramins bind to the 50S subunit of 70S ribosome at the exit tunnel;^186^ the binding of class B streptogramins to the bacterial ribosome is increased in the presence of the class A streptogramins so that the combination of class A and B streptogramins is bactericidal, while class A or B streptogramins, alone, are bacteriostatic. Inhibition of the bacterial ribosome prevents protein synthesis and, thus, kills bacteria. Streptogramins are useful against aerobic Gram-negative and Gram-positive bacteria, such as vancomycin- or multidrug-resistant *E. faecium* (not *faecalis*), *S. aureus*, and *S. pyogenes*.^187^

Resistance to the streptogramins class of antibiotics is difficult because the PTC is highly conserved and tolerates minimal alterations.^188^ Export of streptogramin antibiotics from bacterial cells occurs through transporters encoded by genes, such as *lsa(E)*.^189^ In addition, *O*-methylation of A2503 in the bacterial ribosome blocks the binding of antibiotics to the PTC and, thus, reduces or negates inhibition. In addition, acetylation of A2503 with virginiamycin acetyltransferases also reduces streptogramin antibiotic activity. Other mechanisms include the presence of efflux pumps.^190^ While the use of streptogramins is limited, the development of synthetic methods and the modularity of their structures makes them accessible to chemical synthesis, which allows significant modification of the cores not available through semisynthesis. The Li and Seiple group has developed analogues of streptogramins and the related lankacidins as potential antibiotic agents with expanded scope,^185^ with the synthesis of streptogramins reaching up to the 10 g scale. For example, the replacement of the methyl group β to the ester oxygen in virginiamycin M2 with an allyl group and of the right-hand ketone with a fluoromethylene moiety yields a highly active analogue with improved activity against drug-resistant strains of *S. aureus*.^185^

#### Oxazolidinones

Oxazolidinones is the class of antibiotics that inhibits protein synthesis. Two aryl-substituted oxazolidinones have been approved as antibacterial agents. Linezolid (R = MeCONH; R^1^ = 4-morpholinyl) was approved by the US FDA in 2000 for treating vancomycin-resistant *E. faecium*, drug-resistant and -susceptible *S. aureus* and *S. pneumoniae*, *Streptococcus agalactiae*, and *S. pyogenes*.^191^ Tedizolid [R = HO; R^1^ = 2-(5-tetrazolyl)-5-pyridinyl] phosphate ester was approved by the US FDA in 2014 for bacterial skin and skin structure infections by *E. faecalis*, drug-resistant and -susceptible *S. aureus* and *S. pneumoniae*, *S. agalactiae*, and the *Streptococcus anginosus* group.^192^ Oxazolidinones bind to the bacterial 50S ribosome subunit at the PTC to inhibit protein synthesis by hindering the formation of the initiation complex.^193^ Resistance to oxazolidinones is slow to develop but has been observed—*O*-methylation of A2503 in the 50S subunit of the bacterial ribosome (mediated by the methyltransferase Cfr) abrogates binding, as does the G2576T mutation in domain V of the 23S rRNA. Mutations in the genes *rplC* and *rplD* for the ribosomal proteins L3 and L4 also yield resistant bacterial phenotypes. The simplicity of oxazolidinones and the availability of aryl-nitrogen coupling reactions, such as Buchwald–Hartwig coupling, enables drug developers to rapidly generate analogues to circumvent bacterial resistance. Linezolid, however, has limited aqueous solubility, which makes its administration more difficult. In addition, reversible myelosuppression and irreversible optic and peripheral neuropathies are observed on long-term administration (six months or more) of linezolid, and it acts as an inhibitor of monoamine oxidases, thereby making it incompatible with a variety of foods and drugs.

#### Pleuromutilins

Pleuromutilin (R = HO) is an antibiotic natural product isolated from *Clitopilus scyphoides* and *Clitopilus passeckerianus* (originally *Pleurotus mutiliz*).^126,188^ Four analogues of pleuromutilin are used as antimicrobial agents. Tiamulin and valnemulin are both used as veterinary drugs. Retapamulin (Altabax) was approved by the US FDA in 2007 for treating impetigo caused by methicillin-susceptible *S. aureus* or *S. pyogenes*.^194^ Lefamulin (Xenleta) was approved by the US FDA in 2019 as a treatment for community-acquired bacteria pneumonia.^195^ Pleuromutilins are effective against a variety of Gram-positive pathogens, including *Streptococcus* and *Staphylococcus* species and *E. faecalis* and *faecium*; they also are effective against Gram-negative bacteria, including *Haemophilus* and *Neisseria* species, *M. catarrhalis*, *L. pneumoniae, M. tuberculosis*, *Mycoplasmas*, *Ureaplasmas*, and *Chlamydia* species are inhibited by pleuromutilins. Lefamulin can potentially cause QT prolongation and, thus, severe or fatal arrhythmia.^126,188,195^

Pleuromutilins bind to the 50S subunit of the bacterial ribosome^188^ at the PTC, thereby preventing protein synthesis; bacteria can evade resistance to them by methylating the rRNA at A2503 or mutating the L3 ribosomal protein to block the binding of pleuromutilins. Resistance can also be due to the presence of efflux pumps.^196^ In addition, *Enterobacteriaceae* possesses the AcrAB/TolC efflux pump to export pleuromutilins from the cell and avoid their effects. Finally, the lipophilicity of pleuromutilins can reduce their bioavailabilities; prodrugs, however, can improve the permeability of pleuromutilins into cells and, thus, their antibacterial activities. Analogues of pleuromutilin have been prepared to expand the antibacterial scope of pleuromutilins, mostly by modification of the acyl moiety on the C14 alcohol.^197−206^ Some recent work has disclosed methods for modification of the pleuromutilin skeleton in addition to the pendant ester.^207^

Development of newer antibiotic classes has been an ever-evolving field. Several classes of antibiotics have failed during the due course of development and are beyond the scope of the current article, which focuses on emerging antimicrobial strategies. For an article focused on failed antibotics targeting Gram-negative bacteria, please see Prasad et al.^208^ A good resource of antibacterial agents in clinical and preclinical development can be found in an article by Butler et al.^209^

### Alternatives to Conventional Antibiotics

**Figure 2:**
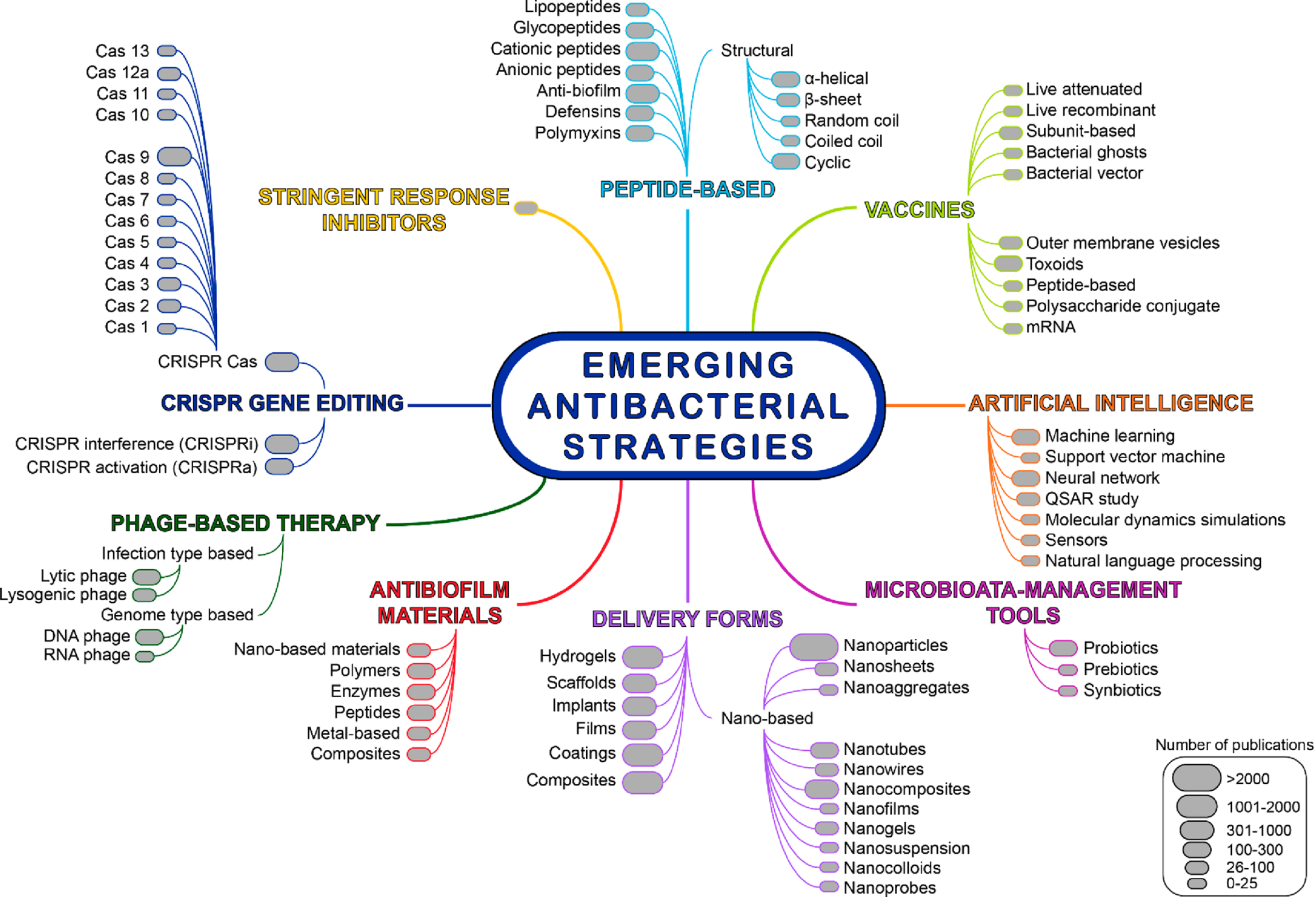
Trend landscape map representing the number of documents (journal and patent publications) from 2012 onward for data retrieved from the CAS Content Collection associated with emerging antibacterial strategies (including emerging forms and newer methodologies used in developing antibacterials).

The continued and growing threat from antibiotic resistance coupled with a lack of newer antibiotics has necessitated the use of alternatives to combat these formidable bacterial infections. Figure 2 shows a trend landscape map representing the number of documents, including journal and patent publications from 2012 onward for data retrieved from the CAS Content Collection associated with emerging antibacterial strategies. The number of documents directly correlates with the interest of researchers in any particular antibacterial strategy or the form of antibacterials being used in the past decade. On the basis of the numbers in the map, a selected few of them are discussed briefly in this section.

#### Stringent Response Inhibitors

Persistent infections affect many; while they are often asymptomatic, the persisting bacteria may be reactivated at any time to cause renewed infection. The quiescent pathogens are termed “persister bacteria.”^52^ Many different mechanisms by which persistent infection is thought to be achieved have been proposed and include stringent response,^52^ SOS response,^210^ toxin-antitoxin response,^211^ and oxidative stress response.^212^ Stringent response is a mechanism by which bacteria counter extreme nutritional starvation (amino acids, fatty acids, iron) and other stresses that allow for survival.^213,214^ Classified as a stress response, the expression and accumulation of guanosine 5′-diphosphate 3′-diphosphate (ppGpp) and guanosine 5′-triphosphate 3′-diphosphate (pppGpp) are the hallmarks of the stringent response.^215^ Both ppGpp and pppGpp, often collectively referred to as (p)ppGpp, are produced by (p)ppGpp synthetase, which includes the RelA/SpoT homologue and small alarmone synthetase proteins.^216^ The exact mechanism by which these molecules achieve stringent response is thought to be varied, one of which includes binding directly to RNA polymerase to lead to decreased transcription.^215,217^ While initially discovered in *E. coli*,^218^ stringent response has also subsequently been identified in many other bacterial species, including *Mycobacterium*(219) and *Bacillus*.^220^ It is now increasingly believed that activation of stringent response might be an important determinant of antibiotic efficacy and might contribute to antibiotic resistance.^221,222^ One avenue that has been explored in recent years is the use of structurally similar compounds, such as 2′-deoxyguanosine-3′,5′-di(methylene bisphosphonate), and analogues leading to competitive inhibition of (p)ppGpp synthetase and decreased production of (p)ppGpp,^223^ which puts a halt to further downstream signaling. Other examples of structurally similar analogues of (p)ppGPpp explored as stringent inhibitors include relacin^224^ and its derivatives.^225^ Peptide-based derivatives that bind to and trigger degradation of (p)ppGpp have also been developed^226^ and were shown to be effective against multidrug-resistant ESKAPEE pathogens.^227^ In recent years, similar efforts have been made for *M. tuberculosis*(219,228) by designing small molecule (p)ppGpp synthetase inhibitors on the basis of ppGpp and relacin^229^ and identification of novel/new leads by screening a 2 million compound library.^230^ Other bacterial strains for which this avenue is being explored include *Neisseria*(231) and *Bacillus*.^232^

#### Bacterial Vaccines

In lieu of the development of novel antibiotics, prevention of bacterial infections via the use of vaccines might be a key alternative strategy available. Additionally, the use of vaccines and prevention or minimization of bacterial infections leads to decreased antibiotic consumption and is, therefore, likely to help with antibiotic resistance.^233^ Finally, by reducing or eliminating drug-resistant strains, vaccines could aid in decreasing antibiotic resistance.^233^ Vaccines designed could either be prophylactic or therapeutic, the latter being useful for preventing the infection from relapsing again and appearing to be more common in the context of tuberculosis.^234^ Vaccine can be composed of (i) live-attenuated bacterial cells, (ii) inactivated bacterial cells, and (iii) a subunit vaccine that contains just enough material from bacterial cells to elicit an immune response and might include specific proteins or polysaccharides.^235^ Finally, inactivated toxins isolated from bacterial cells can also be used to design “toxoid” vaccines;^235^ examples include the DPT vaccine and tetanus vaccine, among others.^236^

A report released by the WHO in 2021 provided details of >60 and >90 vaccines in clinical and preclinical development.^237,238^ The report was focused on identifying vaccines that have been designed for the bacterial strains that are listed in the 2017 WHO Bacterial Priority Pathogens List.^238,239^ The report indicates a lack of vaccines in development for *E. faecium* and *Enterobacter* spp., both of which are classified as high and critical priority in terms of requirement of new/novel antibiotics by the WHO.

Despite obvious benefits, the development of vaccines against multidrug-resistant strains has been slow. In recent years, bacterial vaccine-related research has branched out into the incorporation of nanoparticles for improved delivery,^240^ as well as increased/improved antigenicity.^241^ Another avenue of interest is the development of vaccines against multiple bacterial strains.^242^ The critical role of vaccinations in helping to deal with the COVID-19 pandemic is bound to help generate interest in and accelerate the development of bacterial vaccines, especially mRNA-based vaccines. Indeed, in early 2023, Kon et al. reported an mRNA-based lipid nanoparticle vaccine for the deadly bacteria *Y. pestis* responsible for plague.^243^

#### Antimicrobial Peptides

**Figure 3:**
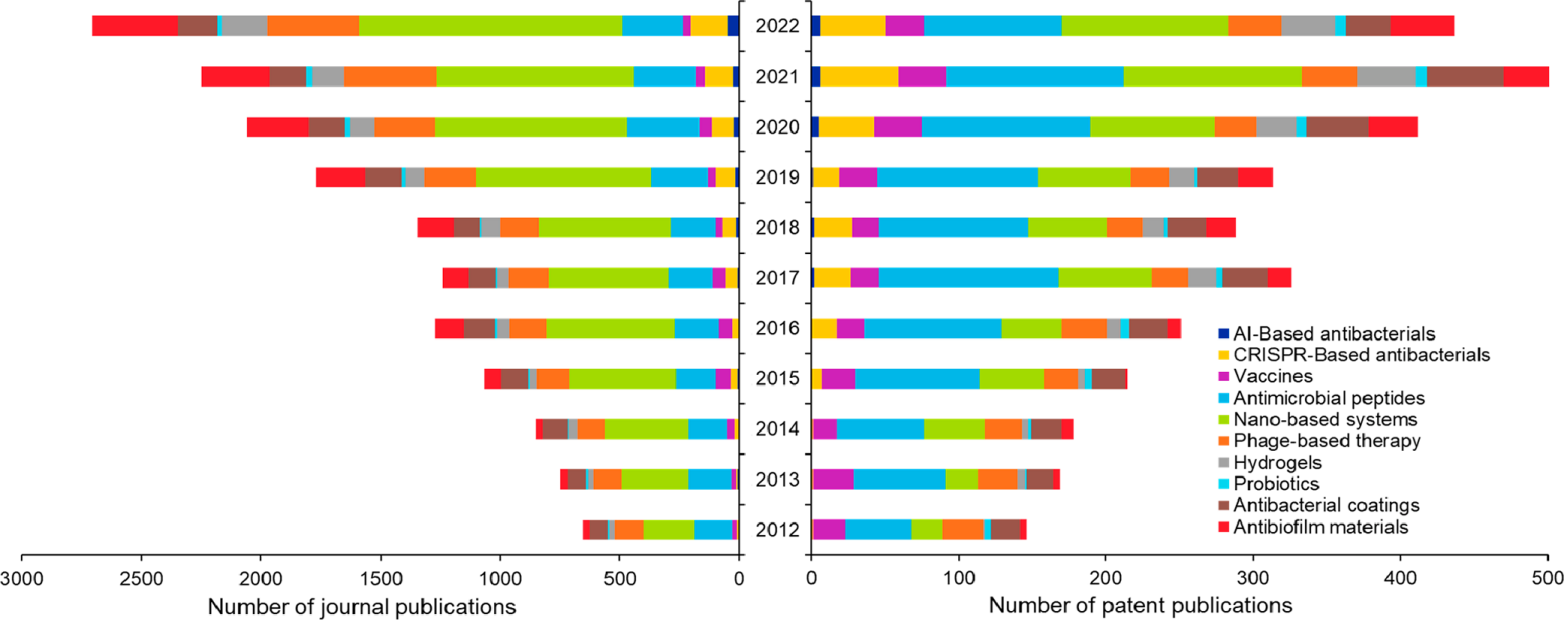
Number of journal and patent publications per year mentioning the use of emerging strategies in antibacterial research over the past decade (2012–2022).

Antimicrobial peptides (AMPs) are gaining popularity in the treatment of drug-resistant bacteria as alternatives for more traditional small molecule antibiotics. They are mostly bioactive proteins naturally produced by all types of living organisms as a host defense system,^244^ though some artificial AMPs have also been synthesized.^245^ AMPs are typically short (<100 amino acids) amphiphilic cationic peptides with a broad spectrum of antimicrobial activity, an overall net charge of +2 to +11 with around 50% of hydrophobic residues, many positive residues (arginine, lysine, histidine), and a molecular weight of <10kD.^244,246−248^ They can be divided into many ways: ribosomally synthesized peptides and nonribosomally synthesized peptides,^249,250^ linear and cyclic peptides,^251^ or on the basis of their secondary structure.^252,253^ In general, AMPs target the cell membranes of pathogens; more details on their mechanisms of actions can be found in reviews by Moretta et al.,^244^ Zhu et al.,^254^ and Zhang et al.^246^ There are over 3000 natural AMPs as of November 2022 according to the Antimicrobial Peptide Database;^255^ some examples of them are glycopeptides, lipopeptides, lipoglycopeptides, lantibiotics,^256−263^ defensins,^264^ and thiopeptides.^265−267^ We will be briefly discussing the first three categories, but for more general information on emerging antibiotic peptides, structure–activity relationship (SAR) studies, strategies to improve AMP activity and biocompatibility, AMP applications, resistance, AMPs in clinical trials, etc., please refer to previous reviews cited in this paragraph. Self-assembled peptide nanomaterials are used to inhibit bacterial growth.^268^Figure 3 suggests that AMPss show a steady growth until 2020 in both journal and patent publications. Interestingly, the growth in patent publications is faster than journal publications, which indicates commercial interest in this area. Notable categories of AMPs are discussed in the following sections.

#### Glycopeptides

Glycopeptides are glycosylated nonribosomal peptides that are composed of a tricyclic or tetracyclic polypeptide scaffold and typically a heptapeptide scaffold made by proteogenic and nonproteogenic amino acids alongside sugar residues, chlorine atoms, methyl groups, or lipid chains.^269,270^ They display antibacterial activity against Gram-positive bacteria typically by inhibiting cell wall biosynthesis due to binding to the C-terminal d-Ala-d-Ala moiety of the peptidoglycan precursor lipid II, which prevents transglycosylation and transpeptidation for cell wall synthesis.^270−274^ They are effective against *S. aureus* (including MRSA), *Enterococcus* spp., *C*. *difficile*, and healthcare-associated infections that are resistant to other antibiotics like *E. faecalis* and *E. faecium*. Some members of approved drugs of this group are vancomycin (1958),^275^ teicoplanin (1988),^276^ telavancin (2009),^277^ dalbavancin (2014),^278^ and oritavancin (2014).^279^ Many novel derivatives and new glycopeptides are also being developed, studied, and optimized.^269−271,280^ Known resistance mechanisms include target site modification, cell wall thickening, enzymatic modification of vancomycin, and efflux pumps.^281^

#### Lipopeptides and Lipoglycopeptides

As the name suggests, lipopeptides consist of a lipid moiety attached to peptide molecules. Daptomycin, which gained US FDA approval in 2003,^282^ remains the only lipopeptide that is currently in use against Gram-positive bacteria.^283^ Structurally, daptomycin is a cyclic lipopeptide consisting of 13 amino acids out of which 10 amino acids form a macrolide ring.^284^ Over the years, SAR efforts have been made to identify structural features required for daptomycin’s antibacterial effect and to try and improve them.^284,285^ Daptomycin functions by disrupting the bacterial cell membrane directly by binding to phosphatidylglycerol^286^ and in an indirect manner by tampering with the synthesis of peptidoglycans^287,288^ with the antibacterial effect observed appearing to be dependent on the presence of and binding with calcium.^287,288^ Other examples of calcium-dependent antibiotics^289^ include lipopeptides isolated and purified from natural sources, such as octapeptins,^290^ friulimicin B,^291^ and amphomycin, among others. Octapeptins, which are cyclic lipopeptides, function by inserting into bacterial cell membranes. They have shown an increase in interest, especially in the last 5 years or so,^292−294^ with efforts being made to systematically study them in order to design newer and more efficacious analogues.^292−294^

Lipoglycocpeptides consist of carbohydrate and lipid moieties attached to peptide molecules. Examples of US FDA-approved lipoglycopeptides include telavancin,^295^ dalbavancin,^296,297^ and oritavancin.^298,299^ The lipoglycopeptide class of antibiotics tends to act via bacterial cell wall disruption by interfering in the synthesis of peptidoglycans^300^ similar to glycopeptides, such as vancomycin. Most likely as a result of the large size, lipoglycopeptides tend to be absorbed poorly upon oral administration and have to be administered intravenously.^301^ They tend to be long-acting with half-lives in the range of several hours.^298,299^ A recent study highlighted lower healthcare costs associated with the treatment of recurrent and serious bacterial infections in individuals with substance use disorder with long-acting lipoglycopeptide,^302^ and a 2020 review described similar outcomes/findings.^303^

#### Bacteriophages

Bacteriophages are viruses capable of targeting and destroying bacterial cells selectively.^304^ While the discovery of bacteriophages can be traced back to the late 1800s, their subsequent development was overshadowed by the discovery and popularization of antibiotics.^304^ Broadly speaking, the lytic cycle of bacteriophages involves the following major steps: (i) attachment to bacterial cells via receptors, (ii) injection of viral DNA into bacterial cells, (iii) replication of viral proteins and components within bacterial cells, and (iv) packing and release of replicated viruses after bacterial cell lysis.^304^ The exact series and mechanism of events may differ depending on the bacteriophage and the host bacterial cell. In contrast, in a lysogenic cycle, incorporation of viral DNA into host DNA occurs.^305^ Bacteriophages tend to be specific in terms of the receptors they interact with and the species they can affect/target.^306,307^ Advantages associated with bacteriophage therapy include effectiveness against MDR bacteria,^306,307^ specificity in terms of species and/or strains, and leaving the patient’s gut microbiome largely unaltered.^308,309^ Furthermore, bacteriophage therapy has been shown to have an excellent safety profile in human beings.^308,310−312^ All of these features mean that as the threat of MDR strains becomes more imminent, bacteriophage therapy has seen a promising resurgence in interest.^313−316^ In a recent study, a group of researchers designed and administered personalized bacteriophage therapy to an individual suffering from lung infection caused by MDR *P. aeruginosa* and appeared to be successful in stopping antibiotic therapy completely.^316^ Other instances of successful personalized bacteriophage therapy against MDR strains have also been reported.^317^ However, there are still challenges that need to be addressed in order to make bacteriophage therapy more viable—poor in vivo efficacy in terms of targeting bacterial species in the gut upon oral administration is an important one.^318,319^ There are also noted instances of resistance against bacteriophages, though their prevalence is far lower than antibiotic resistance.^320,321^

### Microbiota Interventions

#### Probiotics

The use of antibiotics kills not only disease-causing bacterial species but other beneficial bacterial species prevalent in the human gut. Alteration of the complex and dynamic gut microbiota has been increasingly linked to several diseases,^322,323^ including mental health disorders. Furthermore, evidence suggests that disruptions/alterations of the gut microbiome following antibiotic therapy can be long-lasting and anywhere from weeks up to several months.^324^ It has been shown that coadministration of probiotics, live beneficial micro-organisms, along with antibiotics could be beneficial to counter the negative impact of antibiotics on the gut microbiome.^325^ Consequently, this practice is becoming more prevalent; however, there have been concerns raised about the actual benefit of consuming probiotics in rebalancing the gut microbiome.^326^ Micro-organisms that are often administered as probiotics include the bacterial strains *Lactobacillus* and *Bifidobacterium* and can be consumed as part of the diet itself (fermented foods, such as yogurt, sauerkraut, and pickles, among others), or as dietary supplements (in the form of tablets and capsules). After the initial frenzy of interest in probiotics, in recent years, the outlook toward use of probiotics has cooled down considerably. A 2020 study^327^ looked at the use of probiotics in the geriatric population (65 years and older) in a clinical trial comprising 310 individuals and utilized a combination of *Lactobacillus rhamnosus* and *Bifidobacterium animalis* as the probiotic. The study concluded/determined that the use of probiotics could not help reduce administration of antibiotics. Researchers from the Semmelweis University in Hungary published an article in 2023 describing their findings from a meta-analysis of published/conducted clinical trials examining the supplementary use of probiotics in antibiotic therapy.^328^ The analysis concluded that probiotic supplementation was not beneficial during antibiotic administration. As noted by the authors of the 2023 study,^328^ the pool of available data for use of probiotics in antibiotic therapy remains relatively small, and additional comprehensive, systematic and standardized studies are necessary to more effectively examine the role and importance of probiotics.

##### Fecal Microbiota Transplantation (FMT)

Also known more colloquially as stool transplant, FMT is the process of collecting stool samples from healthy donors and transplanting them in the gut of a patient. Used most often in the context of recurrent *C. difficile* infections, FMT is also being explored for other bacterial infections, as well as other diseases, with >500 clinical trials listed on www.clinicaltrials.gov. Interest in FMT as a viable treatment option against *C. difficile* dates back to the 1980s.^329,330^ In 2023, the US FDA approved Vowst, the first fecal microbiota product that can be administered orally.^331^ Meant to be consumed after completion of an antibiotic course, Vowst consists of live bacteria isolated from fecal matter obtained from healthy individuals and is thought to help re-establish gut microbiota.^332,333^

### Importance of Antibiofilm Materials

Biofilms are a complex community of monospecies or multispecies microbes that are attached to a surface and each other and are embedded in a self-produced extracellular matrix that consists of proteins, polysaccharides, and environmental DNA.^334−336^ This allows bacteria to withstand hostile environments, starvation, desiccation, and to be protected from fluctuations in humidity, temperature, pH, etc. Bacteria in biofilms can evade the host defense systems and can cause local tissue damage and acute infection.^335^ These biofilms can develop in catheters, pacemakers, joint prostheses, dentures, contact lenses, prosthetic heart valves, and implants.^334^ Biofilms also protect bacteria and increase bacterial resistance against conventional antibiotics. Dry surface biofilms, which might contribute to healthcare-acquired infections, can be difficult to remove and allow bacteria to tolerate or resist attacks by other pathogens, disinfectants, antiseptics, heavy metals, and other antimicrobial agents.^337^ This means that the development of new antimicrobial materials that also have antibiofilm properties is of utmost importance in the healthcare industry. Specific antibiofilm materials have been developed, such as metal nanoparticles, especially silver nanoparticles, and have been incorporated into various materials, such as catheters, implants, and wound dressings to inhibit biofilm formation.^338,339^ Similarly, chitosan, polyethylene glycol (PEG), graphene, quaternary ammonium compounds (QACs), and natural products have also been explored because of their antibiofilm properties.^340−343^ AMPs can also be used to develop antibiofilm materials since they can be tuned to act on different stages of biofilm formation and have diverse modes of action. They can exhibit antibiofilm properties by acting on different stages of biofilm formation, such as inhibiting bacterial adhesion, preventing quorum sensing within the biofilm, and in disrupting preformed biofilm, which is more difficult than preventing biofilm assembly.^344^ Nisin A and CAMA peptide can disrupt or degrade biofilms of the MRSA strain of *S. aureus*.^345,346^ Similarly, other peptides, such as LL-37, Hepcidin 20, SMAP-29, KT2, and RT2, act on biofilms resulting from *P. aeruginosa*, *S. epidermidis*, *Burkholderia thailandensis*, and MDR *E. coli* biofilms.^347−350^ Certain enzymes, such as deoxyribonuclease I; protease; and the glycoside hydrolases dispersin B, PgaB, PelA, Sph3, amylase, and cellulase alginate, are used as antibiofilm agents.^351,352^

### Emerging Antibacterial Forms

A variety of purposes require prolonged antimicrobial activity or repulsion of microorganisms for which conventional antibacterial administration is less likely to be effective. The use of materials to deliver antibiotics rather than conventional drug delivery methods requires more invasive methods but can provide localized, prolonged, and stimulus-dependent antibacterial activity. Various forms, such as hydrogels, films, coatings, scaffolds, implants, and nanobased forms, such as nanoparticles, are being used to design antibacterial strategies. Medical devices, such as catheters and intravenous lines, can be sources of microbial infection that can potentially be prevented with antimicrobial materials; in addition, the formation of biofilms can impede their functions, thereby making antimicrobial or antibiofilm materials necessary for their continued function. Similarly, implants for bone may be necessary to induce bone regeneration but can also act as sources of infection, which impedes their effectiveness. Surfaces that are touched by many people can act as vectors of infection; antimicrobial coatings on such surfaces can reduce the transmission of microbes. Antimicrobial films can be useful in preventing food spoilage and reducing food waste and food-borne illness. Fabrics with antimicrobial coatings can reduce disease spread and the energy costs and need for cleaning. The forms of materials are important for their activities. Some commonly used examples are described in more detail.

#### Hydrogels

Hydrogels are moldable and injectable materials, and their low density and degradability make them useful for drug delivery and wound healing. Their solvent accessibility also makes them effective at stimuli-sensitive materials. As with many of the materials noted, hydrogels are not inherently antibacterial and require antibiotics or other antimicrobial components to exert antibacterial activity. The surface area of hydrogels can allow them to act in place while being exposed to cells or bodily fluids, which allows either diffusible antibiotics or gel-bound antibiotic agents, such as AMPs, to be used.^353^ The hydrogel material can also protect the antibiotic agents against degradation, thereby allowing them to be more effective at the same dosage or to be equally effective at a lower dosage. Hydrogels are useful as stimulus-responsive materials. Antimicrobial hydrogels may respond to acidity, either reversibly (through conformational shifts) or irreversibly (by chemical reactions, such as hydrazone cleavage). They can also be degraded by enzymes, such as hyaluronidase, which are specific to pathogenic bacteria, or by toxins secreted by bacteria, which enables selective antimicrobial activity. Figure 3 shows that journal publications related to the use of antibacterial hydrogels have shown a constant increase in the last 5 years.

Biologically derived polymers can also be used for antimicrobial hydrogels. Lignin, in particular, has been used because of its broad availability and tunable stiffness that makes a variety of forms accessible.^354^ For example, lignin-containing hydrogels containing silver nanoparticles have been used as antibacterial agents.^355^ A copolymer of lignin with PEG and poly(*co*-vinyl methyl ether-maleic acid) containing curcumin has been shown to be active against *S. aureus* and *P. mirabilis* biofilms.^356^ Lignin-based nanoparticles combined with a poly(oxazoline) triazole have been used as anti-inflammatory agents.^357^

#### Nanoparticles

Nanoparticles are the most used nanobased form in the antibacterial field. The small size of nanoparticles makes them easy to deliver, while their high surface area-to-volume ratio allows them to deliver drugs effectively. Surface modification of nanoparticles can be used to tailor them for specific targets and locations, and the surface chemistry and composition control the timing of activity, drug release, and of duration of action. In addition, alteration of the morphologies of nanoparticles also alters their aggregation, movement, and persistence. All of these properties increase the attractiveness of nanoparticles as antimicrobial agents.^358^ Changes in composition can allow nanoparticle-bound drugs to evade or reduce drug resistance mechanisms; for example, poly(*co*-lactic acid-glycolic acid) (PLGA) nanoparticles containing metronidazole were as effective against juvenile periodontitis as tetracycline, though metronidazole was previously found to be ineffective against the contributing bacterium *Aggregibacter actinomycetecomitans*.^359,360^ PLGA or polyamidoamine (PAMAM) nanoparticles containing platensimycin were more effective against *S. aureus* in mice than free platensimycin and were even effective against MRSA in mice.^361^ PLGA nanoparticles containing azithromycin showed improved activity against MRSA and *E. faecalis* but not *P. aeruginosa*; improvement corresponded to the presence of efflux pump-derived resistance as nanoparticle encapsulated antibiotics are reported to bypass the efflux activity in bacteria.^362^ Metal or alloy nanoparticles can also be effective antibacterial agents. Silver nanoparticles have been used to prevent bacterial growth and treat infections, but their toxicity may limit their use.^363^ Copper nanoparticles also show antimicrobial activity.^364^

#### Films or Coatings

Films occlude microbial access to surfaces, thereby preventing their adherence, preventing biofilm formation, and killing bacteria. Hospitals are high-traffic areas with objects and surfaces being handled/touched by many people, which makes them foci of disease spread. Antibacterial copper nanoparticle-containing coatings have been suggested for application to surfaces in hospitals, such as bed rails and chairs, to reduce the viability of bacteria and viruses on those surfaces.^365^ For example, poly(ethylene glycol diacrylate) films containing copper nanoparticles were prepared as antibacterial films.^366^

Catheters and intravenous lines are also common sources of infection: they can carry bacteria from the environment into patients, bypass the defenses of the skin and mucous membranes, and increase the population of microbes in people of special concern, i.e., immuno-compromised individuals, who have reduced energy or resources to fight off infection. Reducing the ability of medical devices to transmit infection would be an effective way to improve the health and survival of hospital patients. To this end, polycationic polymers (including quaternary ammonium salt-containing polymers), zwitterions, PEGs, and antibacterial peptides are promising materials to be used as antibacterial coatings for the prevention of medical device-associated infections.^367−369^ Flat surfaces can be used to harvest UV and visible light and used to generate reactive species, such as singlet oxygen. While UV light is lethal to many microbes, it is also harmful to human and animal cells, so materials that can use visible light to generate reactive species are preferable. A photoactive polymer was prepared and shown to generate singlet oxygen, which killed nearby cells.^370^ The anatase form of titanium dioxide (TiO_2_) generates reactive oxygen species upon irradiation, which are toxic to microbial cells.^371^ This activity also underlies the use of TiO_2_ in self-cleaning window coatings.^372^

Antimicrobial films may also serve other purposes. Antifouling coatings can be formed without the use of antimicrobial agents by the generation of superhydrophobic surfaces in which the feature sizes (on a micron or nanometer scale) and shapes prevent both water and other solvents from binding effectively to the surface. Surfaces that can repel water and other solvents can also prevent dirt and microorganisms from adhering to a surface.^373−375^ Superhydrophobic films can also be used on fabrics to repel water and dirt, thereby reducing their need for laundering, but previous coatings have used fluorinated polymers whose degradation products, intermediates, and precursors are persistent pollutants with unknown toxicities, which deprecates their use. Antibacterial films can also be used to reduce bacterial degradation of food, which would reduce food waste. Edible films using chitosan, starches modified to improve their durability in the presence of water, carboxymethylcellulose and cyclodextrins, pectin, zein, whey protein, and the Maillard adducts of soy protein and carbohydrates have been tested for food preservation to preserve food while reducing fossil fuel use.^376^

#### Scaffolds and Implants

Networks also have a high surface area-to-volume ratio but are localized to specific sites and generally are more persistent than hydrogels. They are useful as substrates for cell growth and, thus, are useful for wound and bone healing. For wound healing, networks of chitosan^377^ and sodium alginate with poly(vinyl alcohol) (PVA)^378^ can both facilitate healing and inhibit infection. Bone matrixes require yet more persistence to allow the growth of new bone and greater rigidity because of the stiffness of bone. Antibacterial agents are important because bone infections are likely less accessible to antimicrobial agents and, thus, are more difficult to treat; preventing them would be more efficient than treating them. One example is a gentamicin-containing porous implant for bone healing;^379^ a quaternized chitosan/polyester/hydroxyapatite scaffold was also implanted in rats and rabbits and had antibacterial and bone-healing activities.^380^ Implants using cationic polymers^381^ or copper nanoparticles were effective at preventing infections, and the copper/polyetheretherketone implant was effective against MRSA.^382^ An alternative antibacterial method is the use of nitric oxide-releasing agents in concert with bone matrixes to kill microbes.^383^

#### Composites

Composites use multiple materials in concert. One example of an antibacterial composite is the combination of copper compounds with an anion-exchange resin to kill bacteria in water for purification.^384^ The addition of tetrachlorocuprate(II) salts to an anion exchange resin and reduction with ascorbic acid yielded a composite resin containing Cu_2_O; exposure of Gram-positive *E. faecalis* to the material reduced bacterial load by 10^5^, while the resin did not affect Gram-negative *E. coli*. Antimicrobial composite materials are useful for medical devices, such as dental implants, where infections may be difficult to treat or may cause secondary structural damage. For example, silver and zinc oxide nanoparticle-containing composite resins for dental use were tested and inhibited *Streptococcus mutans* and *Lactobacillus* species.^385^ Polylysine was incorporated into a dental composite to prevent caries-induced demineralization and repair failure,^386^ and noncovalent assemblies of *N*-Fmoc-pentafluorophenylalanine bound to a dental resin reduced bacterial growth of *S. mutans* at 0.25–1% concentrations and nearly abolished it at 2% concentration.

The variety of materials capable of exerting antimicrobial activity provides options not only for treating microbial infection but also for preventing microbial transmission and infection. They are also capable of reducing some of the other effects of bacterial and microbial growth, such as fouling and food spoilage. The alternative antibiotic delivery systems are likely to have disadvantages, as well. Hydrogels and nanoparticles are likely to require delivery by injection and, thus, administration by medical professionals. The rate of drug release from hydrogels is difficult to control because of their rapid degradation. Their mechanical stabilities also are low (greater stiffness would make their delivery by injection difficult) but can be improved by alteration of the polymers in the hydrogel framework. Nanoparticles require specialized coatings for treatment of specific organs and infections. Metal nanoparticles are likely toxic to bacteria through their generation of reactive oxygen species;^387,388^ while this mechanism is less amenable to evasion by bacteria, metal nanoparticles also can potentially be toxic to normal cells and to cells upon emission into the environment. Antibacterial films (if used internally), implants, and composites are mechanically stable, but the release of coatings or fragments from films or implants may lead to undesired toxicity. Implants and devices with films require surgical implantation, and if the coatings or implants either no longer show antibiotic activity (either through resistance or by depletion of the antibiotic component) or are toxic, they will require surgical removal. If the mechanism of action of the antibacterial films is susceptible to resistance, their durability prolongs the exposure time and, thus, potentially the frequency of resistance.

While materials are subject to evolutionary strains in microbes and, thus, require monitoring, they provide broader and longer-term means to deal with a variety of problems related to microbial growth and infection. Various emerging antibacterial forms and therapeutic strategies are currently in clinical trials (Supplementary Figure 1 and Supplementary Table 2).

## Landscape View of Antibacterial Research—Insights from CAS Content Collection

The CAS Content Collection^8^ is the largest human-compiled collection of published scientific information representing a valuable resource to access and keep up to date on scientific literature with over 59 million records across disciplines, including chemistry, biomedical sciences, engineering, materials science, agricultural science, and many more, from all over the world. Comprehensive data from the CAS Content Collection allows quantitative analysis of global research publications across various parameters, including time, geography, scientific area, medical application, disease, and chemical composition. To apprehend the research landscape for antibacterials in the past decade, a search query was developed to extract the data set and was analyzed extensively to give insights into publication trends, patent activities, CAS-indexed concepts, and substances.

**Figure 4:**
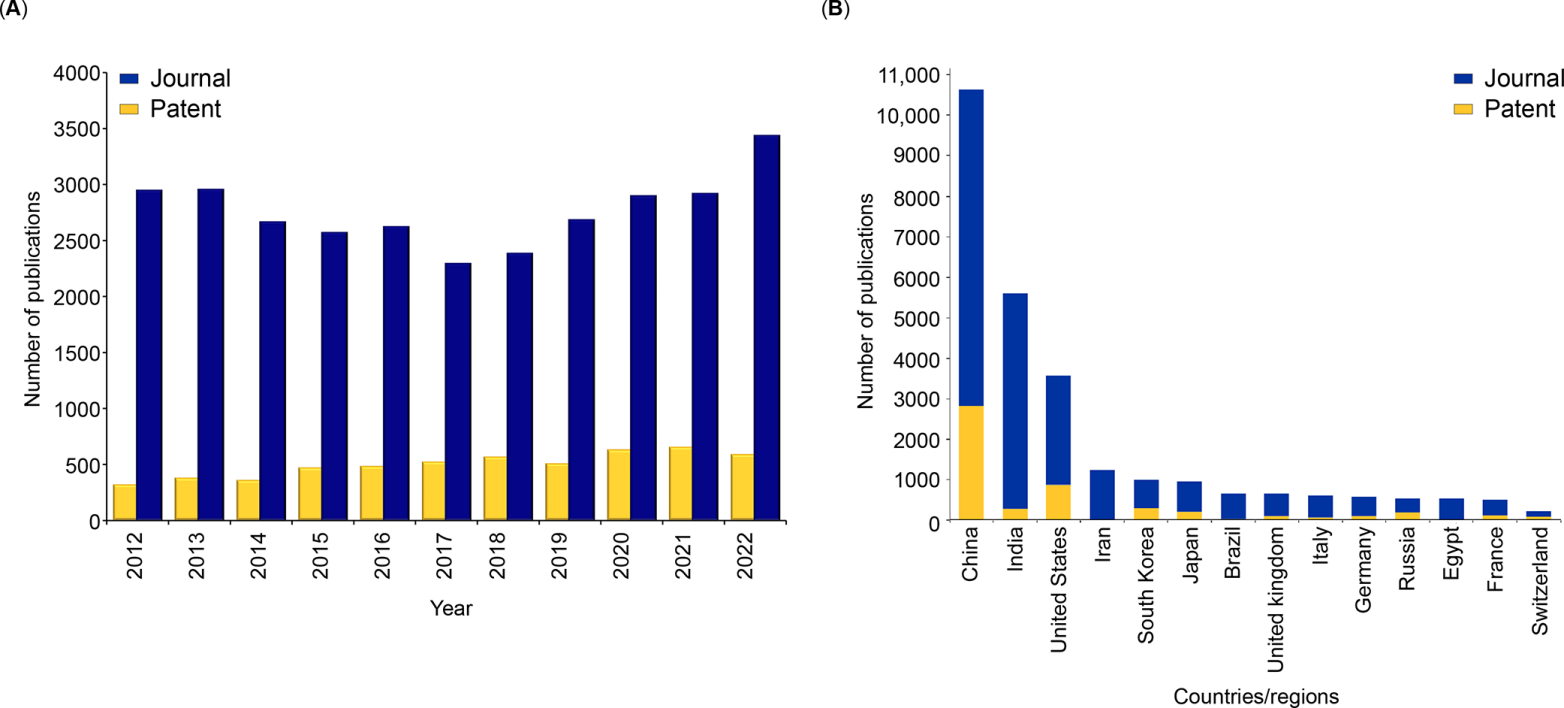
(A) Number of journal and patent publications per year in the field of antibacterial research (shown as blue and yellow bars, respectively) over the past decade (2012–2022). (B) Top countries/regions for the numbers of antibacterial-related journal articles (blue bars) and patents (yellow bars) over the past decade (2012–2022).

In the past decade, there have been over 35 000 scientific publications (mainly journal articles and patent publications) related to antibacterial research in the CAS Content Collection indicating continual research, development, and commercialization efforts being made in this field. Journal publications dominate the field, while patent publications amount to one-fifth of the journal publications. This trend suggests that vast amounts of academic research in the past decade have not yet resulted in commercialization. There has been an overall growth in journal publications over the last 5 years with a >15% increase in the last year (Figure 4A) correlating well with the post-COVID19 increase in nosocomial infections.^7^ China, India, the United States, Iran, and South Korea are the world leaders with respect to the number of journal publications (Figure 4B); China has nearly twice as many publications as India. It is noteworthy that Iran, India, and Italy have a much higher number of published journal articles than patent publications, while China has ∼3-fold greater number of journal and patent publications, respectively, than the United States, which indicates differential allocation of research funds in each country or region.

**Figure 5:**
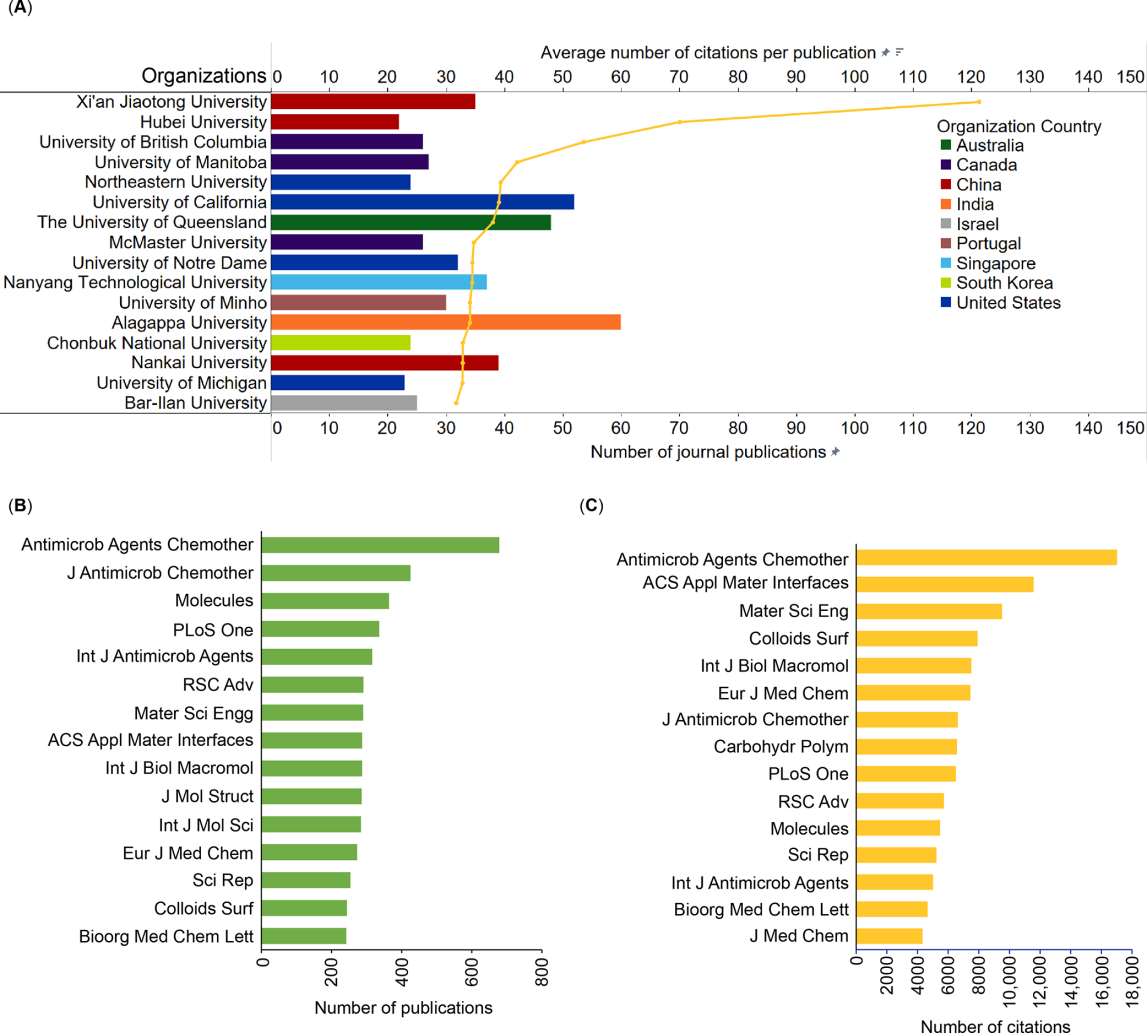
(A) Top research institutions in terms of average citation numbers per journal publication between 2012 and 2022. The colors of the bars represent the institution’s country/region: red (China), blue (USA), indigo (Canada), green (Australia), light blue (Singapore), brown (Portugal), orange (India), light green (Republic of Korea), and gray (Israel); the yellow line represents the average number of citations per publication. Top scientific journals with respect to (B) the number of antibacterial research-related articles published and (C) the number of citations they received for the period 2012–2022.

We identified leading organizations for journal publications in research related to antibacterials (Figure 5A) with respect to both the number of journal publications and the average number of citations per publication (an indicator of the influence of that publication in the field). Unsurprisingly, research institutes from the United States and China account for nearly half of the top journal publications and are followed closely by institutes in Canada. One institute each from India, Israel, Portugal, South Korea, and Australia features in the list of top institutes. The journal *Antimicrobial Agents and Chemotherapy* appears to publish the highest number of articles related to antibacterial research (Figure 5B) and is the most cited journal in the field (Figure 5C).

**Figure 6:**
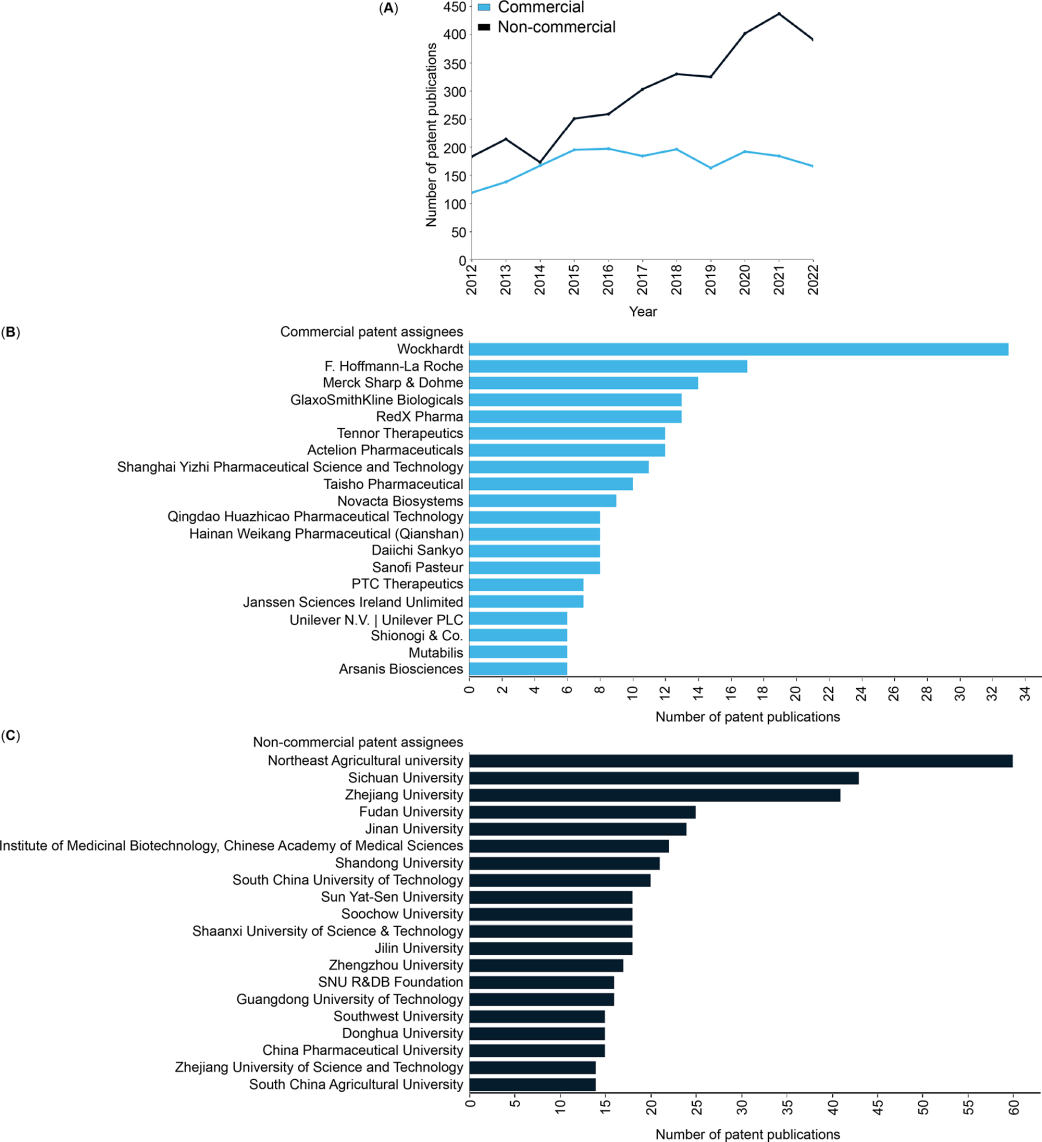
(A) Number of patent publications per year between 2012 and 2022 by commercial (blue) and noncommercial (black) assignees. Top 20 (B) commercial assignees and (C) noncommercial assignees with respect to the number of antibacterial research-related patents published from 2012 to 2022.

**Figure 7:**
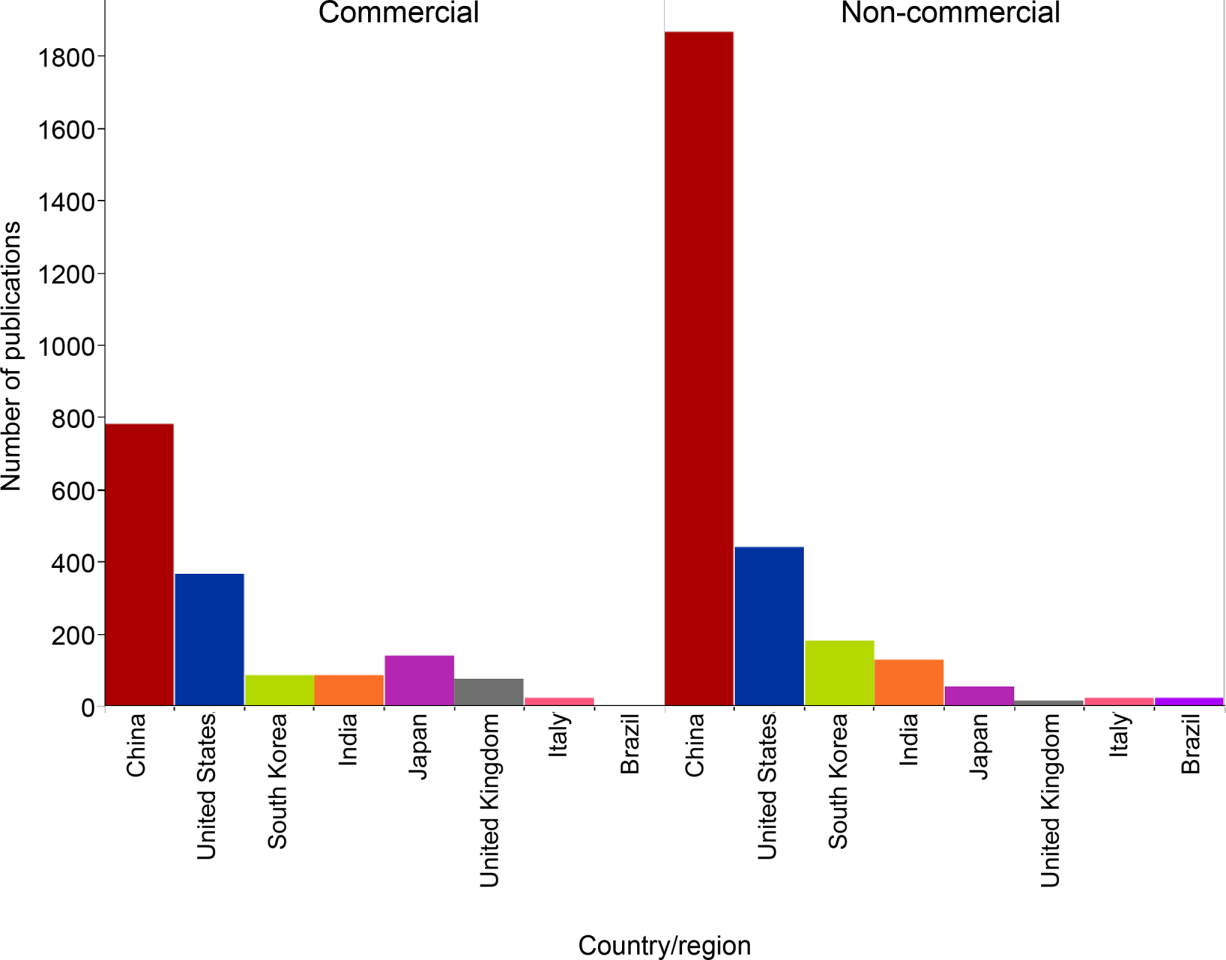
Distribution by country of patent publications for commercial assignees (left panel) and noncommercial assignees (right panel). The colors of the bars represent the organization’s country/region: yellow (China), blue (USA), light blue (Republic of Korea), orange (India), magenta (Japan), gray (United Kingdom), and pink (Israel).

Patent publications were analyzed to identify leading patent assignees and their geographical distribution. In terms of the number of patent publications, patents by noncommercial assignees outnumber commercial ones, which indicates that noncommercial organizations are engaged in more antibacterial research and are trying to find ways to patent and commercialize them. Interestingly, the number of patents by noncommercial assignees has shown a steady increase in the past decade, while the number remains more or less steady for commercial assignees (Figure 6A). China dominates patents in the field of antibacterials as it has the highest number of commercial and noncommercial patent assignees (Figure 7). Chinese universities account for all of the top 15 spots in the top noncommercial assignees. Unsurprisingly, the number of patents by noncommercial assignees from China is ∼4 times higher than the USA and ∼3 times higher than that of South Korea. China, the USA, Japan, Korea, India, the UK, and Italy are the top assignees for commercial patents. Wockhardt Limited, the leading commercial organization in the field of antibacterials, has notable patents on the use of nitrogen-containing compounds as antibacterials.^389,390^ Other companies, such as F. Hoffmann-La Roche, have patents related to sequence-specific antibacterial testing^391^ and peptide macrocycles against drug-resistant strains of *A. baumannii*,^392^ among many others. Notable journal and patent publications from recent years are included in Supplementary Table 3 and Supplementary Table 4, respectively.

**Figure 8:**
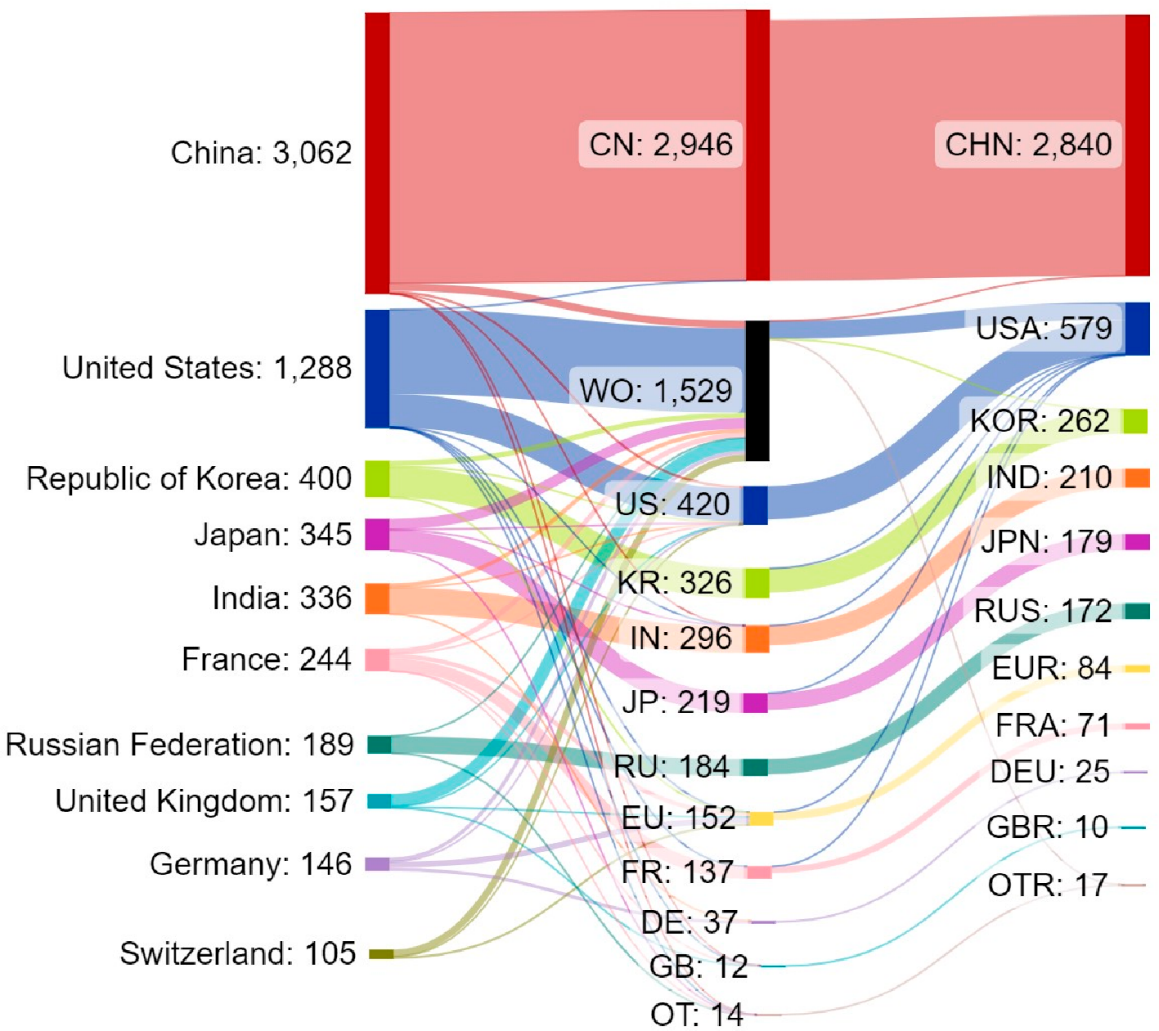
Patent flow of antibacterial-related patent filings from different assignee countries/regions to various patent filing offices (center) and final destination patent office (right). The abbreviations in the center and right indicate the patent offices. Standard two- and three-letter codes are used to denote country names corresponding to their patent offices.

Patent protection is influenced by the country/region of the applicant; consequently, the same invention can be filed for patent protection in several jurisdictions or it can be filed through the World Intellectual Property Organization (WO) and later filed to patent offices in different countries. This accounts for certain patent families being counted more than once, which represents them being filed at multiple patent offices. Figure 8 represents a chronological flow of filing individual patent applications within patent families in various national patent offices, the World Intellectual Property Organization, and the European Patent Office (EP). The left column shows the top 10 patent assignee countries/regions in terms of the number of patent activities (here, an activity is defined as an event where a patent document, either an application or a granted patent, is published). The extreme right column shows the patent office where the patent activity took place. The center column connecting the two indicates the office where the first patent in the family was filed. Unsurprisingly, China and USA have the highest patent flow activity, which correlates well with their high patent numbers. Interestingly, most countries tend to have a higher number of patent filings at their home country’s patent office followed by their initial filings at the WO.

**Figure 9:**
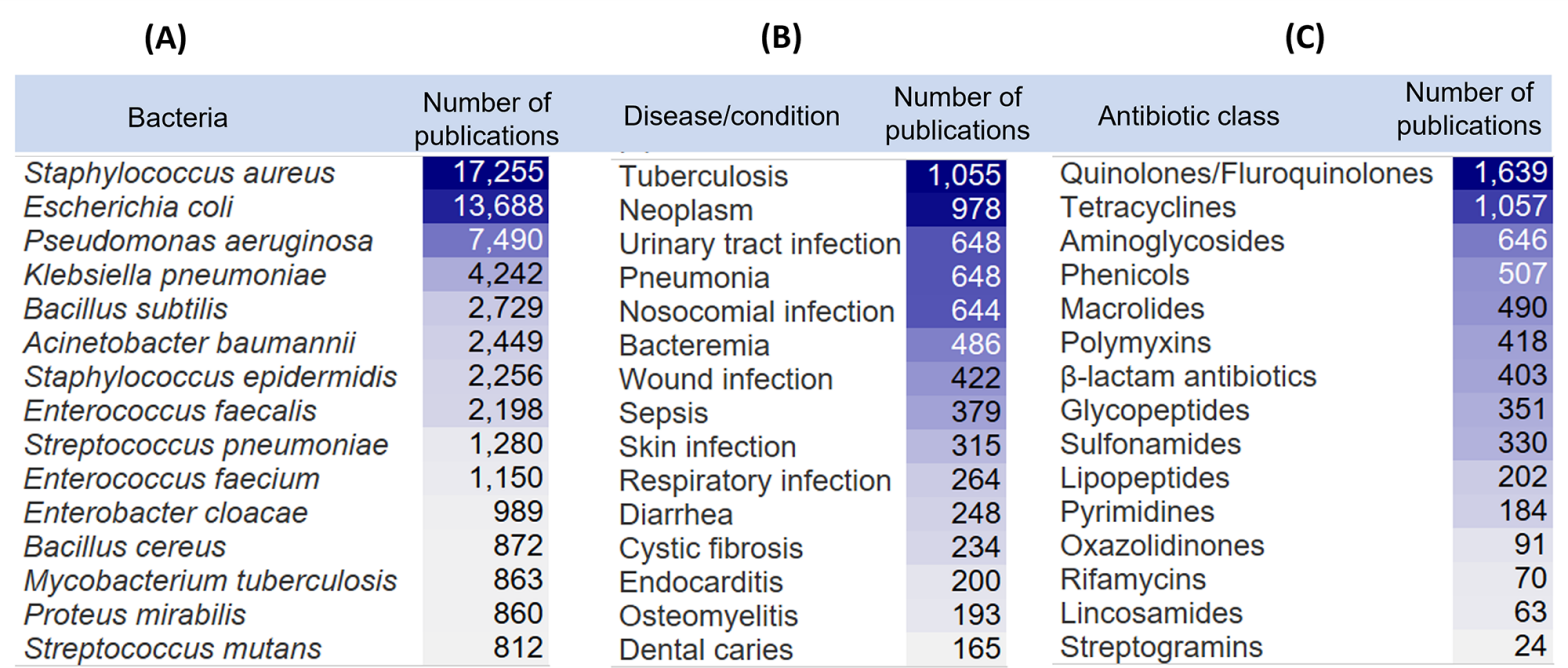
Heat map tables indicating number of publications mentioning the top (A) bacterial species, (B) diseases/conditions caused by bacteria, and (C) antibiotic classes used in the field of antibacterials.

We further explored distribution and trends in the published documents (journals and patents) dealing with various antibacterial-related concepts. Figure 9A shows the number of publications corresponding to the most prominently occurring bacteria in the field of antimicrobials. *S. aureus* shows the maximum number of publications followed by *E. coli;* this is unsurprising as these microorganisms are the most common causes of hospital-associated infections and bacteremia (the presence of bacterial infection in the blood) in predisposed populations.^393,394^ MRSA remains a prominent cause of bacteria-related deaths worldwide.^326^ Interestingly, all the bacteria from the “ESKAPEE” list, including *E. faecium, S. aureus, K. pneumoniae, A. baumannii, P. aeruginosa, Enterobacter* spp., and *E. coli*, feature in this list, which indicates that significant research efforts are being directed toward combating these bacteria.^395^ In terms of the number of publications mentioning specific bacterial diseases or conditions, tuberculosis was the most common bacterial disease found (Figure 9B). This is consistent with the frequency of indexing of bacterial species in which *M. tuberculosis* is most often seen in publications (Figure 9A). Urinary tract infections, nosocomial, and respiratory infections have also been frequent subjects of published research (Figure 9B). Quinolones and fluoroquinolones appear to head the top antibiotic classes followed closely by tetracyclines and aminoglycosides (Figure 9C).^396^

**Figure 10:**
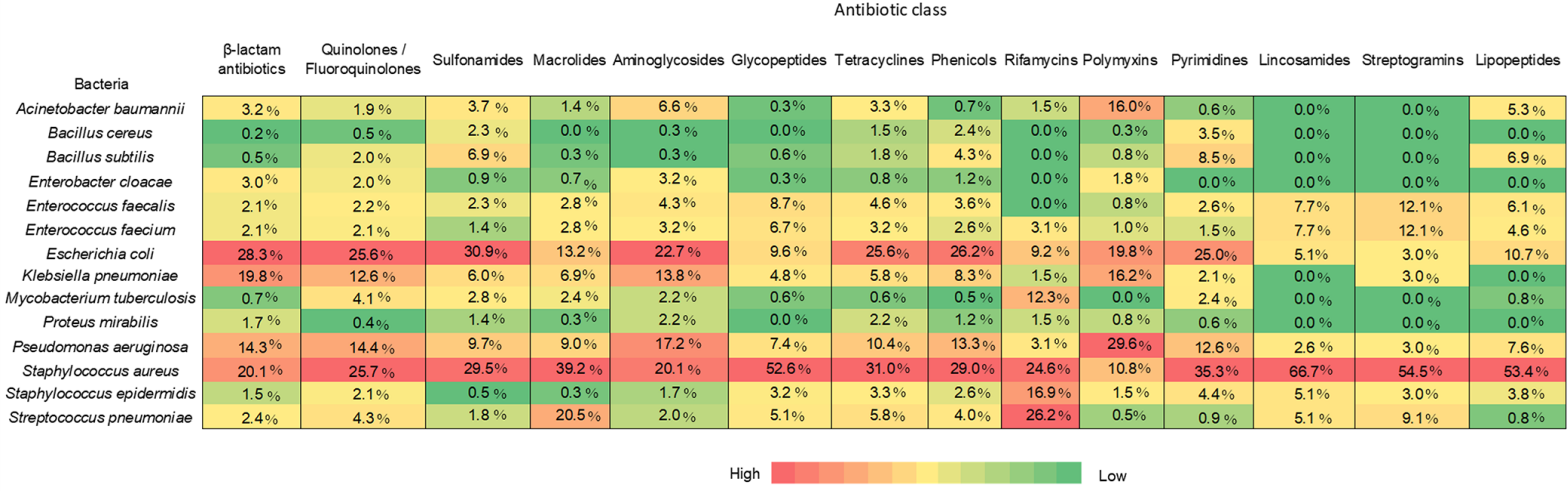
Heat map of the relationship between the most used classes of antibiotics (top) and prevalent bacterial species (left) in the field of antibacterials. Data comprises journal and patent publications obtained from the CAS Content Collection for the period 2012 to 2022. Relative frequencies of each bacterial species have been calculated within each class of antibiotics.

To understand the co-occurrence of major classes of antibiotics and various bacterial species, we generated a heat map as shown in Figure 10. Here, the relative frequencies of each bacterial species have been calculated within each class of antibiotics and are indicative of the relationship between each antibiotic class and the top species of bacteria. Overall, *S. aureus* and *E. coli* have the highest relative frequencies for each major antibiotic class, which indicates a higher amount of research documents present for these bacteria. Certain classes of antibiotics are selectively effective against Gram-positive or Gram-negative species. For instance, aminoglycosides are documented to be more effective against Gram-negative bacteria, particularly *E. coli*, *K*. *pneumoniae*, and *P. aeruginosa*, which comprise more than 50% of co-occurrences. Similarly, a higher use of polymyxins against Gram-negative bacteria, especially *A. baumannii* and *K. pneumoniae*, correlates with the literature.^397^ However, lipopeptides and glycopeptide-based antibiotics have higher document frequencies with Gram-positive bacteria, such as *S. aureus*.^396^

**Figure 11:**
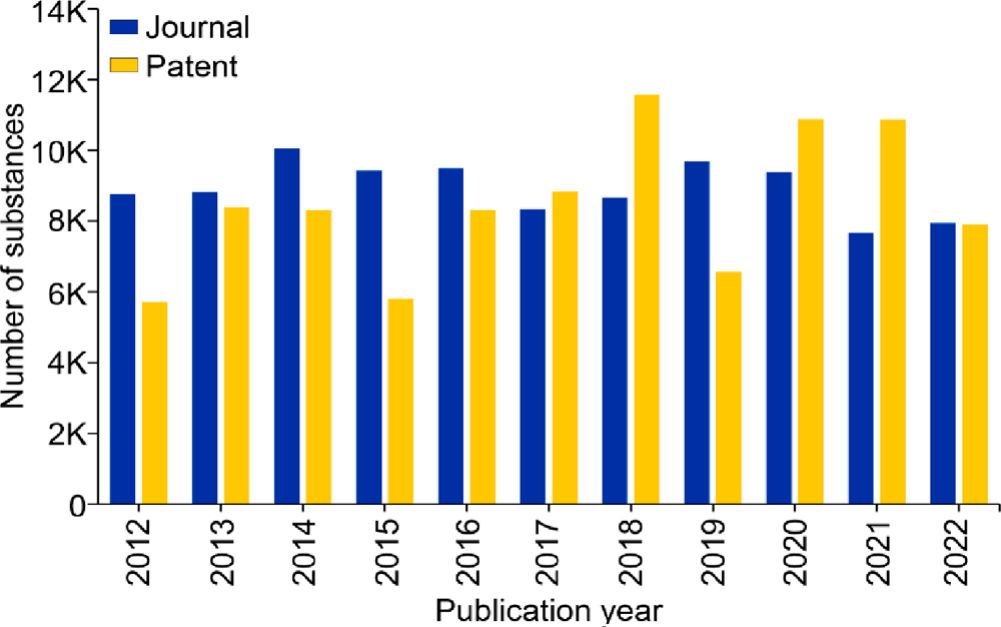
Growth in substances associated with antibacterials over 2012–2022 from the CAS Content Collection. Only substances indexed with a therapeutic (THU) or pharmacological activity (PAC) role were included for the analysis.

Data analysis for substances in the field of antibacterials for the past decade depicts a steady number over the years. Substance analysis was confined to relevant roles, including therapeutic (THU) and pharmacological activity (PAC). Figure 11 represents the growth of substances associated with the antibacterial field in the past decade. In the initial years, the number of substances reported in journal publications was higher than the number reported in patent publications, but the trend reversed between 2018 and 2020. Interestingly, the number of substances reported in journals and patents is nearly identical in 2022.

**Figure 12:**
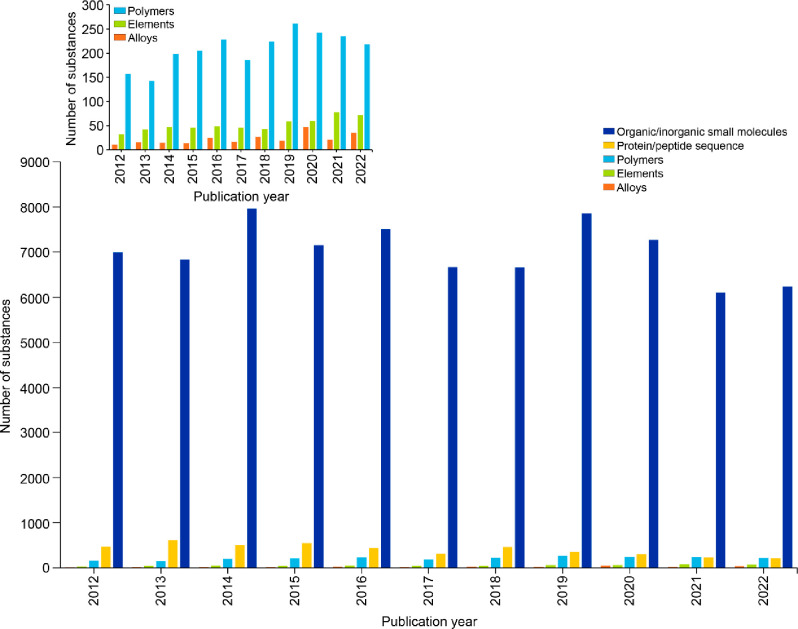
Distribution of substances associated with antibiotics over 2012–2022 from the CAS Content Collection. Only substances indexed with a therapeutic (THU) or pharmacological activity (PAC) role were included in the analysis. Heat map tables list the top 10 substances co-occurring in those specific classes.

**Figure 13:**
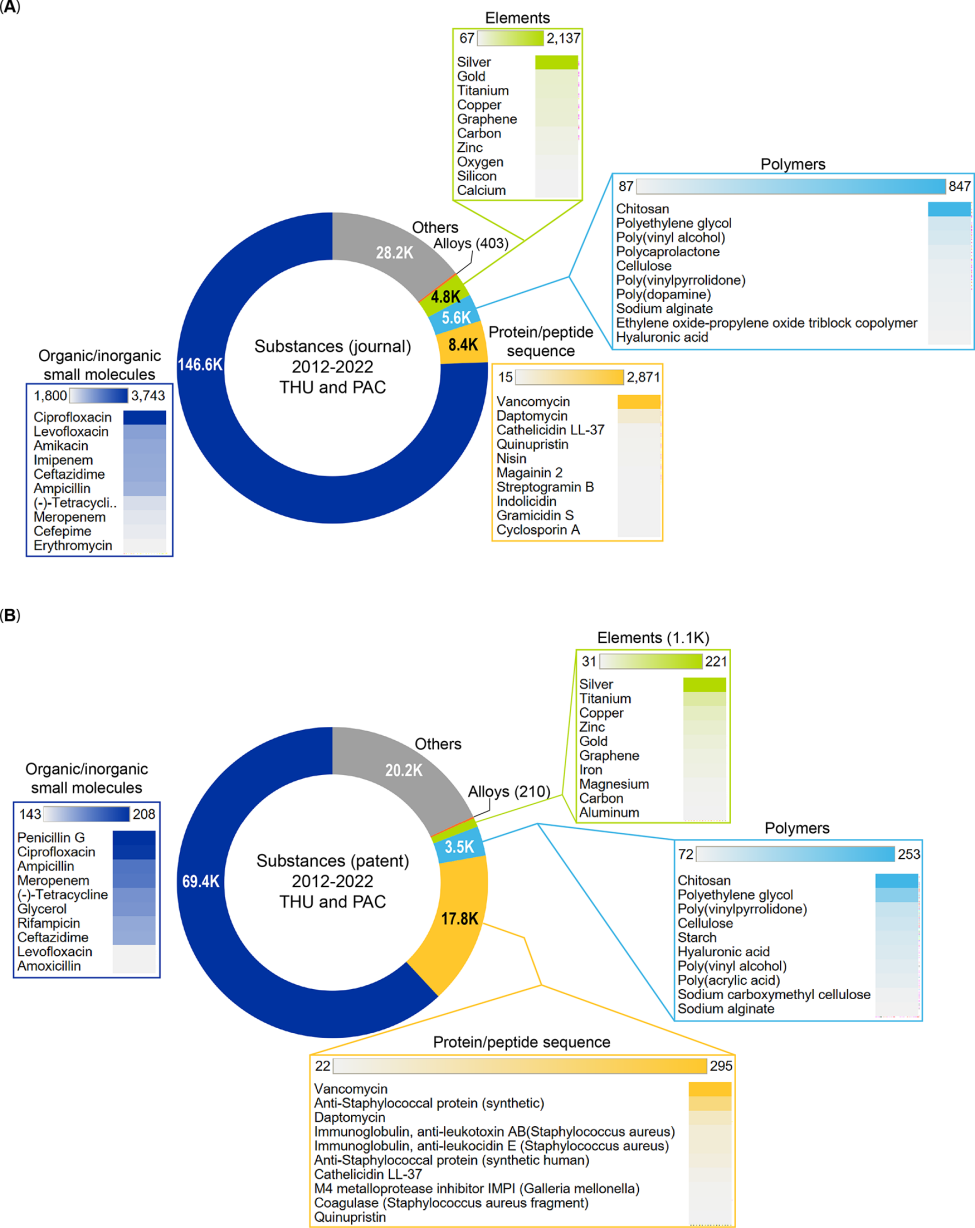
Number of substances of different classes associated with journal publications of antibiotics over 2012–2022 from the CAS Content Collection. Only substances indexed with a therapeutic (THU) or pharmacological activity (PAC) role were included for the analysis. Inset graph shows a zoomed in view with an emphasis on polymers, elements, and alloys to better reflect growth over the past decade.

Further investigation into the classes of substances suggests that organic and inorganic small molecules, protein/peptide sequences, polymers, elements, and alloys are the major classes of importance in the field of antibacterials. Figure 12 represents the growth of various substance classes in the past decade. The number of substances classified as organic and inorganic small molecules is 40–50 times higher than the next class of substances—protein/peptide sequences. Other classes, such as polymers, elements, and alloys, while important, still account for a much smaller fraction of substances being used in the field of antibacterials. Among the major classes, organic/inorganic small molecules show a marginal decrease post-2020, thereby indicating the shift in interest from small molecules toward more novel/alternative forms of antibiotics, such as materials and forms. Figure 13 depicts the distribution of substances from journal and patent publications, respectively. Overall, the distribution varies slightly between journals and patents where the percentage of small molecule substances is slightly less in patents when compared with journal publications, whereas peptide-based substances are reported more in patent publications.

As seen in Figure 13, there are over 216 000 small molecule substances associated with publications in our data set. Among the small molecule category, ciprofloxacin and levofloxacin (both belonging to the quinolone class of antibiotics) have the highest number of occurrences, and this agrees with Figure 9C wherein the number of publications for quinolones and fluoroquinolones were the highest. Other antibiotics featured in the list belong to various classes, such as β-lactam antibiotics (imipenem, ceftazidime, ampicillin, meropenem, cefepime, penicillin, etc.), aminoglycosides (amikacin), and macrolides (erythromycin), among others. Among the proteins/peptides found in the field of antibacterials, a total of ∼26 000 substances have been reported. Unsurprisingly, peptide-based antibiotics, such as vancomycin—a glycopeptide antibacterial^398^—exhibit the highest number of occurrences followed by the lipopeptide antimicrobial, daptomycin.^399^ AMPs, such as cathelicidin LL-37,^400^ nisin,^401^ magainin 2 (MG2a),^402^ and streptogramin B,^403^ among others, also feature in top protein/peptide substances. Polymers with antibacterial properties have various advantages over their small molecule counterparts, such as higher efficacy, reduced toxicity, fewer environmental problems, and less susceptibility to antimicrobial resistance.^404^ Natural polymers, such as chitosan, cellulose, and starch, and synthetic polymers, such as PEG, PVA, and polycaprolactone (PCL), are among the top-ranking polymer-based substances in this field. Metals, such as silver, gold, titanium, gold, etc., feature among the top element-based substances. Substances from other categories, such as ceramics, plastics, and MXenes, are also used in the antibacterial field, which indicates substance diversity.

**Figure 14:**
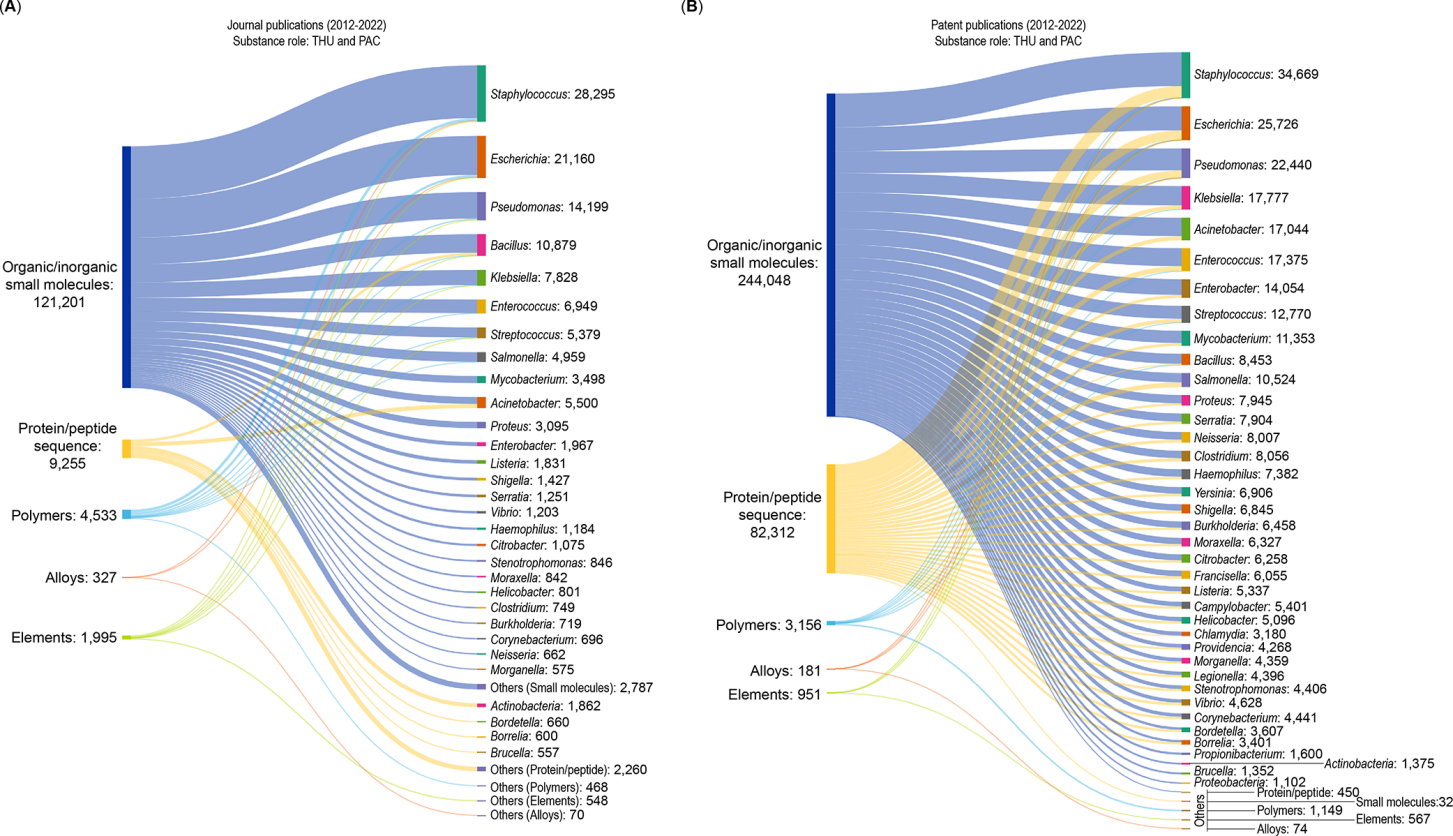
Sankey graphs indicating co-occurrences between different classes of substances and various bacterial genera in (A) journal and (B) patent publications from the CAS Content Collection for the period 2012–2022. Only substances indexed with a therapeutic (THU) or pharmacological activity (PAC) role were included in the analysis.

Correlation between various substance classes and different bacterial genera is shown as a Sankey graph for journal and patent publications (Figure 14). *Staphylococcus, Escherichia, Pseudomonas, Klebsiella*, and *Bacillus* have the highest number of reported substances associated with both journal and patent publications. Interestingly, a greater number of protein/peptide-based substances associated with journal publications appear to be focused on *Acinetobacter* and *Actinobacteria*, while for patent publications, *Staphylococcus, Escherichia*, and *Pseudomonas* are the top bacterial genera.

**Figure 15:**
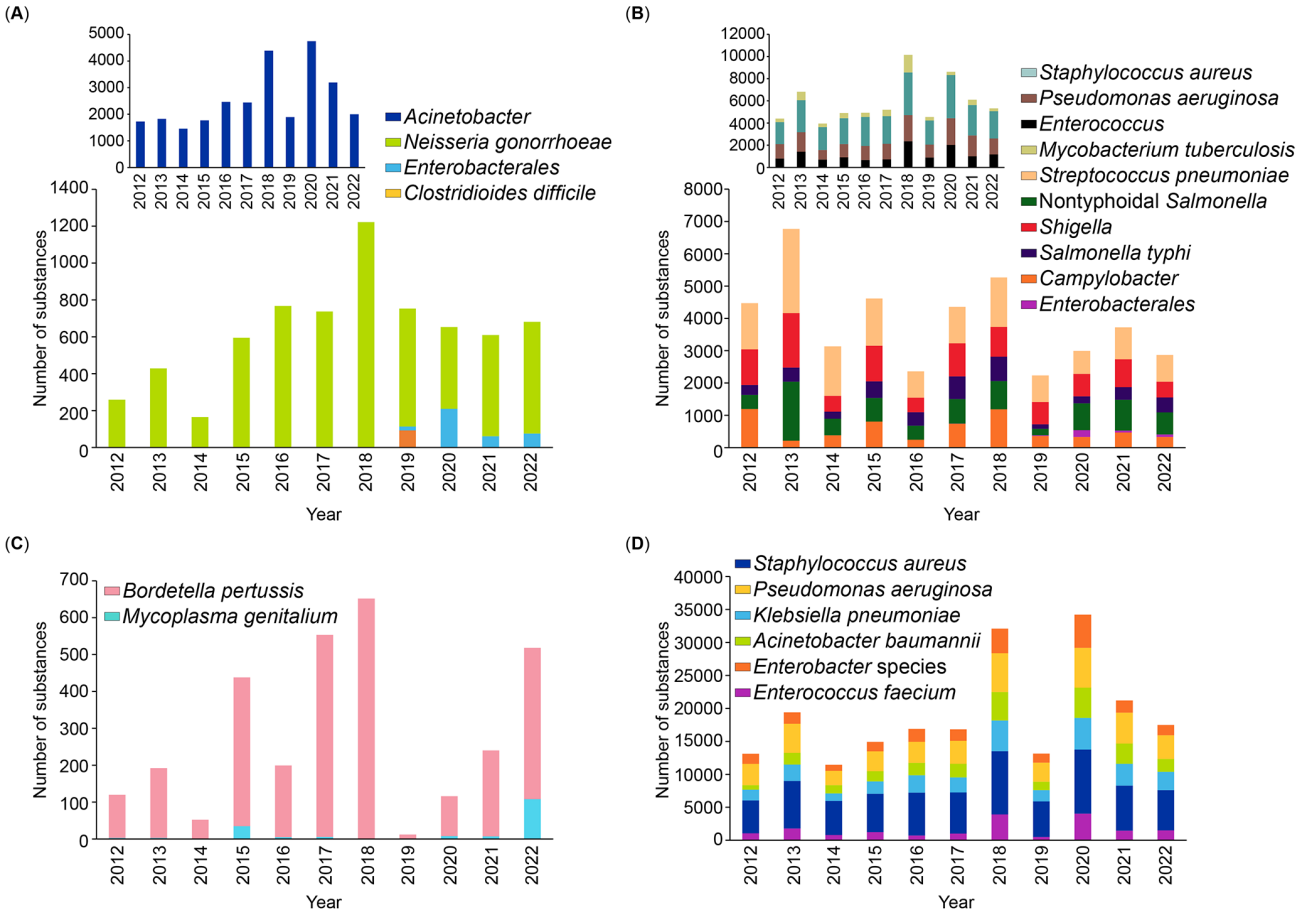
Growth in substances for bacterial strains recognized as (A) CDC’s urgent AMR threat, (B) CDC’s serious AMR threat (C) CDC’s AMR watchlist, and (D) ESKAPEE pathogens from the CAS Content Collection for the period 2012–2022. Only substances indexed with a therapeutic (THU) or pharmacological activity (PAC) role were included in the analysis.

Since antimicrobial resistance is a growing threat, the CDC has maintained a list of microbes that could represent urgent antimicrobial resistance (AMR) threats, serious AMR threats, or AMR watchlist (microbes that could become serious threats in the future because of their propensity of becoming MDR) in 2019.^23,405^ These lists serve as strategic tools to prioritize and address the most pressing antimicrobial threats. Figure 15 represents the growth of substances associated with bacteria belonging to each of these lists from journal and patent publications in the past decade. Figure 15A shows growth for bacteria from the CDC’s urgent threat list comprising drug-resistant *Acinetobacter, Neisseria gonorrheae, Clostridioides difficile*, and Enterobacterales. *Acinetobacter* has the highest number of reported substances. Substances for *N. gonorrheae* have shown more or less steady growth in the past three years. Figure 15B represents substance growth over the years for bacteria in the CDC’s serious threat list. The highest number of substances are reported for *S. aureus, P. aeruginosa, Enterococcus*, and *M. tuberculosis*. Almost all bacterial species show sustained interest with the number of substances associated with them being steady. *Salmonella typhi*, in particular, appears to show a modest and steady increase in the number of substances for the last three years. Figure 15C depicts substance growth over the years for bacteria in the CDC’s watchlist. Interestingly, *Mycoplasma genitalium* shows a spike in the number of substances in 2022. *M. genitalium is* the causative agent for urethritis in men (urethral inflammation) and cervicitis in women (cervical inflammation) and is resistant to azithromycin. *B. pertussis*, meanwhile, is responsible for whooping cough and shows a steady increase in substances over the last three years, nearly doubling in 2022, which is indicative of interest in this direction. Finally, Figure 15D represents substance growth in the last 10 years for ESKAPEE pathogens—*E. faecium, S. aureus, K. pneumoniae, A. baumannii, P. aeruginosa, Enterobacter* spp., and *E. coli*—showing that overall number of reported substances show a slight decrease in the past few years.

## Capital Investment

**Figure 16:**
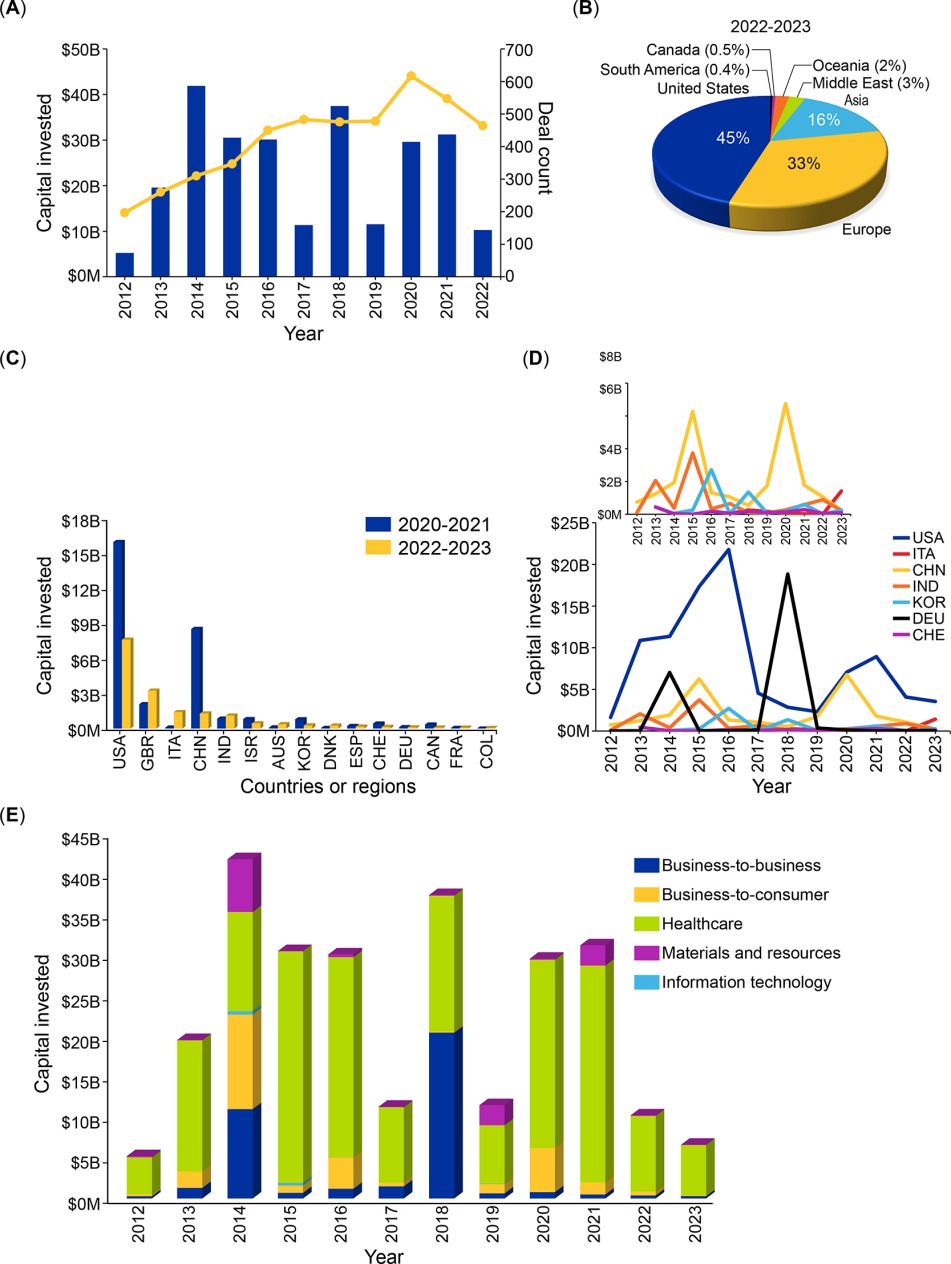
Commercial interest in antibiotics (data from PitchBook). (A) Capital invested and deals related to antibiotics for the past decade (2012 to 2022). (B) Geographical distribution of capital invested in 2022 and 2023 in the field of antibiotics. (C) Leading countries or regions in terms of capital invested over 2020–2023. (D) Growth in capital invested over time for a few key countries or regions. Standard three-letter codes are used to represent countries or regions. (E) Distribution of capital invested across different industry types over the past decade.

Data from Pitchbook,^406^ an online platform for investment data, reveals a steady increase in invested capital over the past decade (Figure 16A). The exceptions appear to be 2017, 2019, and 2022, which show a curious dip in the amount of invested capital (Figure 16A), the exact reason for which remains unspecified. Similar dips, especially around 2016 and 2019, are also observed in our substance data (with a far less noticeable dip in publications) from the CAS Content Collection. In terms of geographical distribution, the US continues to lead in terms of capital invested in 2022 to 2023, followed closely by Europe and Asia (Figure 16B). Among the leading countries or regions, the United Kingdom (GBR) and India (IND) are the only two that show an increase in capital investments from 2022 to 2023 compared with the previous years, 2020 to 2021 (Figure 16C). Despite this, the USA leads in terms of the sheer volume of capital invested, which is ∼5 times that of China (CHN) in 2022 and 2023 (Figure 16C). Growth in capital invested over the past decade for a few of the leading countries or regions indicates a curious periodic trend showcased most notably by the USA, Germany (DEU), and China, and to a smaller extent by India and South Korea (KOR). This trend appears to be characterized by spikes in capital invested between 2013 and 2016 and 2017–2021 (Figure 16D) led by Germany, a country with a strong pharmaceutical research and development initiative/presence/sector. Overall, investments in 2022 and 2023 in the field of antibiotics appear to be lower for most countries or regions except for Italy (ITA) (Figure 16D) and could be a sign of waning interest. In terms of industry type, unsurprisingly, the healthcare sector accounts for most of the capital invested over the past decade (Figure 16E). This is followed by the business-to-business and business-to-consumer sectors. The materials and resources sector also shows a decent volume of investment, which is perhaps indicative of increasing commercial interest (Figure 16E). Finally, the information technology sector accounts for a very small portion of capital invested (Figure 16E).

Overall, the problem of antibiotic resistance is a worldwide concern, especially with the rise of infections from the ESKAPEE list and CDC’s lists of urgent and serious threat bacteria. Trends indicate that funding and research from big pharmaceutical companies have reduced slightly in the past few years because of the failure of various antibiotics and longer timelines required for the development of new antibiotics leading to enormous costs and lesser returns on investment. Various government/nongovernment-funded research institutes have stepped up to fund research in this field, which is evident from the high number of patents by research institutes and newer forms of antibacterials being developed. Trends indicate that in terms of geographical distribution of funding initiatives, the US, Europe, and Asian countries like China, India, etc., lead the market with major focus on the development of newer antibacterials and using futuristic antimicrobial approaches.

## Emerging Antibacterial Approaches

As antibiotic resistance poses a growing threat to global public health, it necessitates innovative approaches that can be used to fight bacterial infections. In the recent years, many such technologies, such as CRISPR, the use of artificial intelligence (AI), bacterial protein degradation (BAC-PROTAC), and antisense oligonucleotides have emerged as powerful approached to combat antibacterial resistance. CRISPR gene editing offers high precision, AI can help with enhancing analytical capabilities of antibacterials, BAC-PROTAC can help in targeted degradation of bacterial proteins, while antisense oligonucleotides can be used to inhibit bacterial gene expression. This section explores each emerging antibacterial approaches and highlights their applications and benefits.

### Role of CRISPR-Based Gene Editing in Antibacterials

Clustered regularly interspaced short palindromic repeats (CRISPR)-based gene editing systems originated in bacteria as a defense mechanism against bacteriophages. However, CRISPR-Cas nucleases, especially CRISPR-Cas9 systems, can be used to produce antimicrobials (Figures 2 and 3).^407^ They are used for designing antibacterial therapies by using engineered CRISPR-Cas systems for gene editing to destroy specific bacterial DNA, thereby offering an alternative for traditional antibiotics. It can be used for “phage therapy enhancement” where bacteriophages can be engineered to offer specific treatment against bacterial infections. CRISPR systems can also be used to understand bacterial pathogenesis and resistance mechanisms, which can help in designing targeted therapies. In addition, they can also be used for developing diagnostic tools, such as specific high-sensitivity enzymatic reporter (SHERLOCK) for rapid and accurate identification of pathogenic bacterial strains. CRISPR-based systems have been used for targeting biofilm formation genes in *P. aeruginosa*.

### Role of AI in Antibacterials

The development of any antibiotic is a tedious and time-intensive process. Low success rates of most candidate drug molecules in combination with lesser returns of investments to companies are major challenges in the field of antibacterial development. The advent of AI has led to an acceleration in drug development with algorithms being developed to identify viable hit molecules. With the rapid advancements in this field, algorithms that are being created using machine learning (ML) and neural networks (NN) are being leveraged for larger in silico exploration and identification of newer antibacterials and combating high AMR rates.^408−411^Figure 3 depicts a clear accelerated growth in journal publications related to the use of AI in antibacterial research in the past decade. However, the increase in the number of patent publications remains relatively low, which indicates nascency in this field and that most research is still in the academic stage and has yet to reach commercialization.

**Figure 17:**
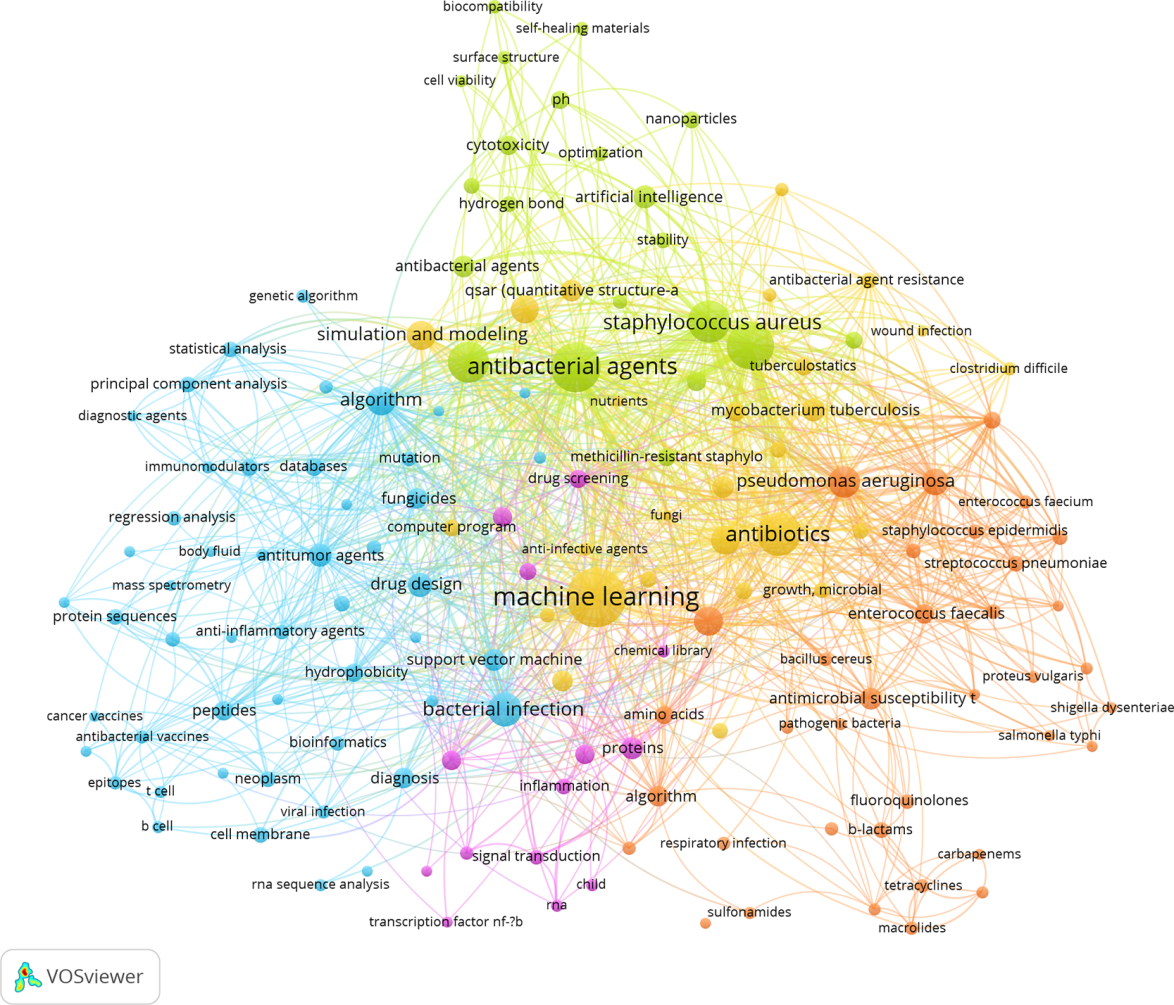
VOSviewer graph indicating networks of top 150 co-occurring concepts related to the use of AI in the field of antibacterials in the past decade.

Figure 17 represents a VOSviewer analysis^412^ for various concepts in the field of AI in antibacterial research. In the network visualization, items are represented by their label and, by default, also by a circle. The size of the label and the circle are directly correlated to the weight of the item. Distance between two items indicates the relatedness of the concepts: the closer the items, the stronger they are related. VOSviewer, by default, also assigns the nodes in a network to clusters (each indicated by a different color). A cluster is a set of closely related nodes. Each node in a network is assigned to exactly one cluster. This figure represents the top 150 co-ocurring concepts that appear more abundantly discussed in academic literature; thus, not all existing concepts on AI in antibacterials are represented in this figure. VOSviewer is also meant to be an interactive image, so some of the information is lost in Figure 13 because of its 2D representation (see the Supporting Information Methods).

The VOSviewer analysis shows that in the past decade, the use of AI in antibacterial research has been carried out to a larger extent for bacteria such as *E. coli, S. aureus, M. tuberculosis*, and *P. aeruginosa*, though research on *K. pneumoniae*, *B. subtilis*, and methicillin-resistant *staphylococcus*, among others, also make an appearance in a much smaller scale. AI-related concept terms, such as “machine learning,” “simulation and modeling,” and “algorithm,” form more and intense connections with various bacteria indicating the increased interest and applicability of AI in this field (Figure 2). This analysis also demonstrates potential areas of growth in this field through, for example, targeting other bacteria apart from the ones previously mentioned; analysis of potential antibacterials in the realm of biocompatibility, cytotoxicity, stability, and metabolites; increasing the use of AI in antibacterial resistance studies, screening, and development of a larger scope of potential antibiotics of different classes; application of AI in nanoparticle and nanomaterial construction, use, and screening as potential antibacterial agents; the application of certain statistical and AI models, such as regression analysis and random forest ML algorithms; and the drastic need for improving the processing of all the information generated by this new technology. Various reviews discussing the use of AI and its limitations in resistance studies and drug discovery have been published.^409,411,413−417^

### Role of Bacterial Protein Degradation Systems (BAC-PROTAC)

Bacterial PROTACs or BAC-PROTACs are an emerging approach to antibacterial therapy. These are chimeric molecules that target specific proteins in bacteria that are no longer needed or are damaged and, in turn, recruit bacterial proteasomal machinery to degrade these proteins.^418^ This system targets a wide range of proteins, and since the mechanism is different from traditional antibiotics, it is less likely to cause resistance. In addition, it is also useful against MDR bacteria that are difficult to kill by traditional antibiotics.^419^ Mechanistically, a bifunctional BAC-PROTAC targets caseinolytic protease complex (ClpC-ClpP) for specific protein degradation by binding to both the target protein and activating ClpC component. In recent studies, scientists have developed BAC-PROTACs targeting *M. tuberculosis*.^420^ This technology is in nascent stages and can be explored further for treating resistant bacterial infections.

### Role of Antisense Oligonucleotides

Antisense oligonucleotides are emerging as new tools in antibacterial research, these are short strands of single-stranded DNA or RNA molecules designed to target a specific bacterial mRNA, thereby preventing target protein synthesis. Most often, proteins involved in vital and housekeeping processes, such as those responsible for bacterial replication and survival, are targeted. Newer methods of delivering antisense oligonucleotides, such as by using nanoparticles and cell penetrating peptides, are being explored.^421,422^

## Perspectives and Future Scope

The global spread of MDR bacteria is an alarming problem causing a threat to human health. The statistics from reputable sources, such as the WHO, CDC, and World Bank, have revealed the severity of the threat that resistant bacteria can cause. They regularly publish reports that provide insights into the impact of resistant bacterial infections on the public health domain. In line with preventing and addressing bacterial infections, the CDC’s lists of urgent threats, serious threats, and watchlist species are periodically updated, thereby suggesting the dynamic nature of challenging and ever-evolving resistance among bacterial species. Various research endeavors are being made toward the development of novel antibiotics, but such developments come with their own challenges. For efficient drug delivery, various factors are needed to be considered, such as understanding the pharmacological properties of antibiotics and optimizing drug formulations. In addition, adequate knowledge is needed for specific targeting of antibiotics at the correct target site. The antibiotics should be enhanced for overcoming drug resistance, enhancing drug stability, and shelf life. Similarly, adequate information is also required for addressing host factors for efficient drug delivery.

Development of novel antibiotics requires a deeper understanding of the host immune system, and individual-level differences in the host immune system are responsible for differential results of the same antibiotic treatment in any population. While traditional antibiotic approaches continue to be utilized for the treatment of bacterial infections, the biggest challenge remains the development and persistence of AMR. Bacteria are either naturally resistant to some antibiotics or they develop antibiotic resistance through gene transfer. The problem is compounded by the fact that the development of AMR in bacterial species is much faster than the pace of development of any novel antibiotic.^27,29^ Moreover, the development of antibiotics is more challenging for Gram-negative bacteria because they have an outer membrane that prevents the entry of various drugs. Another major challenge is the treatment of bacterial infections if the bacteria form biofilms because biofilms prevent the entry of antibiotics and the lowest concentration of antibiotics entering the biofilm can promote the development of AMR.^423^ Therefore, there is a dire need for novel antibacterial materials, such as peptides, bacteriophages, enzymes, biopolymeric materials, and hydrogels that can help mitigate the issues with currently available antibacterial drugs. Another major advancement is the use of AI and ML-based approaches and CRISPR-based gene editing methodologies that have slowly started entering the field of antibiotics, which can significantly reduce the timeline for the development of any new antibiotic. The widespread use of AI is still in the nascent stages and requires more research efforts in the future. A better understanding of resistance in bacteria can help in the development of novel antibiotics and treatment strategies to manage bacterial infection.

## Supporting Information Available

Supplementary Table 1, major antibiotic classes and antibiotics belonging to each class; Supplementary Table 2, list of notable clinical trials focusing on antibacterial and antibiotic strategies/methods/therapies ; Supplementary Table 3, list of notable journal articles in the field of antibacterials published in recent years (2021 onward); Supplementary Table 4, list of notable patents published in the field of antibacterials; and Supplemental Figure 1, distribution of various stages of clinical trials related to antibacterial research (PDF)
