# Supplementary material for: Navigating Antibacterial Frontiers: A Panoramic Exploration of Antibacterial Landscapes, Resistance Mechanisms, and Emerging Therapeutic Strategies
Source: ACS Infect Dis. 2024 May 1;10(5):1483–519. doi: 10.1021/acsinfecdis.4c00115 (PMC11091902; doi:10.1021/acsinfecdis.4c00115)
Supplement: Supplementary file 1 — id4c00115_si_001.pdf [file id4c00115_si_001.pdf]

Supplementary Information for

Navigating Antibacterial Frontiers: A Panoramic Exploration of Antibacterial Landscapes, Resistance Mechanisms, and Emerging Therapeutic Strategies

Krittika Ralhan,[1] Kavita A. Iyer,[1] Leilani Lotti Diaz,[2] Robert Bird,[2] Ankush Maind,[1] and Qiongqiong Angela Zhou[2]*

[1]ACS International India Pvt. Ltd., Pune 411044, India

[2]CAS, A Division of the American Chemical Society, Columbus, Ohio 43210, United States

*Corresponding author, email: qzhou@cas.org

S1 Methods

Data extraction and visualization

To perform data extraction, a search query was developed by subject matter experts (SMEs) to include various terms related to the field of antibacterials such as antibacterial resistance, multidrug resistance, bacterial infections, antimicrobial peptides, antibiofilm, bacterial vaccine, antibacterial material, etc. This query was thoroughly examined, and exclusion criteria were added to include the relevant results. The finalized search query resulted in over 40000 documents, which were extracted from the CAPLUS database. The retrieved dataset included journal articles, patents, conference proceedings, dissertations, and preprints published from January 2012 through December 2022. The data was extracted using the CAS Content Collection[1], a repository of diverse scientific knowledge containing over 59 million records from chemistry, biomedical sciences, material sciences, and other topics, which has been extensively curated by SMEs. The following information was extracted: title–abstract and claims (for patents), CAS indexing and concept approaches (based on full-text content), the year of publication, type of document, CAS section and subsection, number of citations for all extracted documents, name of organizations, names of journals and countries in journal publications and patent activity data (assignee and final destination countries) for patent publications, which includes patents published by 97 patent offices around the world. This data allowed for the determination of the chronological flow of filing initial patent applications within patent families through national patent offices, the World Intellectual Property Organization (WO), and the European Patent Office (EPO), leading eventually to individual patent publication activities within national patent offices.

In addition, substance data for the last decade (2012-2022) was retrieved and included information about role indicators, such as THU for therapeutic use, PAC for pharmacological activity, as well as information about substance classes such as small molecule, protein/peptide sequences, polymers, elements, and alloys, among others to analyze trends of substances with therapeutic potential.

For plotting growth trends over time, finding top concepts, and co-occurrence analysis, data was gathered by searching for terms in the title, abstract, claims (for patent publications), and CAS-indexed terms. Search terms included the use of multiple keywords (which were confirmed by extensive literature search and screened by SMEs) comprising abbreviations, synonyms, and indexed alternative terms, to ensure complete coverage while ensuring low noise levels. The Trend Landscape Map for the number of documents was designed and created using Abode Illustrator. Other data analysis about journal and patent publications and substance data was performed using Tableau and MS Excel. Figures were prepared using a combination of Tableau, Microsoft Excel, Adobe Illustrator, and MS PowerPoint. The illustration for the graphical abstract and Figure 1 was created using www.biorender.org.

VOSViewer was used to generate Figure 17. For access to an interactive version of this figure, please email request to corresponding author.

Supplementary Table 1. Major antibiotic classes and antibiotics belonging to each class.

Name | CAS RN	Structure	Mechanism/Target of antibiotic effect	The most common resistance mechanism in bacteria
Isoniazid | 54-85-3	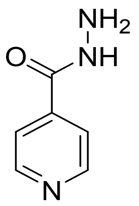	Inhibits synthesis of mycolic acids[2]	Mutation in Isoniazid activating enzyme, Modified drug target (catalase-peroxidase) [3] efflux pumps[4], target overexpression[5]
**Sulfonamides**			
Sulfanilamide | 63-74-1	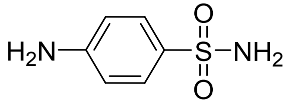	Folic acid synthesis inhibitors[6]	Modified drug target (genetic mutation that leads to modified (Dihydropteroate synthase) DHPS enzyme)[7, 8]
Sulfadiazine | 68-35-9	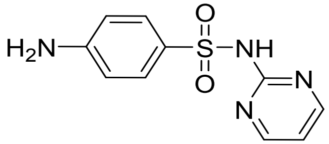		
Sulfapyridine | 144-83-2	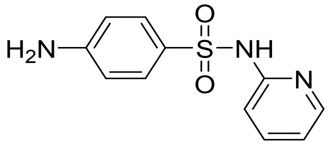		
Sulfasalazine | 599-79-1	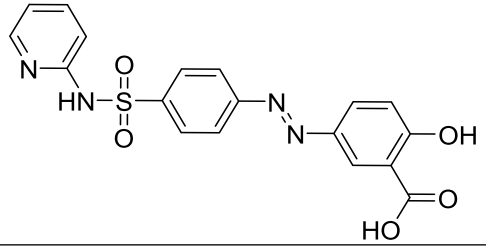		
Sulfamethizole | 144-82-1	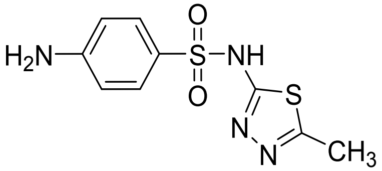		

Sulfacetamide | 144-80-9	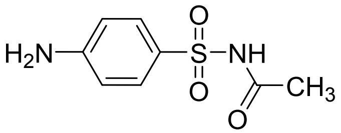		
Sulfamethoxazole | 723-46-6	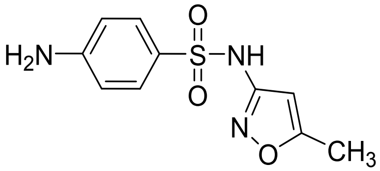		
**β-lactams**			
Penicillin G | 61-33-6	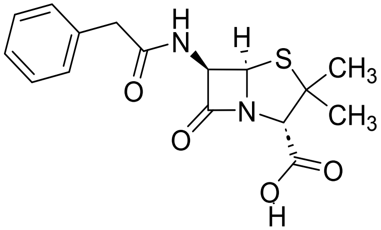	Lyses or weakens cell wall (interferes with transpeptidation process during cell wall formation)[9]	Enzymatic degradation (production of β-lactamases to hydrolyze antibiotics)[10], modified drug target, presence of efflux pump[11] and changes in membrane permeability<br>* Resistance mechanism for Cefilavancin is not clear
Sulbactam | 68373-14-8	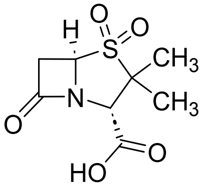	β-lactamase inhibitors[12]	
Clavulanic acid | 58001-44-8	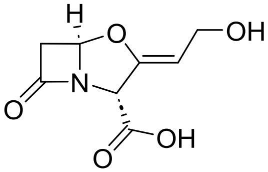	β-lactamase inhibitors[13]	
Tazobactam | 89786-04-9	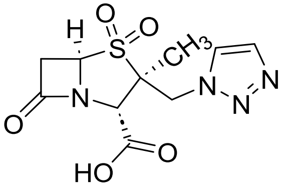	β-lactamase inhibitors (ref)	

Cefilavancin (TD-1792) | 722454-12-8	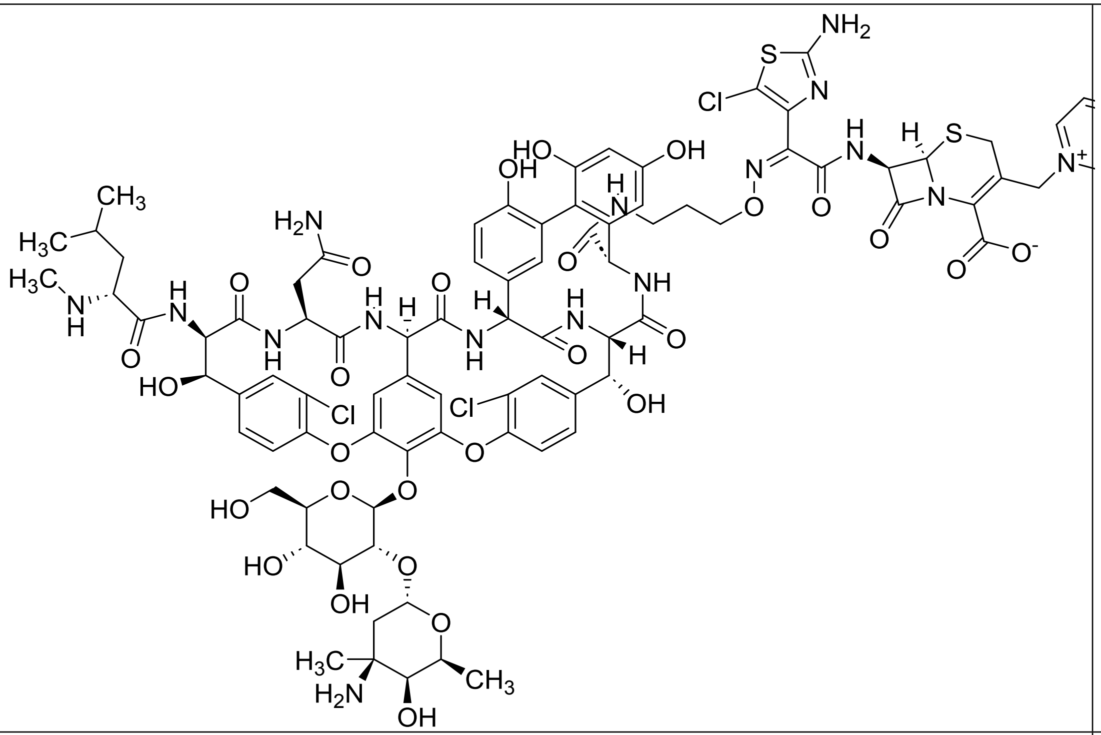	Vancomycin-cephalosporin (β-lactam) heterodimer which acts by inhibiting cell wall synthesis[14]	
Cefiderocol | 1225208-94-5	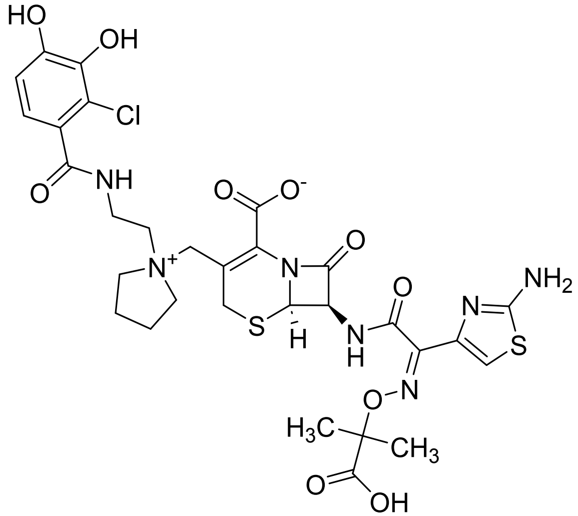	Lyses or weakens cell wall (it enters the cell by utilizing bacterial iron transport system)[15]	

Doripenem | [148016-81-3]	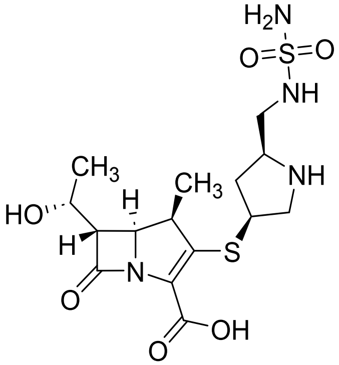	Lyses or weakens cell wall[16]	
Meropenem | [96036-03-2]	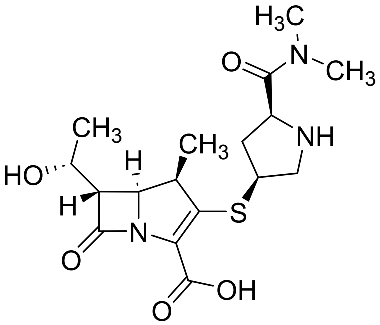	Lyses or weakens cell wall[17]	
Ampicillin | [69-53-4]	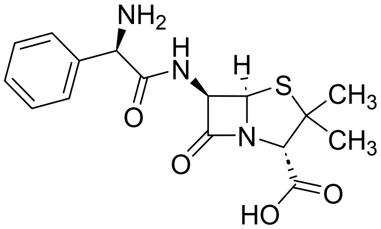	Lyses or weakens cell wall[18]	
**Non-β-lactam β-lactamase inhibitors**			
Avibactam | [1192500-31-4]	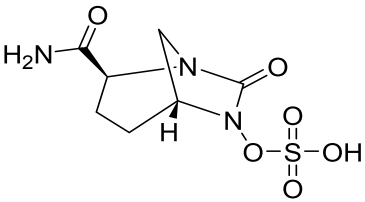	β-lactamase inhibitors (causes covalent acylation of β-lactamase[19]	

Vaborbactam | 1360457-46-0	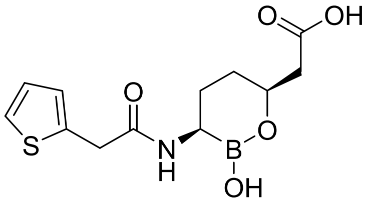	β-lactamase inhibitors[20]	
**Aminoglycosides**			
Streptomycin | 57-92-1	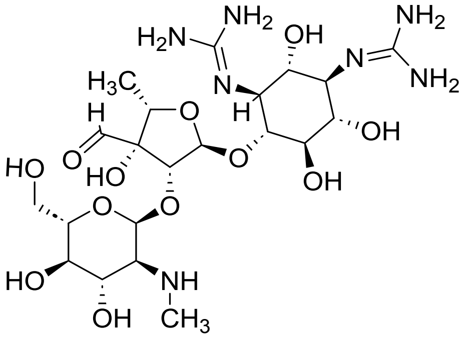	Inhibition of bacterial protein synthesis (bind to the 16S ribosomal RNA at the tRNA acceptor aminoacyl-site (A-site) on the 30S ribosome, interferes with codon-anticodon recognition during translation. They specifically target the proofreading process)[21]	Modified target site (Enzymatic modification by aminoglycoside acyltransferases (AACs), aminoglycoside phosphotransferases (APHs), and aminoglycoside nucleotransferase (ANTs))[22]; target site modification via methylation of 16S rRNA or chromosomal mutation[23]; efflux pumps[24], uptake and permeability mutations; and highly efficient membrane proteases[25]
Kanamycin | 59-01-8	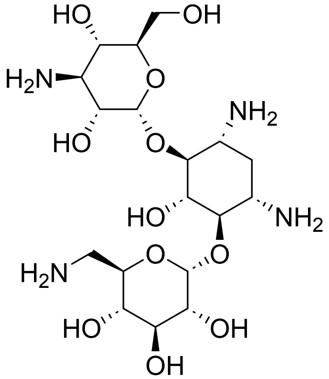		
Gentamicin | 1403-66-3	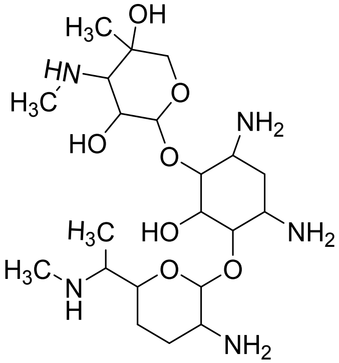		

Sisomicin | 32385-11-8	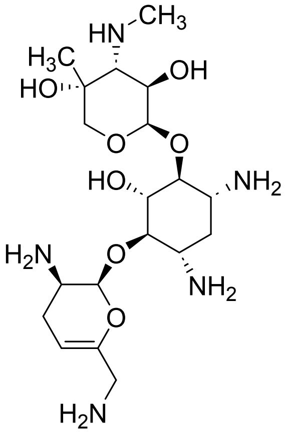		
Amikacin | 37517-28-5	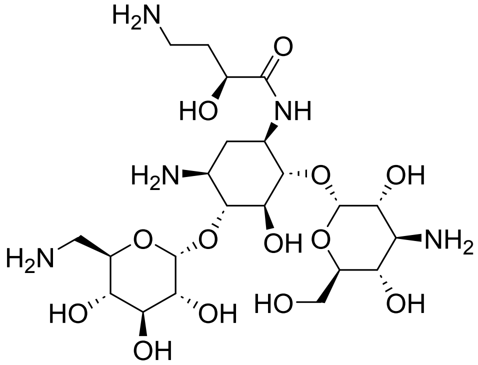		
Netilmicin | 56391-56-1	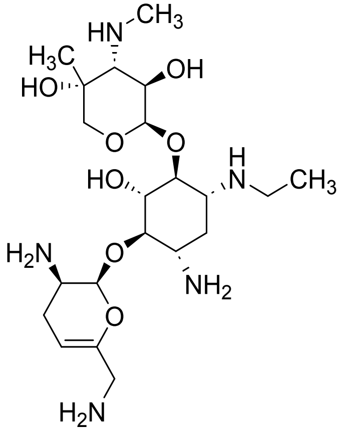		

Arbekacin | 51025-85-5	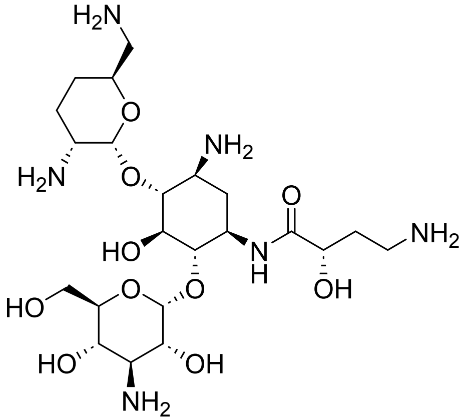		
Plazomicin | 1154757-24-0	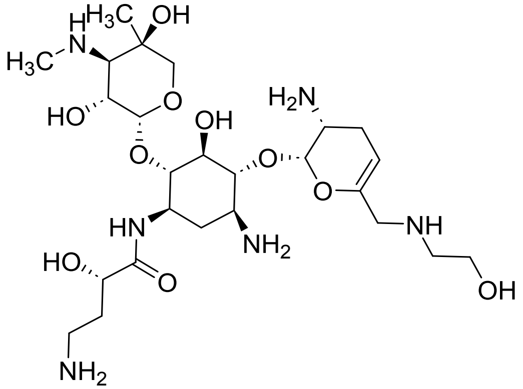		
Apramycin | 37321-09-8	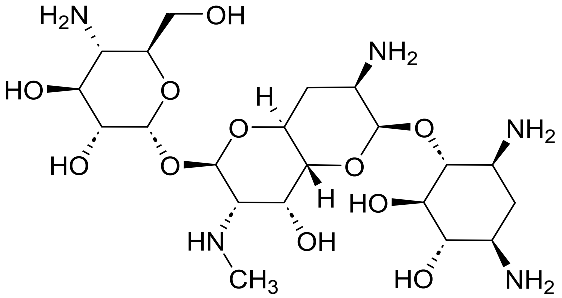		

Neomycin | 1404-04-2	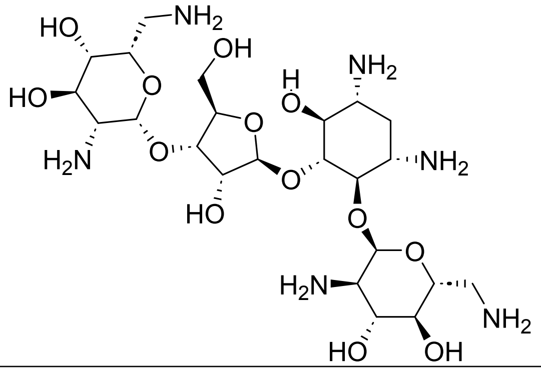		
Ribostamycin | 25546-65-0	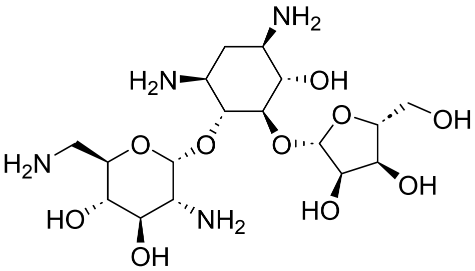		
Tobramycin | 32986-56-4	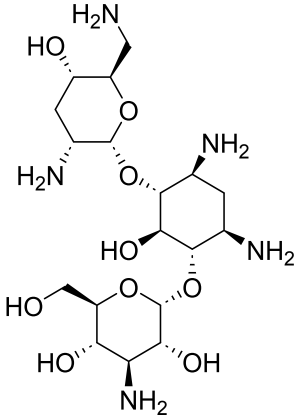		
**Tetracyclines**			
Tetracycline | 60-54-8	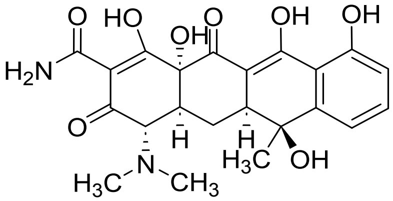	Inhibition of bacterial protein synthesis (bind reversibly to the A site of the 30S ribosomal unit, interfering with the binding of the	

Aureomycin | 57-62-5	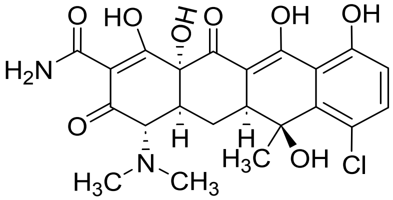	aminoacyl-tRNA to the acceptor site of the mRNA-ribosome complex, thereby preventing the elongation of polypeptide chain)[26]	Efflux pumps, enzymatic degradation/modification of antibiotic disruption its interaction with the 30S subunit by ribosomal protective proteins (TetM and TetO), deactivation via hydroxylation of position C-11a (TetX and Tet 37), modified binding site, and changes in membrane permeability[27, 28]
Terramycin | 79-57-2	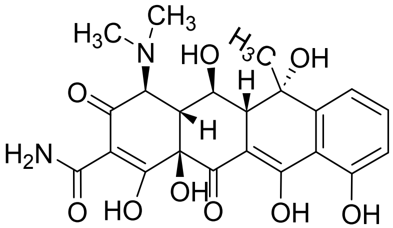		
Demeclocycline | 127-33-3	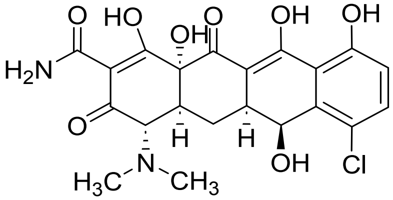		
Lymecycline | 992-21-2	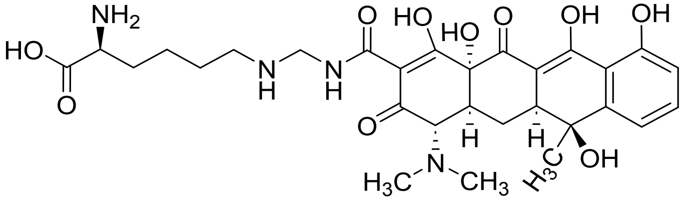		
Methacycline | 914-00-1	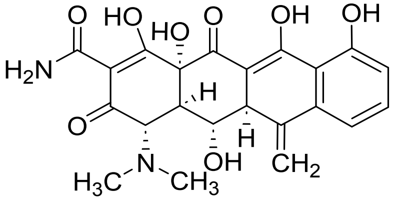		
Minocycline | 10118-90-8	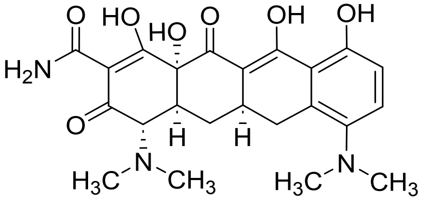		

Rolitetracycline | 751-97-3	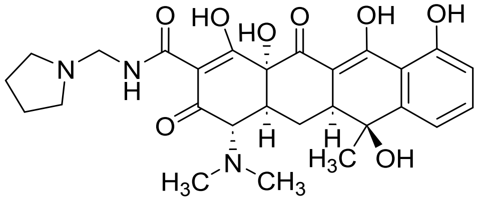		
Sarecycline | 1035654-66-0	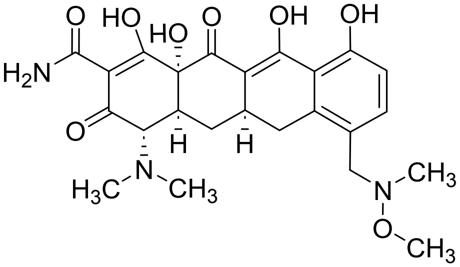		
Omadacycline | 389139-89-3	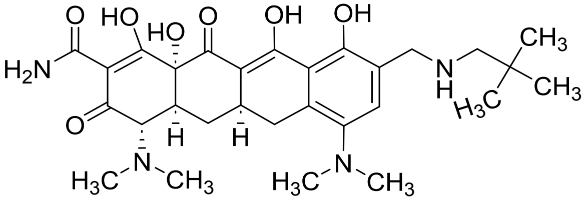		
Doxycycline | 564-25-0	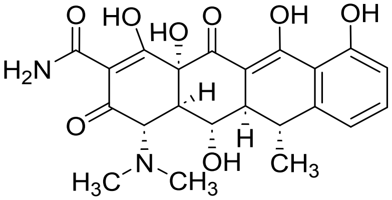		
Tigecycline | 220620-09-7	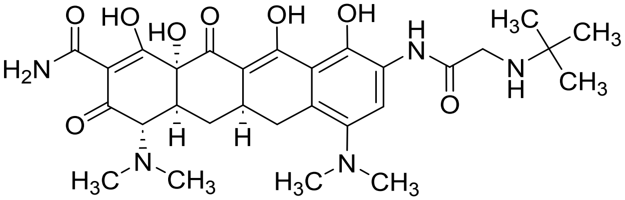		
Eravacycline (TP-271) | 1207283-85-9	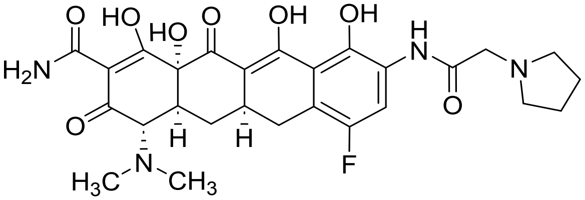		
**Polymyxins**			

Polymyxin B | [1404-26-8]	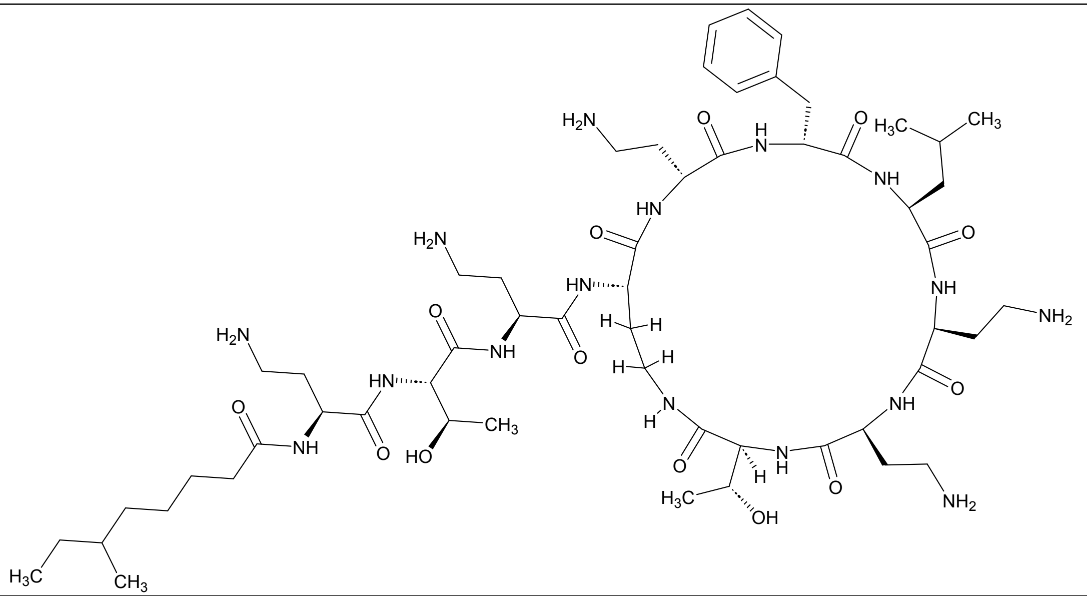	Loss of membrane integrity (generally by binding to lipopolysaccharides (LPS) present in the outer membrane of Gram-negative bacteria; buildup of reactive oxygen species in membranes; inactivate endotoxins.[29] *QPX9003, SPR206 has shown to have a better safety profile and be more effective against lung infections and kidney infections respectively[30, 31]	Efflux pumps, membrane modification to reduce their negative charge, formation of biofilms and capsules, target size modifications[32]
Polymyxin E (Colistin) | 1066-17-7	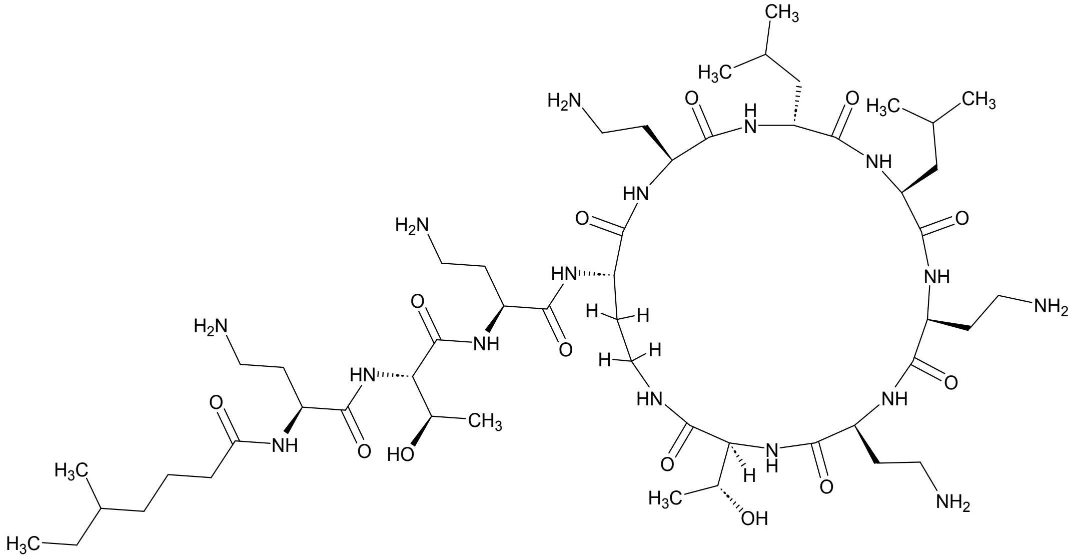		

QPX9003 | 1814918-91-6	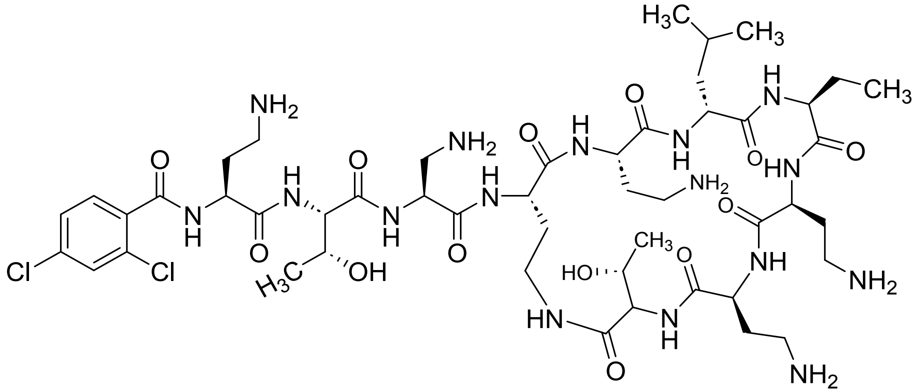		
SPR206 | 2407717-17-1	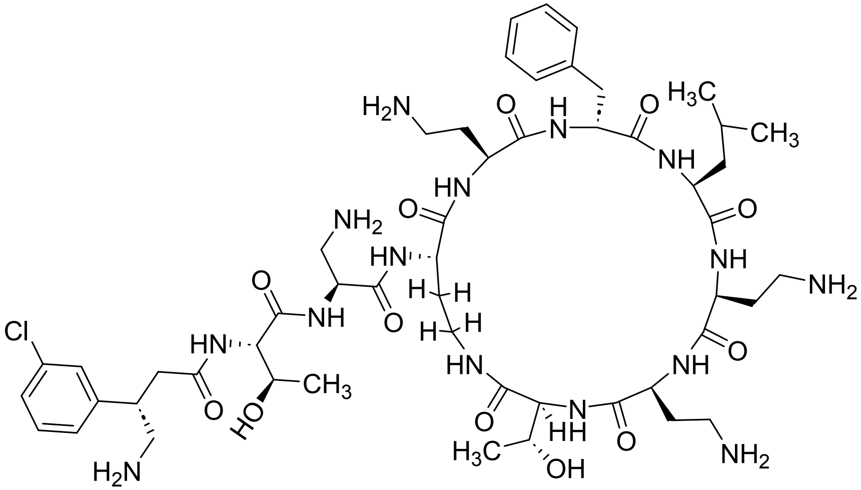		

NAB739 | 1006686-82-3	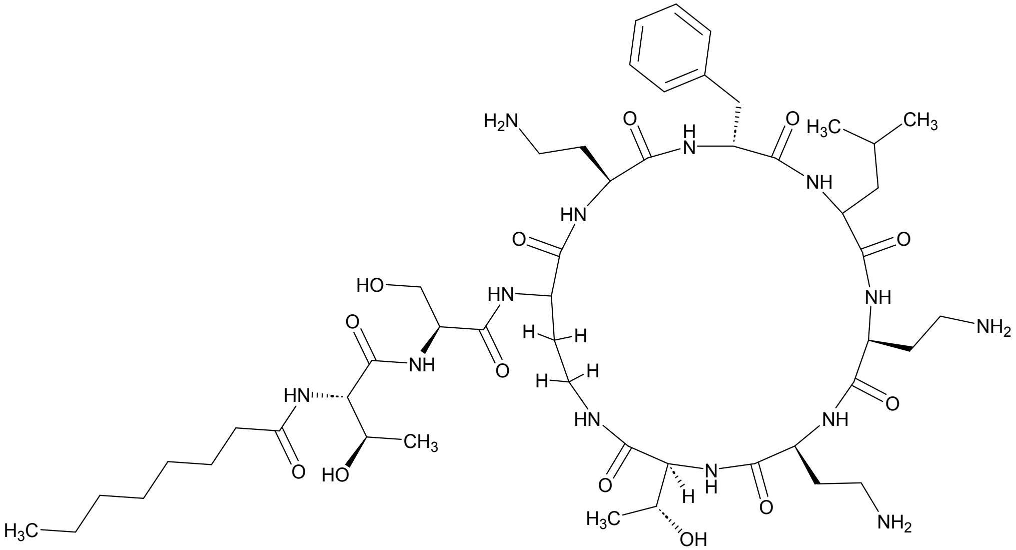		
NAB815 | 1969250-99-4	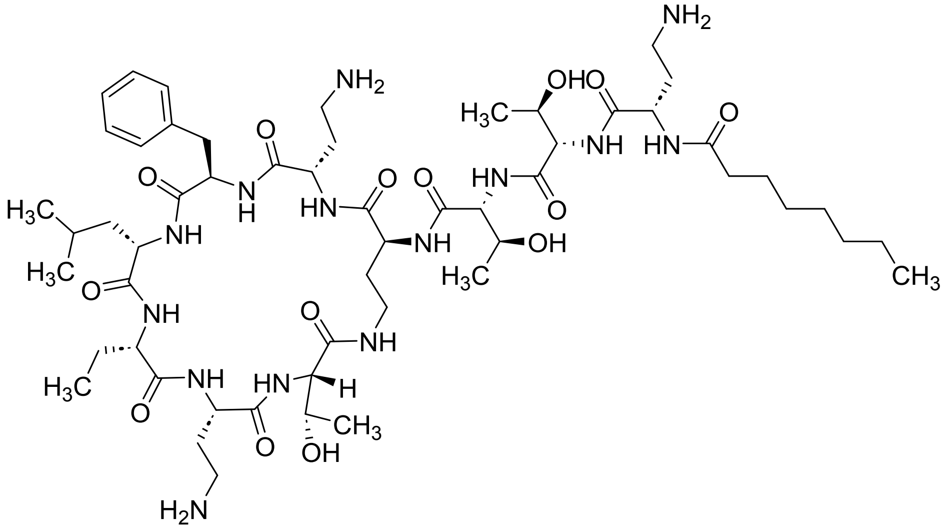		

NAB741 | 1179330-52-9	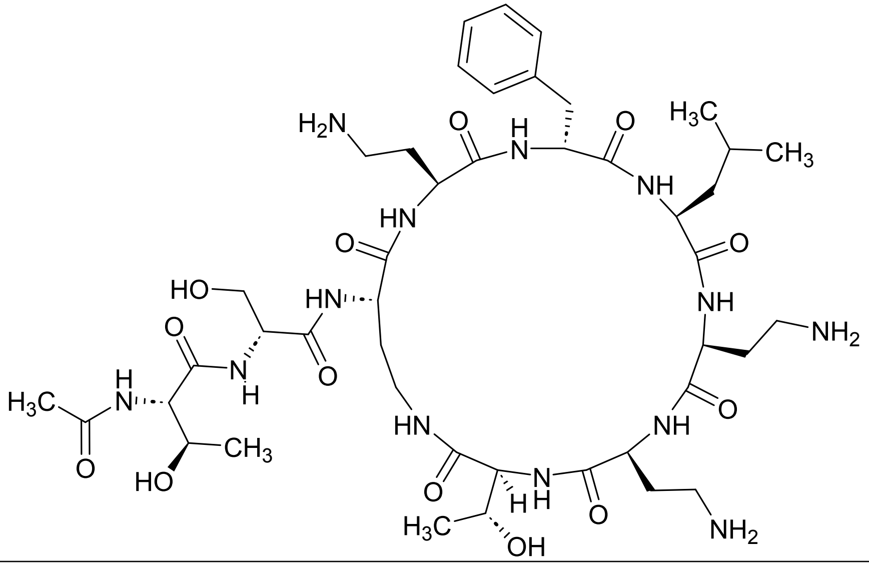		
**Phosphonic antibiotics**			
Fosfomycin | 23155-02-4	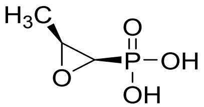	Interferes with the formation of peptidoglycan in the bacterial cell wall (by inhibiting UDP-N-acetylglucosamine-enolpyruvyltransferase)[33]	Target site alteration, enzymatic degradation, reduced uptake, efflux pumps[33]
**Chloramphenicol Analogs**			
Chloramphenicol | 56-75-7	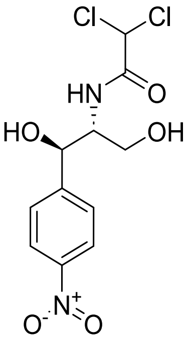	Inhibition of bacterial protein synthesis (bind to 23S rRNA of 50S ribosomal subunit, at the A site of peptidyl transferase center)[34]	Enzymatic degradation (chloramphenicol acetyltransferases (CATs) acetylate chloramphenicol which prevents it from binding ribosomes, drug target modification (due to mutations in 23s

Chloramphenicol succinate | 3544-94-3	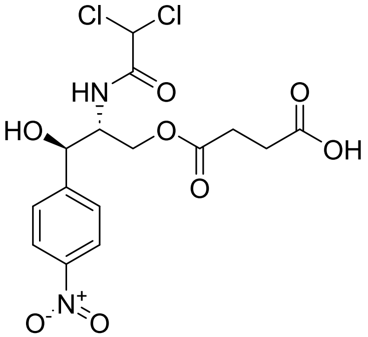		rRNA of 50S ribosomal subunit), efflux pumps, and presence of chloramphenicol efflux proteins (CEPs), changes in membrane permeability.[35, 36]
Thiamphenicol | 15318-45-3	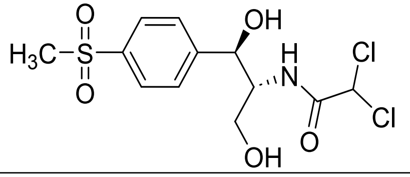		
Florfenicol | 73231-34-2	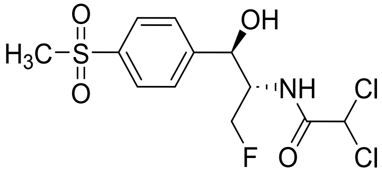		
**Macrolides**			
Erythromycin | 114-07-8	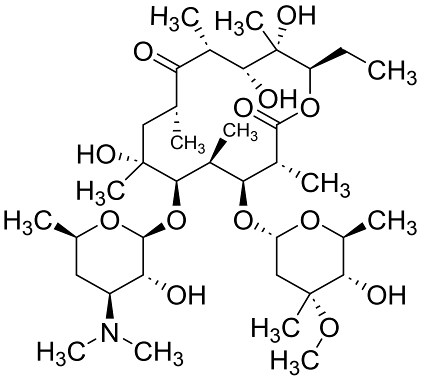	Inhibition of bacterial protein synthesis (bind to 50S ribosomal subunit) disrupting translocation and blocking peptide exit channel[37]	Efflux pumps, modified drug target (mutations in the 23S rRNA sequence), acquisition of a methyltransferase to modify the rRNA, generation of a peptide to displace macrolides from the ribosome, phosphorylation or

Dirithromycin | 62013-04-1	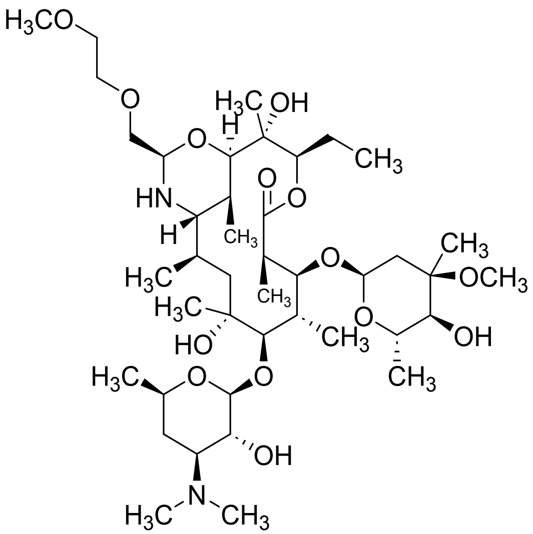		lactone hydrolysis[38]
Clarithromycin | 81103-11-9	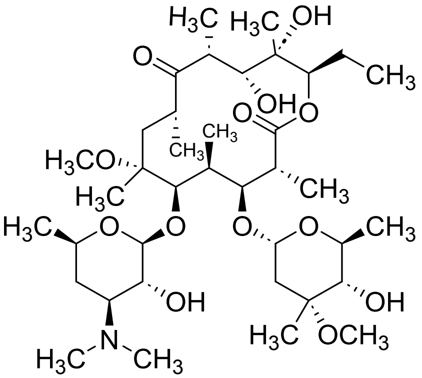		

Azithromycin | 83905-01-5	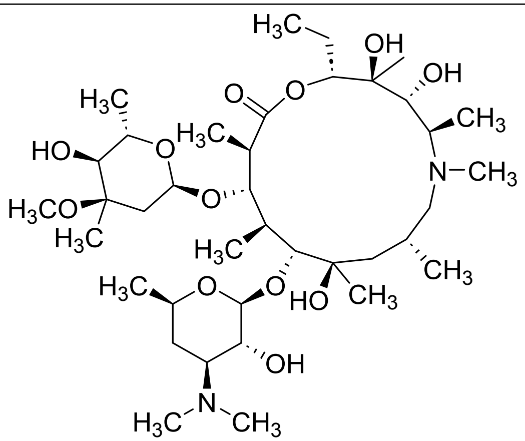		
Fidaxomicin | 873857-62-6	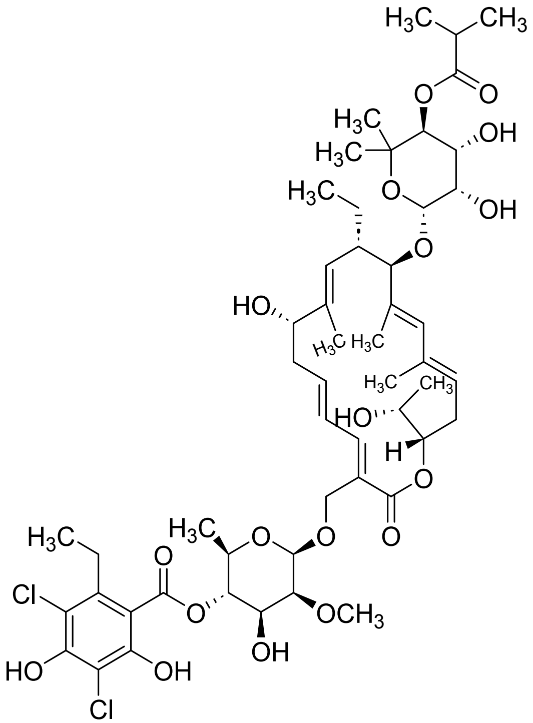		

Telithromycin | [191114-48-4]	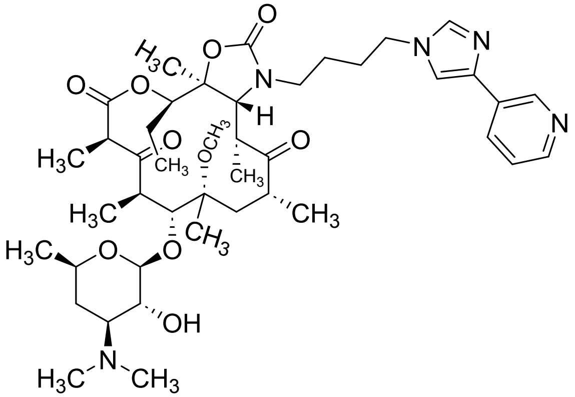		
**Rifamycins**			
Rifampicin | [13292-46-1]	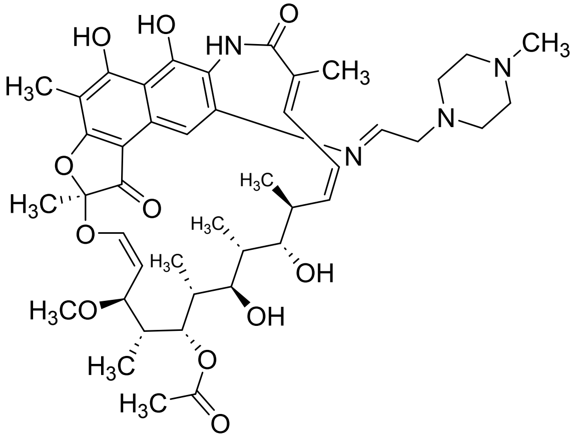	Inhibition of bacterial RNA synthesis (bind to B subunit of RNA polymerases, thus blocking the initiation of RNA synthesis)[39]	Modified drug target (Mutation of rpoB encoding the B subunit of the RNA polymerase)[39]
Rifabutin | [72559-06-9]	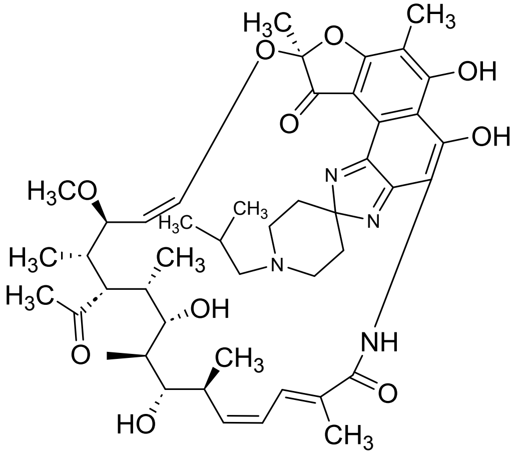		

Rifapentine | 61379-65-5	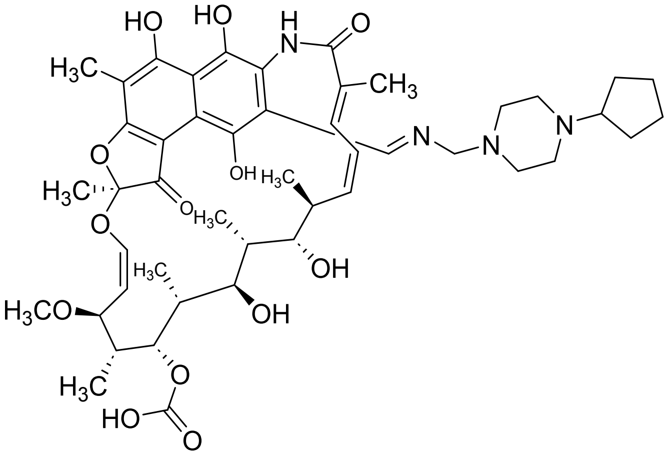		
Rifaximin | 80621-81-4	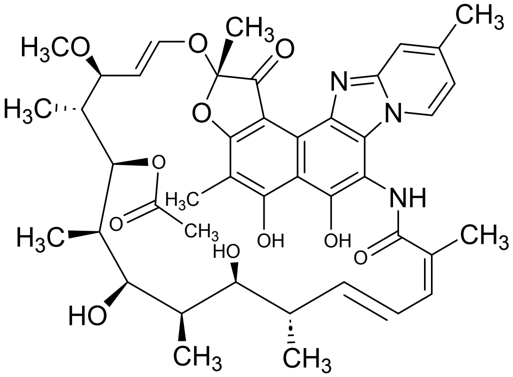		
**Pyrimidines**			
Pyrimethamine | 58-14-0	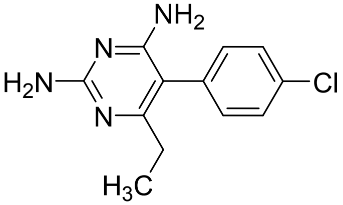	Inhibits dihydrofolate reductase (DHFR)[40]	Modified drug target (mutations in genes encoding dihydropteroate synthetase for sulfonamides and DHFR for trimethoprim), changes in membrane permeability, overproduction of target enzyme[41]
Trimethoprim | 738-70-5	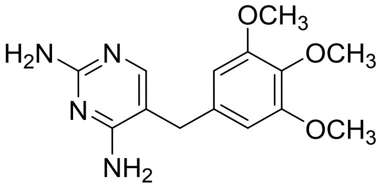		

Iclaprim | 192314-93-5	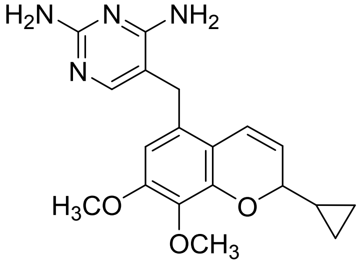		
**Quinolones and Fluoroquinolones**			
Nalidixic acid | 389-08-2	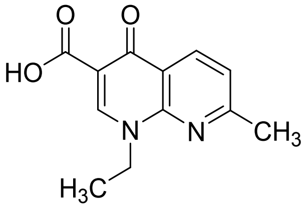	Interferes with DNA synthesis (inhibition of bacterial topoisomerase and DNA gyrase enzymes)[42]	Modified drug targets (mutations in genes encoding DNA gyrase and topoisomerase enzymes), Efflux pumps, Protection of plasmid-mediated proteins (Qnr proteins) that protect DNA gyrase and topoisomerase IV, changes in membrane permeability[42, 43]
Ciprofloxacin | 85721-33-1	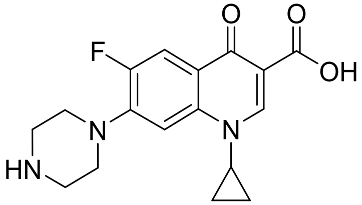		
Gemifloxacin | 175463-14-6	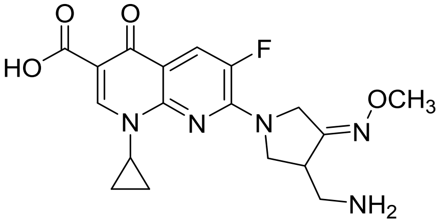		
Levofloxacin | 100986-85-4	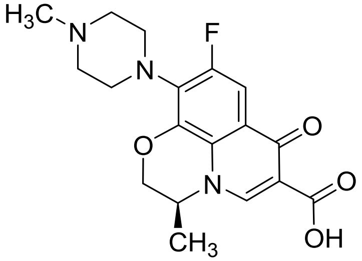		

Moxifloxacin | 151096-09-2	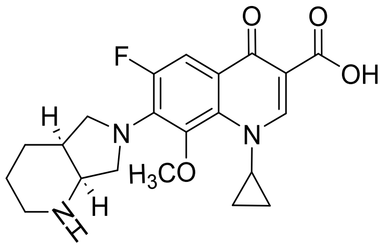		
Norfloxacin | 70458-96-7	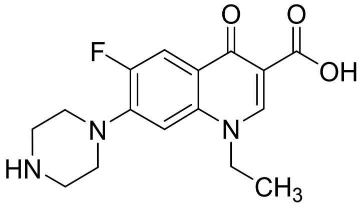		
Ofloxacin | 82419-36-1	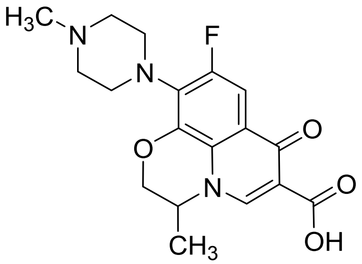		
Sparfloxacin | 110871-86-8	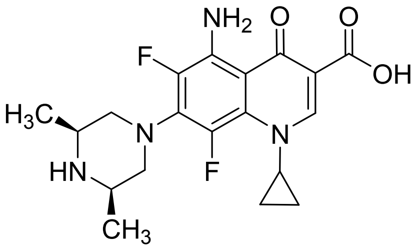		
Delafloxacin | 189279-58-1	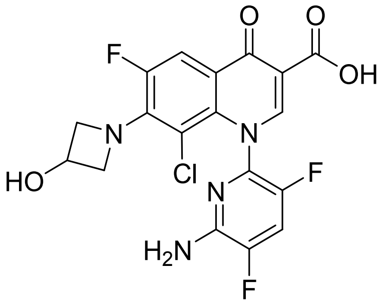		

Trovafloxacin | 147059-72-1	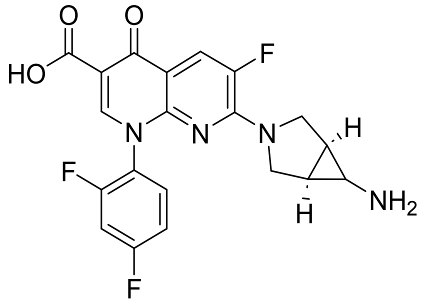		
**Lincosamides**			
Lincomycin | 154-21-2	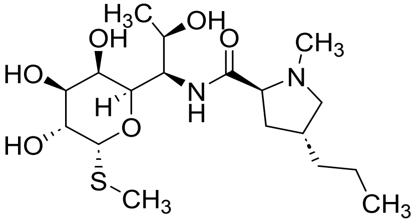	Inhibition of bacterial RNA synthesis (bind to peptidyltransferase center of the 50S ribosomal subunit)[44]	Changes in membrane permeability), in Gram-positive bacteria, reduced passive diffusion in Gram-negative bacteria, efflux pumps, modified drug targets (methylation of the 23S rRNA)[45]
Clindamycin | 18323-44-9	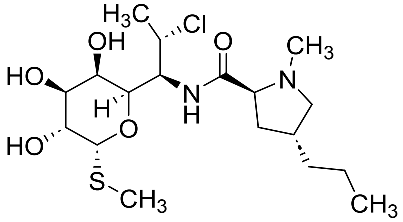		
**Streptogramins**			
Virginiamycin M2 | 21102-49-8	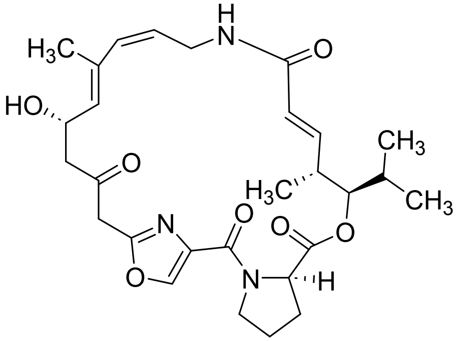	Inhibition of bacterial protein synthesis (bind to 50S ribosomal subunit)[46]	Modified drug target (O-methylation of A2503 within peptidyl transferase center of 23S rRNA in the bacterial ribosome, acetylation of A2503 with virginiamycin acetyltransferases interferes with binding of antibiotics), efflux

Dalfopristin | 112362-50-2	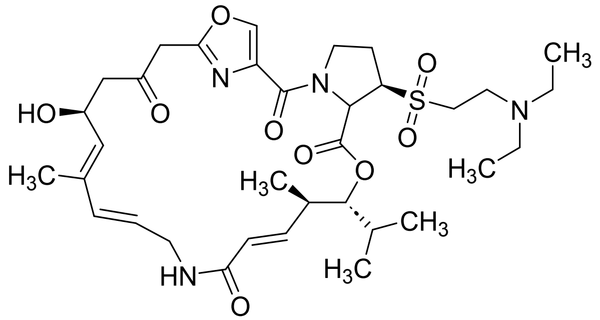		pumps[47, 48]
Quinupristin | 120138-50-3	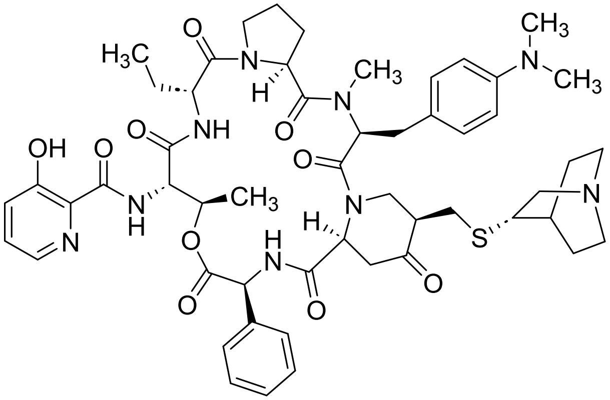	Inhibition of bacterial protein synthesis (50S subunit of 70S ribosome and prevents chain elongation)[49]	
**Oxazolidinones**			
Linezolid | 165800-03-3	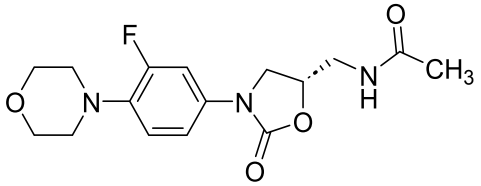	Inhibition of bacterial protein synthesis (bind to 50S ribosomal subunit and preventing the formation of initiation complex)[50]	Modified drug target (O-methylation of A2503 in the 50S subunit of the bacterial ribosome, G2576T mutation in domain V of the 23S rRNA gene), efflux pumps[50]
Tedizolid | 856866-72-3	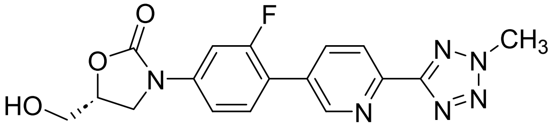		
**Pleuromutilins**			

Pleuromutilin | 125-65-5	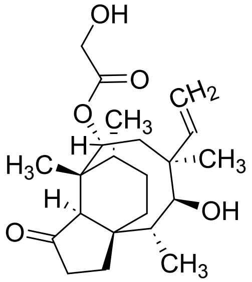		
Tiamulin | 55297-95-5	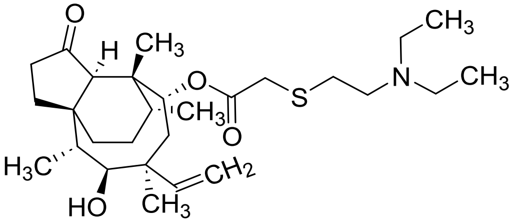		
Valnemulin | 101312-92-9	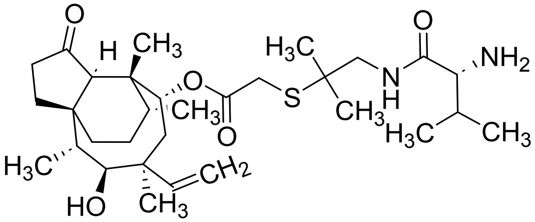	Inhibition of bacterial protein synthesis (bind to 50S ribosomal subunit)[51]	Modified drug target (Methylation of A2503 in rRNA, mutations in the L3 ribosomal protein[51, 52]
Retapamulin | 224452-66-8	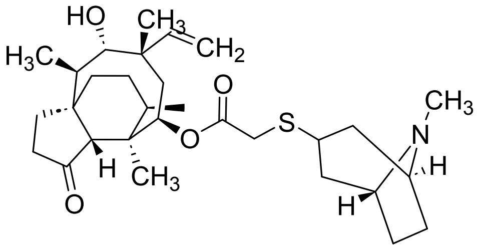		
Lefamulin | 1061337-51-6	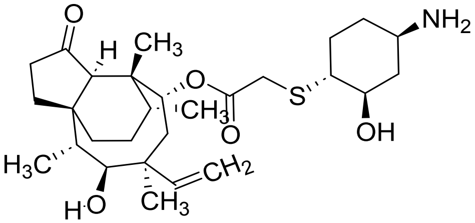		
**Stringent response inhibitors**			

<table>
<tr><td>2’-deoxyguanosine-3’,5’-di(methylene bisphosphonate) | 1187644-84-3</td><td>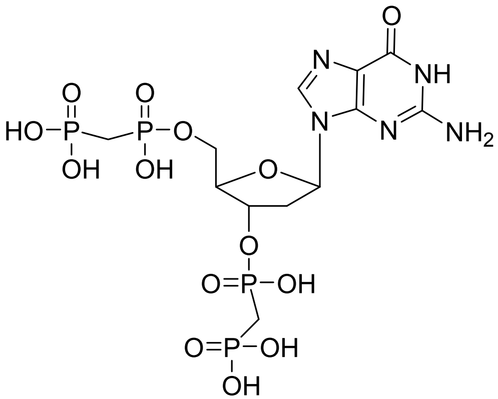</td><td rowspan="2">Decreases production of guanosine 5’-triphosphate 3’-diphosphate</td><td rowspan="2">NA</td></tr>
<tr><td>Relacin | 1357928-14-3</td><td>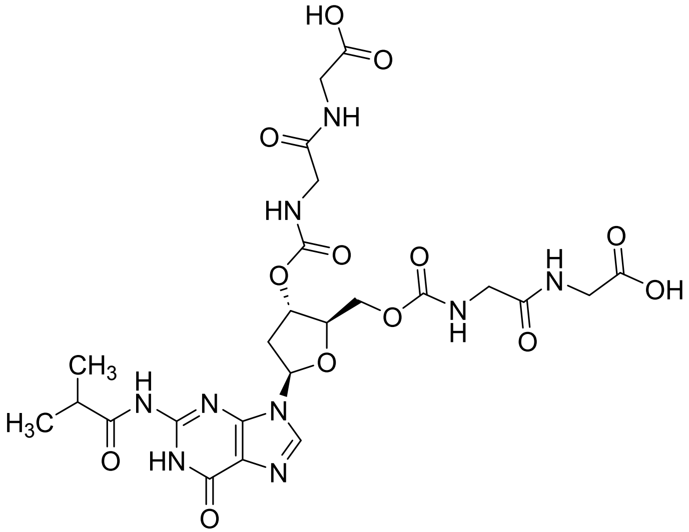</td></tr>
<tr><td colspan="4">Antimicrobial peptides (AMPs)</td></tr>
</table>

<table>
<tr><td>Vancomycin | 1404-90-6</td><td>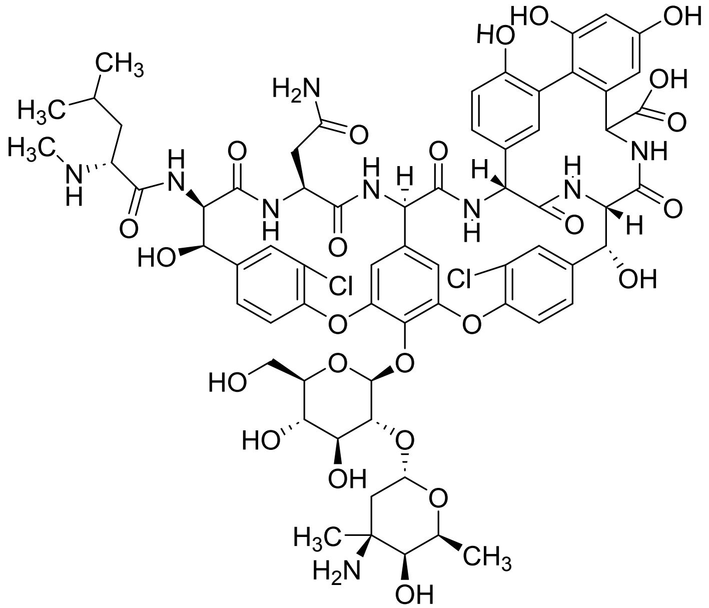</td><td rowspan="2">Cell wall inhibition (binding to the C-terminal D-Ala-D-Ala moiety of the peptidoglycan precursor Lipid II, preventing transglycosylation and transpeptidation for cell wall synthesis)</td><td rowspan="2">Target site modification, cell wall thickening, enzymatic modification of vancomycin and efflux pumps[53]</td></tr>
<tr><td>Teicoplanin | 61036-62-2</td><td>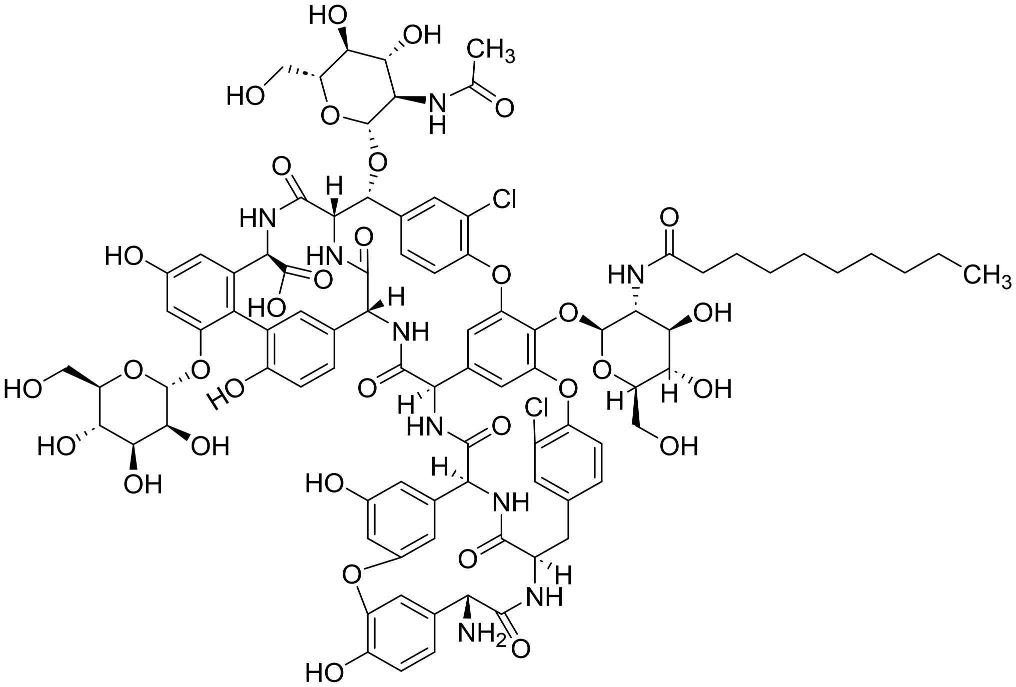</td></tr>
</table>

Daptomycin | 103060-53-3	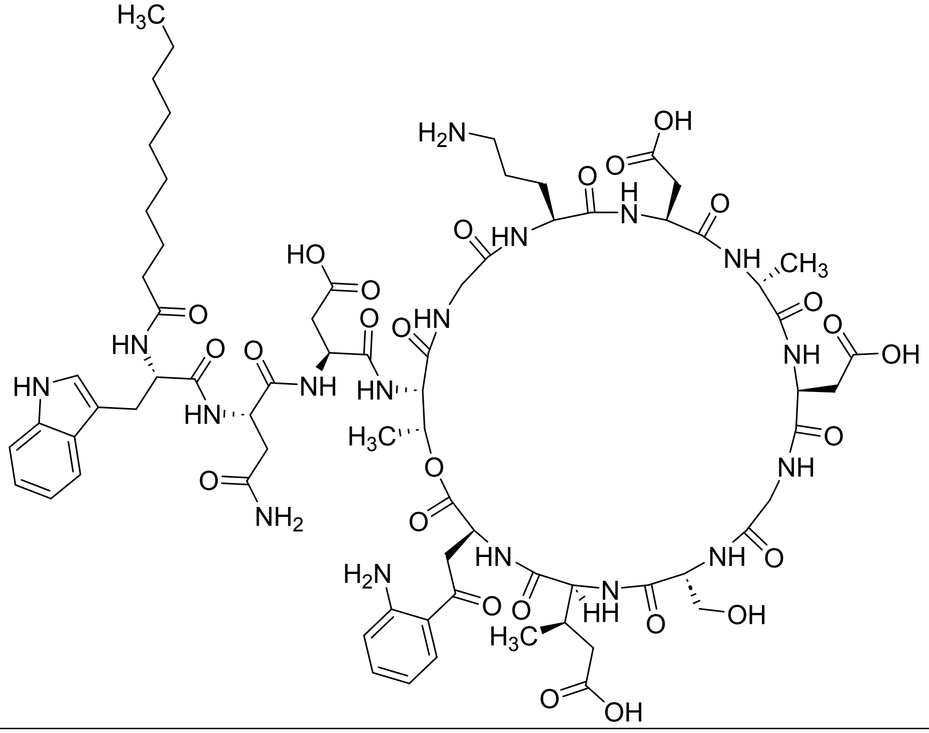	Disrupting the bacterial cell membrane directly by binding to phosphatidylglycerol	Changes in membrane, efflux pumps[54]

EM49 (Octapeptin) | 39342-08-0	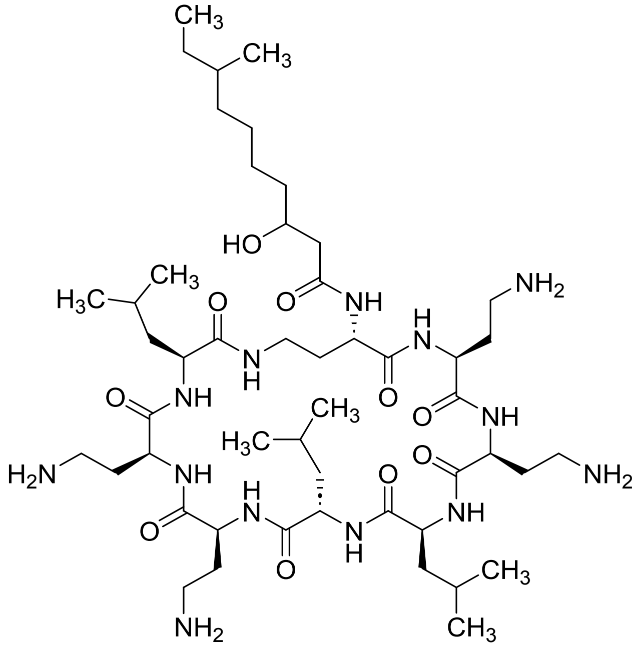	Cell membrane disruption by insertion into the cell membrane	
Friulimicin B | 239802-15-4	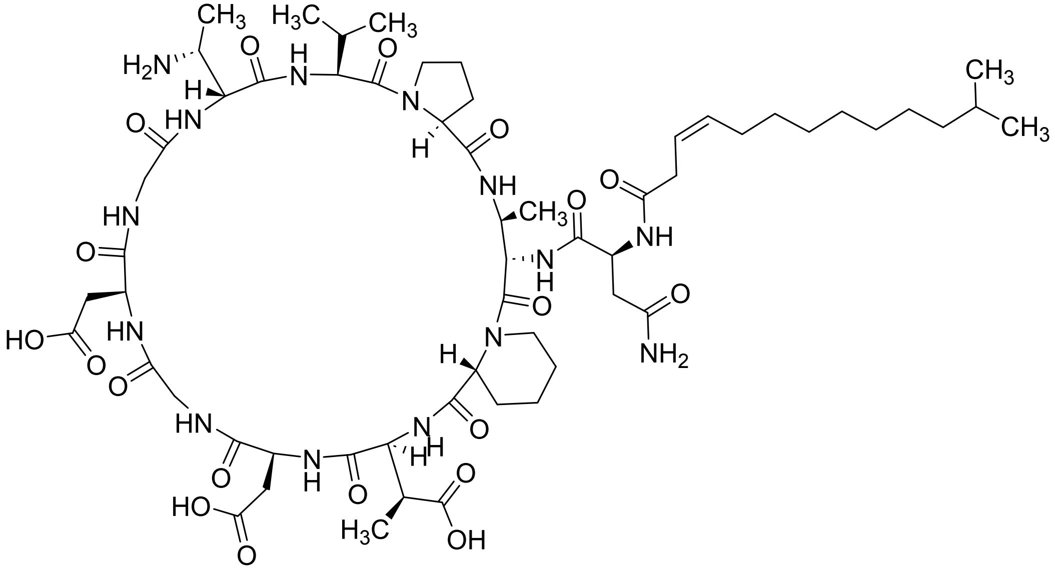	Disrupts membrane integrity (Lipophilic nature helps interact with the lipid bilayer of the cell membrane)[55]	

Amphomycin | 1402-82-0	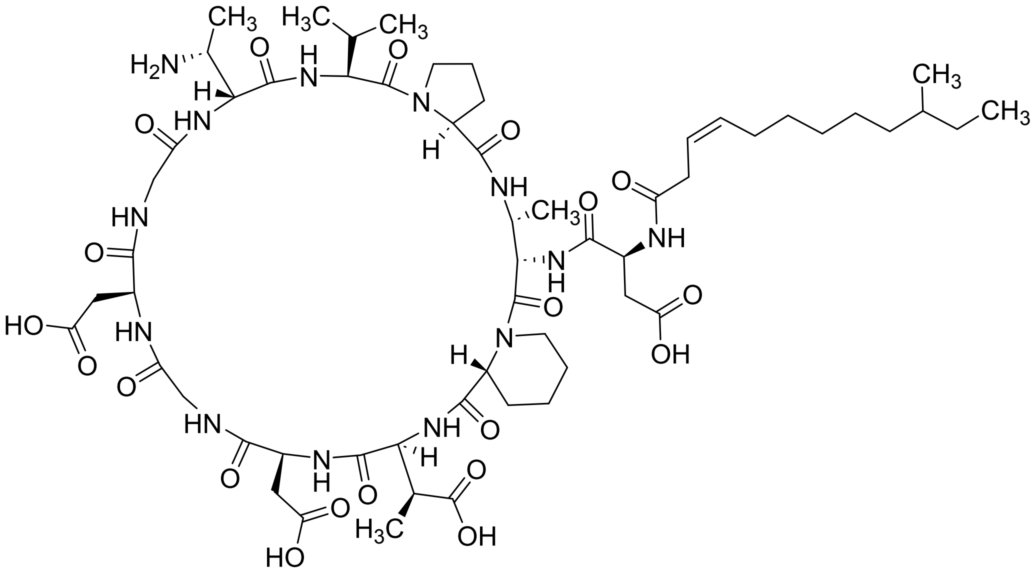	Binds to the cell membrane and forms pores[56]	
Telavancin | 372151-71-8	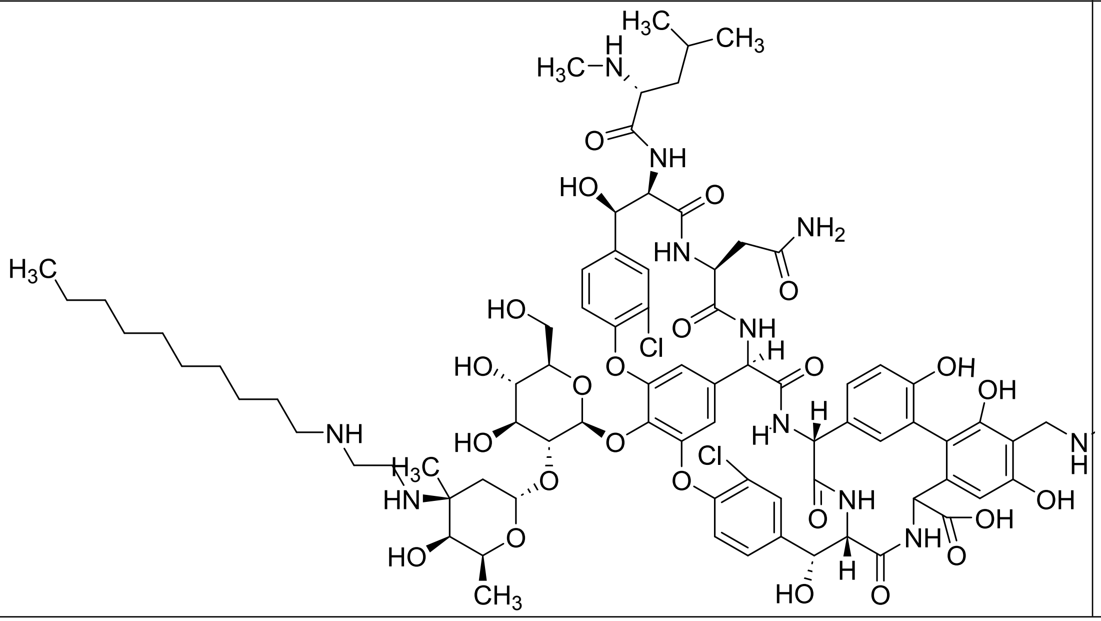	Disrupts cell membrane (by inhibiting transglycosylation and transpeptidation and causing membrane depolarization)[57-59]	

Dalbavancin | [171500-79-1]	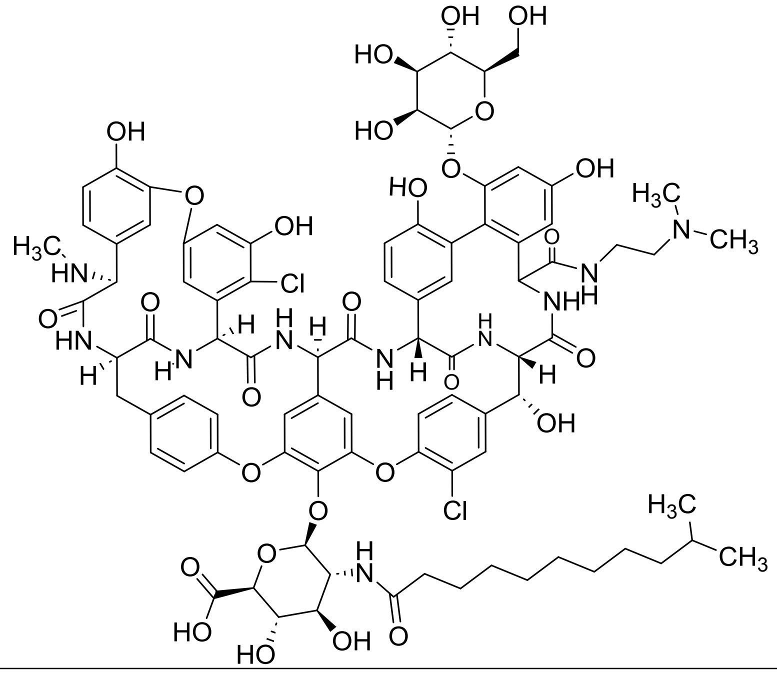		

Oritavancin | 171099-57-3	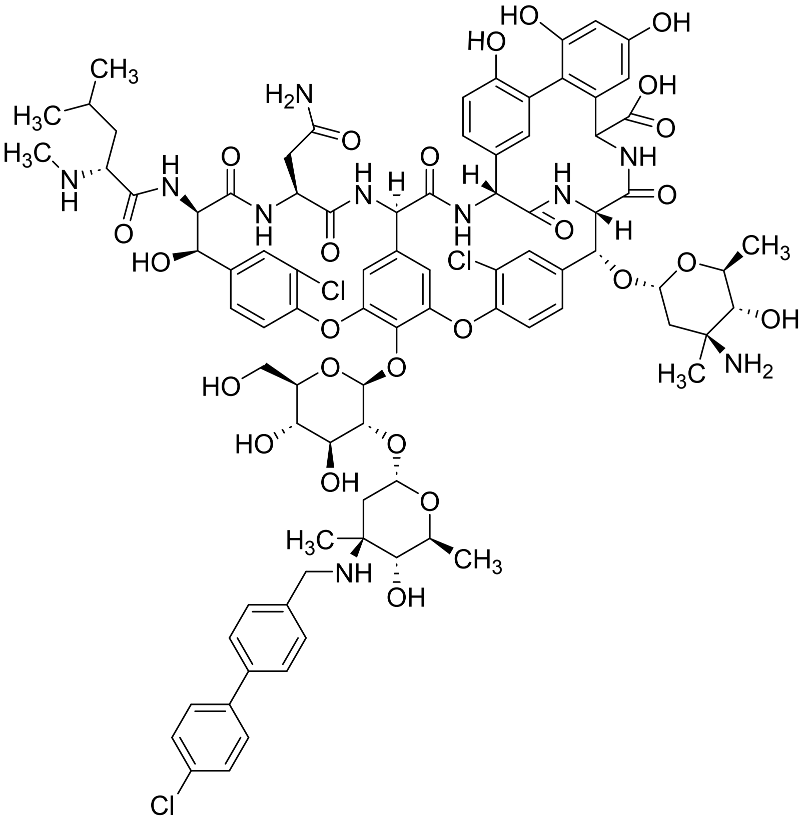		

S3: Clinical Trials

There are more than 8000 clinical trials listed at www.clinicaltrials.gov in the field of antibacterials ranging from different bacterial infections to various treatment strategies being used to tackle bacterial infections. **Supplementary Figure 1** depicts the distribution of clinical trials into various FDA-defined phases where most of the studies are in phase 1 (11%) and phase 2 (18%) of clinical trials. Out of these trials, ~ 3500 focus on antibiotics and antibacterial strategies. A list of a few notable clinical trials has been included in **Supplementary Table 2.**

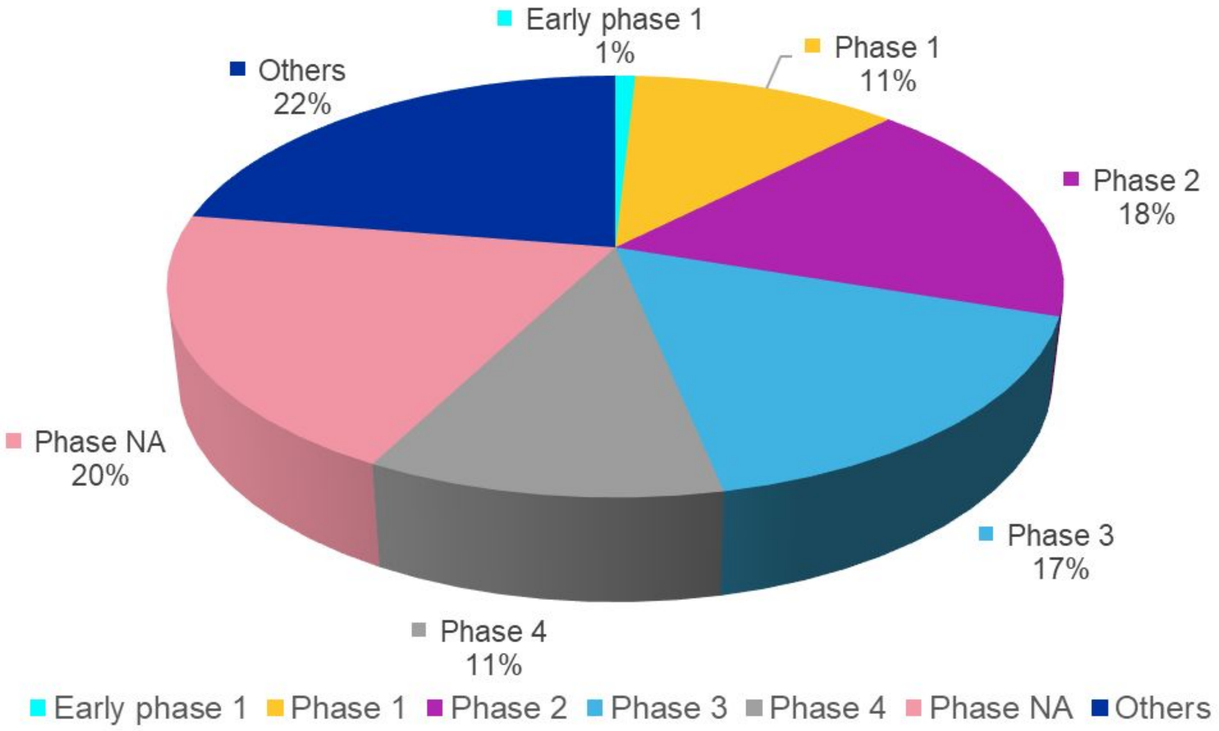

Supplementary Figure 1: Distribution of various stages of clinical trials related to antibacterial research as listed in www.clinicaltrials.gov. Phase NA is defined as clinical trials that do not fit in any FDA-defined phases, including trials of devices or behavioral interventions.

Supplementary Table 2: List of notable clinical trials focusing on antibacterial and antibiotic strategies/methods/therapies as listed in www.clinicaltrials.gov.

NCT Number	Antibacterial strategy/antibiotic/therapy used	Target population
NCT03478150	Antibacterial effect of laser diode and zinc oxide nanoparticles when used as cavity disinfectants	Adults with good oral hygiene
NCT05903339	Antibiotic-loaded hydrogel coating (Defensive Antiadhesive Coating DAC®, Novagenit SRL) that acts as a biofilm inhibitor and prevents peri-prosthetic hip joint infection	Adults with periprosthetic hip joint infection
NCT04256824	Antibacterial effect of triclosan-coated Polyglactin 910 sutures to prevent surgical site infection	Adults who have undergone clean-contaminated wound surgery
NCT04178382	Effect of PCR-CRISPR/Cas12a on the early anti-infective schemes in patients with pneumonia	Adults with suspected pneumonia
NCT03581734	Effect of co-administering oral polio vaccine and whole cell killed oral cholera vaccine	Healthy children (age 1-3 years old)
NCT05017766	Using proteomic, metabolomic, and transcriptomic analysis, immunocytochemical or fluorescence in-situ hybridization (FISH) analysis, flow cytometry analysis (FACS), and immunophenotyping to combat antibiotic-resistant bacteria *E. coli, Klebsiella species, S. aureus, P. aeruginosa*)	Adult patients with confirmed (i) urinary tract infection, (ii) pneumonia (including patients after lung transplantation, cystic fibrosis), or (iii) deep-seated infection
NCT05845073	Efficacy of probiotics on gastrointestinal (GI) complaints and diarrhea in subjects receiving short-term antibiotic (AB) treatment	Healthy adults who have undergone short-term antibiotic treatment (penicillins, cephalosporins, quinolones, tetracyclines, and lincomycins)
NCT04882514	Peptide-based Group A Streptococcal (GAS) Vaccine candidates (J8-K4S2 and p*17-K4S2) in healthy individuals	Healthy adults
NCT05340790	Effect of membrane penetrating antimicrobial peptide: PL-18 vaginal suppositories on vaginal bacteria	Healthy adult females
NCT06156956	Fecal microbiota transplantation (FMT) to decolonize antibiotic-resistant bacteria (ARB)	Children and adult (>6 months old) patients colonized with antibiotic-resistant bacteria,

NCT04682964	Bacteriophage therapy in tonsillitis sing a liquid complex of pyobacteriophages (pyobacteriophage complex liquid - PCL) which causes drug causes lysis of specific bacteria including *Staphylococcus, Enterococcus, Streptococcus, enteropathogenic Escherichia coli, Proteus vulgaris, Proteus mirabilis, Pseudomonas aeruginosa, Klebsiella pneumoniae, and Klebsiella oxytoca*.	Children from 3-14 years with sore throat, acute tonsillitis, and acute respiratory infection (rhinitis, sinusitis, nasopharyngitis)

Supplementary Table 3: List of notable journal articles in the field of antibacterials published in recent years (2021 onwards).

Title	Year	Summary
Synthesis, Characterization, and Antimicrobial Activity of Ultra-Short Cationic β-Peptides[60]	2023	A short cationic noncanonical β,β-dipeptide with a twelve-carbon urea head group was an effective antibiotic and antibiofilm agent against drug-susceptible bacteria and a MRSA and MDR *E. coli* strain. It showed low toxicity in human cells and synergism with other antibiotics.
Tetrachlorovancomycin: Total Synthesis of a Designed Glycopeptide Antibiotic of Reduced Synthetic Complexity [61]	2023	Tetrachlorovancomycin, an analog of vancomycin differing by two chloride substituents, was synthesized in 15 steps. While tetrachlorovancomycin was significantly simpler to prepare than vancomycin, it was only marginally less active than vancomycin, and derivatives with functionality killing bacteria by multiple mechanisms were equipotent to the corresponding vancomycin derivatives.
Divergent Total Synthesis and Characterization of Maxamycins[62]	2023	Maxamycins (vancomycin analogs with replacement of an amide moiety with an amidine and modified substituents) showed extremely high antibacterial activities against vancomycin-resistant bacterial strains and evaded multiple bacterial resistance mechanisms. Tetrachlorovancomycin analogs of maxamycins may yield antibiotics with similar performance but with simpler syntheses.
Targeted Elimination of $bla_{\text{NDM-5}}$ Gene in *Escherichia coli* by Conjugative CRISPR-Cas9 System[63]	2022	CRISPR was effective at inactivating and removing plasmids containing the carbapenem-resistance gene $bla_{\text{NDM-5}}$ in *Escherichia coli* strains.
An engineered live biotherapeutic for the prevention of antibiotic-induced dysbiosis[64]	2022	A bacterial strain with a split beta-lactamase gene (to avoid the advantage of the added strain and to prevent functional acquisition by other bacteria) was created to hydrolyze free antibiotics in the gut and thus minimize its effect on the gut microbiome (and perhaps side effects from antibiotic use). Its use preserved gut microbiome diversity in mice with ampicillin administration didn't reduce serum antibiotic concentrations and inhibited over-colonization of *Clostridium difficile* in the guts of mice.
Epetraborole, a Novel Bacterial Leucyl-tRNA Synthetase Inhibitor, Demonstrates Potent Efficacy and Improves Efficacy of Standard of Care Regimen Against *Mycobacterium avium* complex in a Chronic Mouse Lung Infection Model[65]	2022	The benzodioxobole epetraborole (an inhibitor of leucyl-tRNA synthetase and thus protein synthesis) was prepared and tested by AN2 as an oral agent alone or in combinations with other antibiotics against non-TB mycobacterial strains and was effective.

A naturally inspired antibiotic to target multidrug-resistant pathogens[66]	2022	A natural product antibiotic (macolacin – mutated at 4 positions from colistin) was found by looking for distant colistin/polymyxin analog biosynthetic gene clusters to evade colistin resistance genes and replacement of acyl group with a biphenylcarbonyl group improved activity further.
Bioinformatic prospecting and synthesis of a bifunctional lipopeptide antibiotic that evades resistance[67]	2022	An antibiotic lipopeptide natural product (cliagicin) is found by organizing bacterial sequences by bacterial gene clusters (BGC) and looking for bacteria in groups that don't contain BGC analogous to current antibiotics but in groups that are known to produce antibiotics. A BGC was found that did not match other known BGCs and its product was predicted and synthesized with a common lipid as lipid tail – it was active against all tested Gram-positive bacteria but not Gram-negative bacteria (except *Acinetobacter baumanii*) or human cells. The compound bound C55-PP, preventing transport of intermediates in cell wall synthesis – the target was found because resistance did not develop over time.
A synthetic antibiotic class overcoming bacterial multidrug resistance[68]	2021	The Myers and Polikanov labs used a previously developed synthetic route to clindamycin analogs (https://doi.org/10.1021/jacs.1c03536) to prepare >500 clindamycin analogs with varied acyl and carbohydrate moieties. An analog with an oxepinopyrrolidinecarbonyl moiety (iboxamycin) was prepared and found to have antibacterial activity against a broad set of bacteria, including ESKAPE pathogens and bacteria with known lincosamide resistance genes, and in vivo activity in mice without observed toxicity to human cells. A crystal structure of iboxamycin bound to the methylated 70S ribosome (lincosamide target with methylation to prevent lincosamide toxicity to bacteria) provided evidence of its mechanism and evasion of resistance.
Cationic Lignin-Based Hyperbranched Polymers to Circumvent Drug Resistance in *Pseudomonas* Keratitis[69]	2021	Antibiotic cationic polymers with reduced bacterial resistance were prepared from lignin by acylation followed by ATRP and quaternization. They acted as membrane-disrupting agents – the degree of quaternization did not correlate to activity. While moderate activity was observed, the activity was broad. Use in a rabbit model of keratitis with *Pseudomonas aeruginosa* showed activity slightly lower than that observed for gatifloxacin.

Supplementary Table 4: List of notable patents published in the field of antibacterials.

Title	Patent Number	Description
"DNAzymes targeting cell wall synthesis enzymes and uses thereof"	WO 2023/017501 A1	Cholesterol-substituted DNAzymes are used to bind the RNA for cell wall biosynthetic enzymes such as MurG, preventing their translation and inhibiting cell wall synthesis. Disruption of cell wall biosynthesis is thus used to degrade biofilms or prevent their formation and to kill Gram-negative bacteria.

”Lanthipeptides and methods of use for treating bacterial infections”	WO 2023/150887 A1	Salivaricin 10 – a mixture of phosphorylated lanthipeptide 32mers with three sulfide rings – was isolated from *Streptococcus salivarius* and found to act as an antibacterial agent against Gram-positive and Gram-negative bacteria, including MDR strains, and as anti-inflammatory agents.
“Antibody Hm0283 for Staphylococcus aureus enterotoxin B and application in anti-infective drugs”	CN 116355084 A	The antibody HM0283 for *S. aureu*s enterotoxin B was generated to detect and treat MRSA-related infection and sepsis.
“Quorum sensing inhibitors and composition containing same”	WO 2022/238627 A1	Dienyloxofurancarboxylic acids, esters, and amides, and alkadienyl dihydrofuropyrandiones were isolated from the *Paraconiothyrium variable* and tested as inhibitors of the quorum-sensing pathways in bacteria.
“Cloning of *Aeribacillus pallidus pcynA* genes encoding heat-resistant antibacterial peptide pallidocyclin against Gram-positive microorganisms”	JP 2022-117365 A	The gene for the antibiotic pallidocyclin (structure not disclosed) was sequenced and cloned from bacterial species such as *Aerobacillus pallidus* and kits for its generation are derived. It was tested as an antibacterial agent in Gram-positive bacteria
“Preparation of rhodamine-based AIE photosensitizer for selective killing of gram-positive bacteria and its application of gel dressing thereof”	CN 11489076 A	A hydroxydiiodobenzylidenexanthene rhodamine-based dye was prepared as an aggregation-induced emission-mediated photobactericide and used as a component in an antibacterial wound dressing.
“Rifamycin analogs as antibacterial agents and their preparation, pharmaceutical compositions and use in the treatment of tuberculosis”	WO 2022/159491 A1	Rifamycin analogs (O-aminomethylbenzyl rifalazils) were prepared semisynthetically as potential anti-TB agents that show reduced induction of the human pregnane X receptor and cytochrome P450 2C9 and 3A, reducing drug-drug interactions and side effects that have prevented development of improved rifamycins with reduced resistance profiles.
”Aprosamine derivatives for treatment of bacterial infection”	WO 2022/020295 A1	Oxazolidinone-containing aminoglycoside antibacterials (such as aprosamine derivatives) were prepared semisynthetically as agents effective against aminoglycoside-resistant bacteria.
“Aminocyclitol compounds containing 2-deoxy-*scyllo*-inosamine, their preparation method, and pharmaceutical composition containing same”	KR 10-2022-0093722	2-Deoxy-*scyllo*-inosamines derived from istamycin were prepared as antibiotics against aminoglycoside-resistant strains and for cystic fibrosis.
“Exodeoxyribonuclease VII inhibitors and quinolone antibiotics combination useful for treating bacterial infections”	WO 2022/140631 A1	Isoquinolinediones inhibit exodeoxyribonuclease VII (such as 7-(3-chlorophenyl)-1,3-isoquinolinedione), resensitizing quinolone-resistant bacterial to quinolones; they thus can be used in combination with quinolones as antibacterial agents.
“Detection of heteroresistant populations in monospecies cultures of pathogenic fast-growing bacteria”	RU 2774904 C1	Heteroresistant bacterial populations (in which the highest noninhibitory concentration of an antibiotic is ≥8-fold lower than the lowest concentration causing maximal inhibition – see https://doi.org/10.1128/CMR.00058-14) can be detected by the comparison of the time evolution of optical medium density in the presence and absence of an antibiotic.

"Tuberculostatic composition for inhibiting tubercle bacillus"	CN 112569232 A	The combination of contezolid, bedaquiline, and pretomanid is used as a tuberculostatic agent.
"Bacteriocin produced by *Lactobacillus acidophilus* NM, preparation method and application in preparing broad-spectrum antibacterial drugs"	CN 112175053 A	An antibiotic mixture (bacteriocin) produced by *Lactobacillus acidophilus* NM as a broad-spectrum antibacterial drug with heat stability and limited resistance.

References

1. CAS. *CAS Content*. American Chemical Society, **2023**. https://www.cas.org/about/cas-content (accessed November 3, 2023).
2. Isoniazid. In *Meyler's Side Effects of Drugs (Sixteenth Edition)*, Aronson, J. K. Ed.; Elsevier, **2016**; pp 341-350.
3. Somoskovi, A.; Parsons, L. M.; Salfinger, M. The molecular basis of resistance to isoniazid, rifampin, and pyrazinamide in Mycobacterium tuberculosis. *Respir. Res.* **2001**, *2* (3), 164. DOI: 10.1186/rr54.
4. Narang, A.; Giri, A.; Gupta, S.; Garima, K.; Bose, M.; Varma-Basil, M. Contribution of putative efflux pump genes to isoniazid resistance in clinical isolates of Mycobacterium tuberculosis. *Int. J. Mycobact.* **2017**, *6* (2), 177-183. DOI: 10.4103/ijmy.ijmy_26_17.
5. Vilchèze, C.; Jacobs William, R. Resistance to Isoniazid and Ethionamide in Mycobacterium tuberculosis: Genes, Mutations, and Causalities. *Microbiol. Spectrum* **2014**, *2* (4), 10.1128/microbiolspec.mgm1122-0014-2013. DOI: 10.1128/microbiolspec.mgm2-0014-2013.
6. National Center for Biotechnology Information. *PubChem Compound Summary for CID 5333, Sulfanilamide*. PubChem, **2024**. https://pubchem.ncbi.nlm.nih.gov/compound/Sulfanilamide (accessed January 3, 2024).
7. Eliopoulos, G. M.; Huovinen, P. Resistance to Trimethoprim-Sulfamethoxazole. *Clin. Infect. Dis.* **2001**, *32* (11), 1608-1614. DOI: 10.1086/320532.
8. Pavelquesi, S. L.; de Oliveira Ferreira, A. C.; Rodrigues, A. R.; de Souza Silva, C. M.; Orsi, D. C.; da Silva, I. C. Presence of Tetracycline and Sulfonamide Resistance Genes in Salmonella spp.: Literature Review. *Antibiotics* **2021**, *10* (11). DOI: 10.3390/antibiotics10111314.
9. Gartlan, W. A.; Rahman, S.; Reti., K. Benzathine Penicillin. In *StatPearls [Internet]*; StatPearls Publishing, **2023**, https://www.ncbi.nlm.nih.gov/books/NBK507723/.
10. Dever, L. A.; Dermody, T. S. Mechanisms of Bacterial Resistance to Antibiotics. *Arch. Intern. Med.* **1991**, *151* (5), 886-895. DOI: 10.1001/archinte.1991.00400050040010.
11. Chiu, C.-H.; Lee, H.-Y.; Tseng, L.-Y.; Chen, C.-L.; Chia, J.-H.; Su, L.-H.; Liu, S.-Y. Mechanisms of resistance to ciprofloxacin, ampicillin/sulbactam and imipenem in Acinetobacter baumannii clinical isolates in Taiwan. *Int. J. Antimicrob. Agents* **2010**, *35* (4), 382-386. DOI: 10.1016/j.ijantimicag.2009.12.009.
12. Peechakara, B. V.; Gupta., M. Ampicillin/Sulbactam. In *StatPearls [Internet]*; StatPearls Publishing, **2022**, https://www.ncbi.nlm.nih.gov/books/NBK526117/.
13. Uto, L. R.; Gerriets., V. Clavulanic Acid. In *StatPearls [Internet]*; StatPearls Publishing, **2023**, https://www.ncbi.nlm.nih.gov/books/NBK545273/.
14. Adis International Ltd. *Cefilavancin - Theravance Biopharma*. Springer Nature Switzerland AG, **2023**. https://adisinsight.springer.com/drugs/800020586 (accessed January 4, 2023).
15. Asrat, H.; Samaroo-Campbell, J.; Ata, S.; Quale, J. Contribution of Iron-Transport Systems and ß-Lactamases to Cefiderocol Resistance in Clinical Isolates of Acinetobacter baumannii Endemic to New York City. *Antimicrob. Agents Chemother.* **2023**, *67* (6), e00234-00223. DOI: 10.1128/aac.00234-23.
16. Hilas, O.; Ezzo, D. C.; Jodlowski, T. Z. Doripenem (doribax), a new carbapenem antibacterial agent. *Pharm. Ther.* **2008**, *33* (3), 134-180.
17. Baldwin, C. M.; Lyseng-Williamson, K. A.; Keam, S. J. Meropenem. *Drugs* **2008**, *68* (6), 803-838. DOI: 10.2165/00003495-200868060-00006.
18. Peechakara, B. V.; Basit, H.; Gupta., M. Ampicillin. In *StatPearls [Internet]*; StatPearls Publishing, **2023**, https://www.ncbi.nlm.nih.gov/books/NBK519569/.
19. Lahiri, S. D.; Johnstone, M. R.; Ross, P. L.; McLaughlin, R. E.; Olivier, N. B.; Alm, R. A. Avibactam and class C β-lactamases: mechanism of inhibition, conservation of the binding pocket, and implications for resistance. *Antimicrob. Agents Chemother.* **2014**, *58* (10), 5704-5713. DOI: 10.1128/aac.03057-14.
20. Queenan, A. M.; Bush, K. Carbapenemases: the Versatile β-Lactamases. *Clin. Microbiol. Rev.* **2007**, *20* (3), 440-458. DOI: 10.1128/cmr.00001-07.
21. Ramirez, M. S.; Tolmasky, M. E. Aminoglycoside modifying enzymes. *Drug Resistance Updates* **2010**, *13* (6), 151-171. DOI: 10.1016/j.drup.2010.08.003.
22. Krause, K. M.; Serio, A. W.; Kane, T. R.; Connolly, L. E. Aminoglycosides: An Overview. *Cold Spring Harb. Perspect. Med.* **2016**, *6* (6). DOI: 10.1101/cshperspect.a027029.
23. Doi, Y.; Arakawa, Y. 16S Ribosomal RNA Methylation: Emerging Resistance Mechanism against Aminoglycosides. *Clin. Infect. Dis.* **2007**, *45* (1), 88-94. DOI: 10.1086/518605.

24. Rosenberg, E. Y.; Ma, D.; Nikaido, H. AcrD of *Escherichia coli* Is an Aminoglycoside Efflux Pump. *J. Bacteriol.* **2000**, *182* (6), 1754-1756. DOI: 10.1128/jb.182.6.1754-1756.2000.
25. Garneau-Tsodikova, S.; Labby, K. J. Mechanisms of resistance to aminoglycoside antibiotics: overview and perspectives. *MedChemComm* **2016**, *7* (1), 11-27, 10.1039/C5MD00344J. DOI: 10.1039/C5MD00344J.
26. Chopra, I.; Roberts, M. Tetracycline Antibiotics: Mode of Action, Applications, Molecular Biology, and Epidemiology of Bacterial Resistance. *Microbiol. Mol. Biol. Rev.* **2001**, *65* (2), 232-260. DOI: 10.1128/mmbr.65.2.232-260.2001.
27. Chopra, I.; Howe, T. G. Bacterial resistance to the tetracyclines. *Microbiol. Rev.* **1978**, *42* (4), 707-724. DOI: 10.1128/mr.42.4.707-724.1978.
28. Schmitz, F.-J.; Krey, A.; Sadurski, R.; Verhoef, J.; Milatovic, D.; Fluit, A. C. Resistance to tetracycline and distribution of tetracycline resistance genes in European Staphylococcus aureus isolates. *J. Antimicrob. Chemother.* **2001**, *47* (2), 239-240. DOI: 10.1093/jac/47.2.239.
29. Ayoub Moubareck, C. Polymyxins and Bacterial Membranes: A Review of Antibacterial Activity and Mechanisms of Resistance. *Membranes* **2020**, *10* (8), 181. DOI: 10.3390/membranes10080181.
30. Roberts, K. D.; Zhu, Y.; Azad, M. A. K.; Han, M.-L.; Wang, J.; Wang, L.; Yu, H. H.; Horne, A. S.; Pinson, J.-A.; Rudd, D.; Voelcker, N. H.; Patil, N. A.; Zhao, J.; Jiang, X.; Lu, J.; Chen, K.; Lomovskaya, O.; Hecker, S. J.; Thompson, P. E.; Nation, R. L.; Dudley, M. N.; Griffith, D. C.; Velkov, T.; Li, J. A synthetic lipopeptide targeting top-priority multidrug-resistant Gram-negative pathogens. *Nat. Commun.* **2022**, *13* (1), 1625. DOI: 10.1038/s41467-022-29234-3.
31. Brown, P.; Abbott, E.; Abdulle, O.; Boakes, S.; Coleman, S.; Divall, N.; Duperchy, E.; Moss, S.; Rivers, D.; Simonovic, M.; Singh, J.; Stanway, S.; Wilson, A.; Dawson, M. J. Design of Next Generation Polymyxins with Lower Toxicity: The Discovery of SPR206. *ACS Infect. Dis.* **2019**, *5* (10), 1645-1656. DOI: 10.1021/acsinfecdis.9b00217.
32. Olaitan, A. O.; Morand, S.; Rolain, J.-M. Mechanisms of polymyxin resistance: acquired and intrinsic resistance in bacteria. *Front. Microbiol.* **2014**, *5*, Review. DOI: 10.3389/fmicb.2014.00643.
33. Silver, L. L. Fosfomycin: Mechanism and Resistance. *Cold Spring Harb. Perspect. Med.* **2017**, *7* (2). DOI: 10.1101/cshperspect.a025262.
34. Weisberger, A. S.; Wessler, S.; Avioli, L. V. Mechanisms of Action of Chloramphenicol. *JAMA* **1969**, *209* (1), 97-103. DOI: 10.1001/jama.1969.03160140053011.
35. Schwarz, S.; Kehrenberg, C.; Doublet, B.; Cloeckaert, A. Molecular basis of bacterial resistance to chloramphenicol and florfenicol. *FEMS Microbiol. Rev.* **2004**, *28* (5), 519-542. DOI: 10.1016/j.femsre.2004.04.001.
36. Roberts, M. C.; Schwarz, S. Tetracycline and Chloramphenicol Resistance Mechanisms. In *Antimicrobial Drug Resistance: Mechanisms of Drug Resistance, Volume 1*, Mayers, D. L., Sobel, J. D., Ouellette, M., Kaye, K. S., Marchaim, D. Eds.; Springer International Publishing, **2017**; pp 231-243.
37. Svetlov, M. S.; Koller, T. O.; Meydan, S.; Shankar, V.; Klepacki, D.; Polacek, N.; Guydosh, N. R.; Vázquez-Laslop, N.; Wilson, D. N.; Mankin, A. S. Context-specific action of macrolide antibiotics on the eukaryotic ribosome. *Nat. Commun.* **2021**, *12* (1), 2803. DOI: 10.1038/s41467-021-23068-1.
38. Fyfe, C.; Grossman, T. H.; Kerstein, K.; Sutcliffe, J. Resistance to Macrolide Antibiotics in Public Health Pathogens. *Cold Spring Harb. Perspect. Med.* **2016**, *6* (10). DOI: 10.1101/cshperspect.a025395.
39. Floss, H. G.; Yu, T.-W. RifamycinMode of Action, Resistance, and Biosynthesis. *Chem. Rev.* **2005**, *105* (2), 621-632. DOI: 10.1021/cr030112j.
40. Gleckman, R.; Blagg, N.; Joubert, D. W. Trimethoprim: Mechanisms of Action, Antimicrobial Activity, Bacterial Resistance, Pharmacokinetics, Adverse Reactions, and Therapeutic Indications. *Pharmacotherapy* **1981**, *1* (1), 14-19. DOI: 10.1002/j.1875-9114.1981.tb03548.x.
41. Sköld, O. Sulfonamides and Trimethoprim. In *Antimicrobial Drug Resistance: Mechanisms of Drug Resistance*, Mayers, D. L. Ed.; Humana Press, **2009**; pp 259-269.
42. Fàbrega, A.; Madurga, S.; Giralt, E.; Vila, J. Mechanism of action of and resistance to quinolones. *Microb. Biotechnol.* **2009**, *2* (1), 40-61. DOI: 10.1111/j.1751-7915.2008.00063.x.

43. Redgrave, L. S.; Sutton, S. B.; Webber, M. A.; Piddock, L. J. V. Fluoroquinolone resistance: mechanisms, impact on bacteria, and role in evolutionary success. *Trends Microbiol.* **2014**, *22* (8), 438-445. DOI: 10.1016/j.tim.2014.04.007.
44. Menninger, J. R.; Coleman, R. A. Lincosamide antibiotics stimulate dissociation of peptidyl-tRNA from ribosomes. *Antimicrob. Agents Chemother.* **1993**, *37* (9), 2027-2029. DOI: 10.1128/aac.37.9.2027.
45. Leclercq, R. Mechanisms of Resistance to Macrolides and Lincosamides: Nature of the Resistance Elements and Their Clinical Implications. *Clin. Infect. Dis.* **2002**, *34* (4), 482-492. DOI: 10.1086/324626.
46. Cocito, C.; Chinali, G. Molecular mechanism of action of virginiamycin-like antibiotics (synergimycins) on protein synthesis in bacterial cell-free systems. *J. Antimicrob. Chemother.* **1985**, *16* (suppl_A), 35-52. DOI: 10.1093/jac/16.suppl_A.35.
47. Korczynska, M.; Mukhtar, T. A.; Wright, G. D.; Berghuis, A. M. Structural basis for streptogramin B resistance in <i>Staphylococcus aureus</i> by virginiamycin B lyase. *Proc. Natl. Acad. Sci. U. S. A.* **2007**, *104* (25), 10388-10393. DOI: 10.1073/pnas.0701809104.
48. Poole, K. Efflux pumps as antimicrobial resistance mechanisms. *Ann. Med.* **2007**, *39* (3), 162-176. DOI: 10.1080/07853890701195262.
49. Manzella, J. P. Quinupristin-dalfopristin: a new antibiotic for severe gram-positive infections. *Am. Fam. Physician* **2001**, *64* (11), 1863-1866.
50. Bozdogan, B.; Appelbaum, P. C. Oxazolidinones: activity, mode of action, and mechanism of resistance. *Int. J. Antimicrob. Agents* **2004**, *23* (2), 113-119. DOI: 10.1016/j.ijantimicag.2003.11.003.
51. Paukner, S.; Riedl, R. Pleuromutilins: Potent Drugs for Resistant Bugs-Mode of Action and Resistance. *Cold Spring Harb. Perspect. Med.* **2017**, *7* (1). DOI: 10.1101/cshperspect.a027110.
52. Schuster, S.; Vavra, M.; Kern Winfried, V. Efflux-Mediated Resistance to New Oxazolidinones and Pleuromutilin Derivatives in Escherichia coli with Class Specificities in the Resistance-Nodulation-Cell Division-Type Drug Transport Pathways. *Antimicrob. Agents Chemother.* **2019**, *63* (9), 10.1128/aac.01041-01019. DOI: 10.1128/aac.01041-19.
53. Ahmed, M. O.; Baptiste, K. E. Vancomycin-Resistant Enterococci: A Review of Antimicrobial Resistance Mechanisms and Perspectives of Human and Animal Health. *Microb. Drug Resist.* **2017**, *24* (5), 590-606. DOI: 10.1089/mdr.2017.0147.
54. Bayer, A. S.; Schneider, T.; Sahl, H.-G. Mechanisms of daptomycin resistance in Staphylococcus aureus: role of the cell membrane and cell wall. *Ann. N.Y. Acad. Sci.* **2013**, *1277* (1), 139-158. DOI: 10.1111/j.1749-6632.2012.06819.x.
55. Schneider, T.; Gries, K.; Josten, M.; Wiedemann, I.; Pelzer, S.; Labischinski, H.; Sahl, H. G. The Lipopeptide Antibiotic Friulimicin B Inhibits Cell Wall Biosynthesis through Complex Formation with Bactoprenol Phosphate. *Antimicrob. Agents Chemother.* **2009**, *53* (4), 1610-1618. DOI: 10.1128/aac.01040-08.
56. Rubinchik, E.; Schneider, T.; Elliott, M.; Scott, W. R. P.; Pan, J.; Anklin, C.; Yang, H.; Dugourd, D.; Müller, A.; Gries, K.; Straus, S. K.; Sahl, H. G.; Hancock, R. E. W. Mechanism of Action and Limited Cross-Resistance of New Lipopeptide MX-2401. *Antimicrob. Agents Chemother.* **2011**, *55* (6), 2743-2754. DOI: 10.1128/aac.00170-11.
57. Higgins, D. L.; Chang, R.; Debabov, D. V.; Leung, J.; Wu, T.; Krause, K. M.; Sandvik, E.; Hubbard, J. M.; Kaniga, K.; Schmidt, D. E.; Gao, Q.; Cass, R. T.; Karr, D. E.; Benton, B. M.; Humphrey, P. P. Telavancin, a Multifunctional Lipoglycopeptide, Disrupts both Cell Wall Synthesis and Cell Membrane Integrity in Methicillin-Resistant *Staphylococcus aureus*. *Antimicrob. Agents Chemother.* **2005**, *49* (3), 1127-1134. DOI: 10.1128/aac.49.3.1127-1134.2005.
58. Smith, J. R.; Roberts, K. D.; Rybak, M. J. Dalbavancin: A Novel Lipoglycopeptide Antibiotic with Extended Activity Against Gram-Positive Infections. *J. Infect. Dis. Ther.* **2015**, *4* (3), 245-258. DOI: 10.1007/s40121-015-0077-7.
59. Zhanel, G. G.; Schweizer, F.; Karlowsky, J. A. Oritavancin: mechanism of action. *Clin. Infect. Dis.* **2012**, *54 Suppl 3*, S214-219. DOI: 10.1093/cid/cir920.
60. Chowdhary, R.; Mubarak, M. M.; Kantroo, H. A.; ur Rahim, J.; Malik, A.; Sarkar, A. R.; Bashir, G.; Ahmad, Z.; Rai, R. Synthesis, Characterization, and Antimicrobial Activity of Ultra-Short Cationic β-Peptides. *ACS Infect. Dis.* **2023**, *9* (7), 1437-1448. DOI: 10.1021/acsinfecdis.3c00238.

61. Moore, M. J.; Qin, P.; Yamasaki, N.; Zeng, X.; Keith, D. J.; Jung, S.; Fukazawa, T.; Graham-O'Regan, K.; Wu, Z.-C.; Chatterjee, S.; Boger, D. L. Tetrachlorovancomycin: Total Synthesis of a Designed Glycopeptide Antibiotic of Reduced Synthetic Complexity. *J. Am. Chem. Soc.* **2023**, *145* (38), 21132-21141. DOI: 10.1021/jacs.3c08358.
62. Moore, M. J.; Qin, P.; Keith, D. J.; Wu, Z.-C.; Jung, S.; Chatterjee, S.; Tan, C.; Qu, S.; Cai, Y.; Stanfield, R. L.; Boger, D. L. Divergent Total Synthesis and Characterization of Maxamycins. *J. Am. Chem. Soc.* **2023**, *145* (23), 12837-12852. DOI: 10.1021/jacs.3c03710.
63. Li, P.; Wan, P.; Zhao, R.; Chen, J.; Li, X.; Li, J.; Xiong, W.; Zeng, Z. Targeted Elimination of bla (NDM-5) Gene in Escherichia coli by Conjugative CRISPR-Cas9 System. *Infect. Drug Resist.* **2022**, *15*, 1707-1716. DOI: 10.2147/idr.S357470.
64. Cubillos-Ruiz, A.; Alcantar, M. A.; Donghia, N. M.; Cárdenas, P.; Avila-Pacheco, J.; Collins, J. J. An engineered live biotherapeutic for the prevention of antibiotic-induced dysbiosis. *Nat. Biomed. Eng.* **2022**, *6* (7), 910-921. DOI: 10.1038/s41551-022-00871-9.
65. De, K.; DeStefano, M. S.; Shoen, C.; Cynamon, M. H.; Alley, M. R. K. 1704. Epetraborole, a Novel Bacterial Leucyl-tRNA Synthetase Inhibitor, Demonstrates Potent Efficacy and Improves Efficacy of Standard of Care Regimen Against Mycobacterium avium complex in a Chronic Mouse Lung Infection Model. *Open Forum Infect. Dis.* **2022**, *9* (Supplement_2), ofac492.1334. DOI: 10.1093/ofid/ofac492.1334.
66. Wang, Z.; Koirala, B.; Hernandez, Y.; Zimmerman, M.; Park, S.; Perlin, D. S.; Brady, S. F. A naturally inspired antibiotic to target multidrug-resistant pathogens. *Nature* **2022**, *601* (7894), 606-611. DOI: 10.1038/s41586-021-04264-x.
67. Wang, Z.; Koirala, B.; Hernandez, Y.; Zimmerman, M.; Brady, S. F. Bioinformatic prospecting and synthesis of a bifunctional lipopeptide antibiotic that evades resistance. *Science* **2022**, *376* (6596), 991-996. DOI: doi:10.1126/science.abn4213.
68. Mitcheltree, M. J.; Pisipati, A.; Syroegin, E. A.; Silvestre, K. J.; Klepacki, D.; Mason, J. D.; Terwilliger, D. W.; Testolin, G.; Pote, A. R.; Wu, K. J. Y.; Ladley, R. P.; Chatman, K.; Mankin, A. S.; Polikanov, Y. S.; Myers, A. G. A synthetic antibiotic class overcoming bacterial multidrug resistance. *Nature* **2021**, *599* (7885), 507-512. DOI: 10.1038/s41586-021-04045-6.
69. Chee, P. L.; Owh, C.; Venkatesh, M.; Periayah, M. H.; Zhang, Z.; Michelle Yew, P. Y.; Ruan, H.; Lakshminarayanan, R.; Kai, D.; Loh, X. J. Cationic Lignin-Based Hyperbranched Polymers to Circumvent Drug Resistance in Pseudomonas Keratitis. *ACS Biomater. Sci. Eng.* **2021**, *7* (9), 4659-4668. DOI: 10.1021/acsbiomaterials.1c00856.
